# Dynamics of the β-cardiac myosin auto-inhibited state explain cardiomyopathy pathogenesis

**DOI:** 10.1038/s41467-026-73572-5

**Published:** 2026-06-04

**Authors:** Daniel Auguin, Laurie Lannes, Carlos Kikuti, Nour Ayoub, Marie Juillé, Stéphane Réty, Neha Nandwani, Divya Pathak, Kathleen M. Ruppel, James A. Spudich, Julien Robert-Paganin, Anne Houdusse

**Affiliations:** 1 Structural Motility, CNRS UMR144, Institut Curie, Sorbonne Université, Université Paris Sciences et Lettres, Paris, France; 2 Laboratoire de Physiologie, Ecologie et Environnement (P2E), UPRES EA 1207/USC INRAE-1328, UFR Sciences et Techniques, Université d’Orléans, Orléans, France; 3 Laboratoire de Biologie et Modélisation de la Cellule, CNRS, UMR 5239, Inserm, U1293, ENS de Lyon, Université Claude Bernard Lyon 1, Lyon, France; 4 Department of Biochemistry, Stanford University School of Medicine, Stanford, CA US; 5 Stanford Cardiovascular Institute, Stanford, CA US

**Keywords:** Cryoelectron microscopy, Cardiovascular diseases

## Abstract

Cardiac contractility requires precise regulation. A recently discovered form of regulation of cardiac contractility involves a β-cardiac myosin off-state, the Interacting-Heads Motif (_Car_IHM). Despite its central role in cardiac physiology and disease, _Car_IHM structural dynamics remain poorly understood. Here, we integrate near-atomic resolution cryo-EM with all-atom molecular dynamics to characterize _Car_IHM in solution and in the context of the filament. We describe its conformational ensembles maintained by dynamic interfaces, and the stabilizing effect of the dilated cardiomyopathy mutation E525K, which limits S2 coiled-coil flexibility and impairs myosin activation. Intrinsically disordered regions of _Car_IHM and MyBP-C further modulate these dynamics. Our findings provide evidence for how _Car_IHM ensembles balance off/on states and anchor myosin heads in distinct thick filament environments. This integrated structural and dynamic approach enhances the understanding of thick filament regulation and facilitates predictions of the effects of genetic variants in inherited cardiomyopathies.

## Abstract (web summary)

This work reveals the intrinsic flexibility of the β-cardiac myosin off-state and its role in regulating the thick filament dynamics. It highlights that point mutations causing cardiomyopathy can disrupt this flexibility, resulting in impaired cardiac regulation

## Introduction

**Fig. 1:**
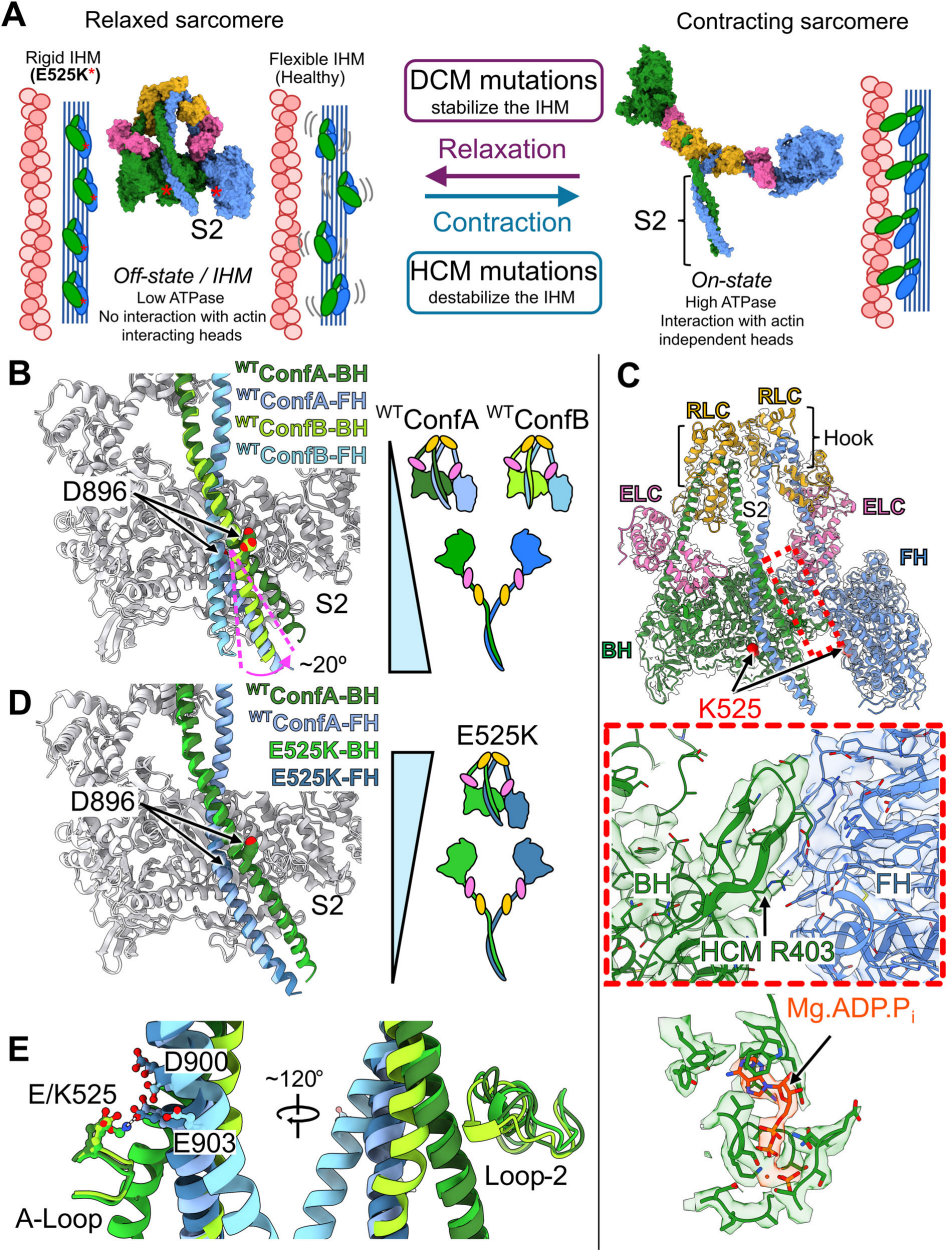
Wild-type and DCM E525K _Car_IHMs have distinct dynamics. **A** Regulation of cardiac myosin and effects of mutations. **B** The left panel shows superimposition of ^WT^ConfA and ^WT^ConfB focused on the BH motor domain (residues 1-710). A ~20° twist in the S2 position creates different interactions between the S2 and the BH. On the right panel, a cartoon illustrating that in WT, a low proportion of particles are in IHM, with two conformations solved at high-resolution. **C** High-resolution structure of E525K-_Car_IHM. The top panel presents the full structure, the middle panel zooms in on the BH/FH interface, and the bottom panel zooms in on the catalytic site of the BH. **D** E525K-_Car_IHM is highly similar to ^WT^ConfA: both structures are aligned over the entire _Car_IHM, (RMSD 0.95 Å calculated from all Cα atoms of the IHM heavy chains. On the right panel, a cartoon illustrates that, in E525K, only one main _Car_IHM conformation is observed and that a high proportion of particles adopt the IHM. (**E**) Comparison of the interaction between the ^BH^A-loop and the ^BH^Loop-2 with the S2 in the three structures. The color code is the same as in (**B**, **D**). IHM interacting-heads motif, A-loop activation-loop, BH blocked head, FH free head, ELC essential light chain, RLC regulatory light chain.

Rapid, cyclical contraction and relaxation of cardiac muscle is vital for pumping blood throughout the cardiovascular system. The ventricles relax in diastole as they fill with blood and then contract during systole to eject blood to the body. The high amount of force required during contraction is produced by myosins, with the β-isoform being predominant in human ventricles. The sarcomere is the fundamental contractile unit of cardiac and skeletal muscle. It consists of myosin-containing thick filaments interdigitated with actin-containing thin filaments. During systole, the sliding of the thick filaments against the thin filaments results in shortening of the sarcomere. During diastole, most β-myosin dimers adopt an ordered auto-inhibited off-state (Fig. 1A)^1–5^, which may be further stabilized by docking on the thick filament. The exact nature of these interactions is not known, although they are essential to tune the force produced by the heart. On the thin filament side, the myosin binding sites are masked during diastole, preventing myosin head interaction and promoting relaxation. Thin and thick filament-based regulation constitutes a dual control of the number of heads that participate in force production^6^, which is essential considering the spatial proximity between thick and thin filaments.

In this confined space, the regulation of contraction requires the precise proportion of myosins in the off-state vs. the on-state, which is key to tuning the force produced by the muscle during contraction, since the force of the ensemble is linked to the number of motors actively interacting with actin. The proportion of β-cardiac myosins in the off-state constitutes a reserve that can be mobilized only when needed (for example, as exertion increases) (Fig. 1A)^7–9^. The off-state is linked to an inactive, asymmetric structural state characterized by the folding back of the two myosin heads onto the proximal portion of their coiled-coil tail (Subfragment 2, or S2), called the Interacting-Heads Motif (IHM^10^). In 2023, the cryogenic electron microscopy (CryoEM) structure of human β-cardiac IHM (_Car_IHM) revealed essential information about the interfaces that contribute to off-state stability^5^ (Supplementary Fig. 1). This constituted a significant advance considering that the _Car_IHM structure could not be predicted and differs significantly from the off-state adopted by other muscle myosins and homology models created earlier^5^. The lack of atomic resolution structures and of knowledge about the dynamics of the interactions within the sarcomere constitutes a major obstacle to understanding the onset of force production in the heart and how missense mutations dysregulate the myosin on/off-switch^9^.

Two classes of inherited cardiomyopathies (CM) are linked to the alteration of the on/off-switch: hypertrophic and dilated cardiomyopathies (HCM and DCM, respectively). HCM is among the most common cardiovascular diseases, with a prevalence of ~1/500 in the general population^11^. HCM is characterized by early hypercontractility of the heart, leading over time to cardiomyocyte disarray, fibrosis, and thickening of the walls of the left ventricle^12^. In contrast, DCM hearts are hypocontractile, with dilation of the left or both ventricles. CMs can be caused by hundreds of single missense mutations in sarcomeric proteins^12–15^. About 40% of HCM cases^16^ and 1–5.3% of DCM cases are linked to single mutations in β-cardiac myosin^17^. The diverse locations of these mutations make the prediction of their effects difficult (e.g., refs. ^18–21^). Almost all HCM mutations tested reduce the stability of the off-state of myosin, thus increasing the number of active motors and therefore force production^9^. Conversely, the DCM E525K mutation was reported to stabilize the off-state, reducing the number of active heads^22,23^ (Fig. 1A).

Two structures of the relaxed cardiac thick filament were solved by CryoEM at medium resolution^24^ (6.4 Å) and by cryo-electron tomography (CryoET) at lower resolution^25^ (~20 Å). These two structures described the three-fold pseudo-symmetry of the fiber and how each crown of _Car_IHM interacts with distinct protein partners (including myosin binding protein C (MyBP-C) and Titin)^24,25^. However, the resolution of those two structures is insufficient to reliably model _Car_IHM conformations at the atomic level. Thus, predictions of the effects of CM-causing mutations on the intrinsic stability of _Car_IHM and its ability to dock on the filament are limited. This constitutes a major knowledge gap that prevents us from understanding how thick filament regulation affects cardiac function and how single missense mutations in the sarcomere can lead to disease.

In this work, we combine cutting-edge CryoEM data processing methods and all-atom molecular dynamics (MD) simulations as complementary approaches to describe with precision the dynamics of _Car_IHM. Instead of a set of rigid structures, we found a continuum of different IHM conformations that explain how WT _Car_IHM is prone to fast activation, and we describe the nature of the intrinsic dynamics of this off-state. In contrast, the DCM mutation E525K stabilizes the S2 coiled-coil position and restricts the _Car_IHM intrinsic dynamics. We also demonstrate that some _Car_IHM dynamics are essential for it to dock in different environments of the thick filament, allowing the stabilization of all crowns. In parallel, all-atom MD simulations were performed based on these _Car_IHM structures and successfully predicted the stabilizing/destabilizing effects of E525K and an HCM missense mutation, respectively. Finally, near-atomic resolution data allowed us to build a highly improved model of the human relaxed cardiac thick filament and enabled MD simulations. Overall, this integrative approach incorporating high-resolution data provides insights into thick filament-based regulation with major potential for predicting the consequences of missense mutations in inherited cardiomyopathies.

## Results

### _Car_IHM is an intrinsically dynamic set of asymmetric structures

The myosin off-state (_Car_IHM) has been known to be a particularly difficult structure to solve. Not only is it in equilibrium with heads that explore the on-state, but we have also found that it explores a continuum of different IHM conformations, providing clues as to how this state can dissociate upon activation. Such instability explains the low resolution of the previous CryoEM maps. Shortly after the publication of our original human β-cardiac myosin _Car_IHM structure^5^, software for processing data from flexible CryoEM samples became publicly available^26^. The three-Dimensional Flexible Refinement method (“3DFlex”)^26^ was applied to the WT-_Car_IHM dataset to analyze the _Car_IHM dynamics (see “Methods”, Supplementary Video 1). Among these, we solved the structures of two distinct conformations at high-resolution (Fig. 1B, Supplementary Fig. 2, and Supplementary Table 1). Among the 314,000 pre-selected particles previously used to solve PDB 8ACT, 106,681 particles were used for the reconstruction of Conformation A (^WT^ConfA) at 3.6 Å resolution, and 31,328 particles were used for the reconstruction of Conformation B (^WT^ConfB) at 3.8 Å resolution. For both structures, ^WT^ConfA and ^WT^ConfB, as well as the E525K-_Car_IHM structure (presented in the next section, Supplementary Fig. 3), the resulting high-quality CryoEM maps were significantly superior to those of previous studies^5,24^ (Supplementary Fig. 4A–C), allowing to position unambiguously the nucleotide (Mg.ADP.P_i_) and most side chains (Fig. 1C and Supplementary Fig. 5A–C). The motor domains of both the blocked head (BH: actin-binding site buried/unavailable) and free head (FH: actin-binding site available)^27^ (Supplementary Fig. 5D) of all three structures are in a similar conformation, including the position of the Converter and the closed P_i_-backdoor (Supplementary Fig. 5A–C). They adopt a stable PPS conformation (Supplementary Table 2) which traps ADP and P_i_ in the active site.

Unlike previous studies^5,24^, precise information on the side chains of the head/head interface is thus revealed for two conformations of _Car_IHM (^WT^ConfA and ^WT^ConfB). The CryoEM density maps unambiguously show that while the FH/BH interface involves the same structural elements in ^WT^ConfA, ^WT^ConfB, and E525K-_Car_IHM, distinct interactions occur (Supplementary Fig. 6 and Supplementary Table 3). In particular, the clear density of these maps indicates how the ^BH^R403 residue, which is associated with HCM when mutated to Gln^28^ can set up a network of plastic interactions at the FH/BH interface via different FH residues, providing direct evidence of the role of musical chairs dynamics^21^, with long, charged side chains facilitating intrinsic plasticity of interfaces, enabling the efficient formation and dissociation of contacts (Supplementary Video 2A). This plasticity seems correlated with the multiple orientations explored by the FH relative to the rest of the IHM. It differs by ~5° between ^WT^ConfA and ^WT^ConfB, the two WT structures we have solved among the continuum of conformations explored by _Car_IHM (Supplementary Fig. 7A, B). 3DFlex indicates that many orientations can be explored (Supplementary Video 1).

A much larger difference is observed for the contacts between the BH and the S2 coiled-coil (Fig. 1B and Supplementary Fig. 7C, D). An ~20° kink in the S2 occurs around residue D896 when ^WT^ConfA and ^WT^ConfB are compared (Fig. 1B). This indicates that in WT-_Car_IHM, the S2 can probe different positions by making variable contacts on the BH surface (Supplementary Table 3). This surface exploration provides a major source of dynamics for the WT-_Car_IHM (Supplementary Videos 1 and 2B–D).

### The DCM-causing E525K mutation greatly stabilizes _Car_IHM

Single ATP turnover experiments and FRET studies have shown that the E525K missense DCM mutation stabilizes the off-state^22,23,29^. To gain insights into the structural basis of this stabilization, we performed CryoEM studies on the dimeric 15-heptad heavy meromyosin (HMM) fragment of the mutant human β-cardiac myosin carrying human light chains (E525K-_Car_IHM, Supplementary Fig. 3). As per the previous WT-_Car_IHM cryoEM study^5^ (Fig. 1 and “Methods”), the experiment was performed without crosslinking to reveal the unbiased native dynamics of the auto-inhibited state. Once the dataset was cleaned of junk particles, all resulting 2D classes represented distinct views of the IHM. No class corresponded to the open HMM or individual heads. This differs from WT-_Car_IHM in which 24% of the particles corresponded to IHM. How this DCM mutation stabilizes _Car_IHM, however, could only be understood after the comparison between the above-described dynamic structures of WT-_Car_IHM and the conformational heterogeneity analysis of E525K-_Car_IHM detailed below.

The E525K particles were further purified by heterogeneous refinement, and the best class was refined to 3.02 Å resolution from 687,392 particles (Fig. 1C, Supplementary Figs. 3, 4A, B and Supplementary Table 1). This high-resolution map was then used to build the atomic model shown in Fig. 1C, which includes side chain information. In all maps from heterogeneous refinements of E525K-_Car_IHM, the S2 adopts the same position, while distinct orientations of the FH are clearly indicated (Supplementary Fig. 3). The 3DFlex method^26^ also indicates that similar degrees of heterogeneity were observed in the positions of the FH, the Hook and the lever arms across the WT and E525K datasets, while the E525K mutation significantly reduces the S2 movement compared to WT (Supplementary Videos 1 and 3). In the E525K-_Car_IHM structure, the S2 coiled-coil adopts a position close to that observed for ^WT^ConfA (Fig. 1D and Supplementary Fig. 7D). Introducing this single missense mutation located at the ^BH^Activation loop (A-loop) is sufficient to greatly stabilize _Car_IHM at the BH/S2 interface by formation of specific contacts between K525 and E903 and D900 (Fig. 1E and Supplementary Video 4).

### Role of BH/S2 interactions in the conformational ensembles explored by _Car_IHM: insights from high-resolution CryoEM maps

To explain the difference in stability between the WT- and E525K-_Car_IHM, we exploited the high-resolution CryoEM structures and examined the interactions found at the BH/S2 interface at the atomic scale. Analysis of the continuous conformational heterogeneity in the CryoEM data significantly improved the maps of WT-_Car_IHM, especially for the S2 coiled-coil. Compared to 8ACT^5^, >15 additional residues of S2 (L898-E920) are now modeled in two distinct orientations for ^WT^ConfA and ^WT^ConfB (Fig. 1B), and >25 residues (L898-E925) for E525K-_Car_IHM (Supplementary Fig. 4B, D). This part of S2 interacts with two surface loops of the BH (Fig. 1E): the A-loop (Supplementary Fig. 8A and Supplementary Video 2C) and Loop-2 (Supplementary Fig. 8B and Supplementary Video 2D). The flexibility of these myosin loops facilitates the establishment of transient interactions with S2 in the WT-_Car_IHM structures, providing a major source of conformational heterogeneity that is much reduced in the E525K-_Car_IHM dataset (Supplementary Video 2B–D). Weaker density is found for some S2 side chains despite the good map quality for the flanking domains for all three structures, reflecting musical chairs pairing at this BH/S2 surface, even for the E525K-_Car_IHM structure, in which the S2 position is restrained (Supplementary Video 3).

**Fig. 2:**
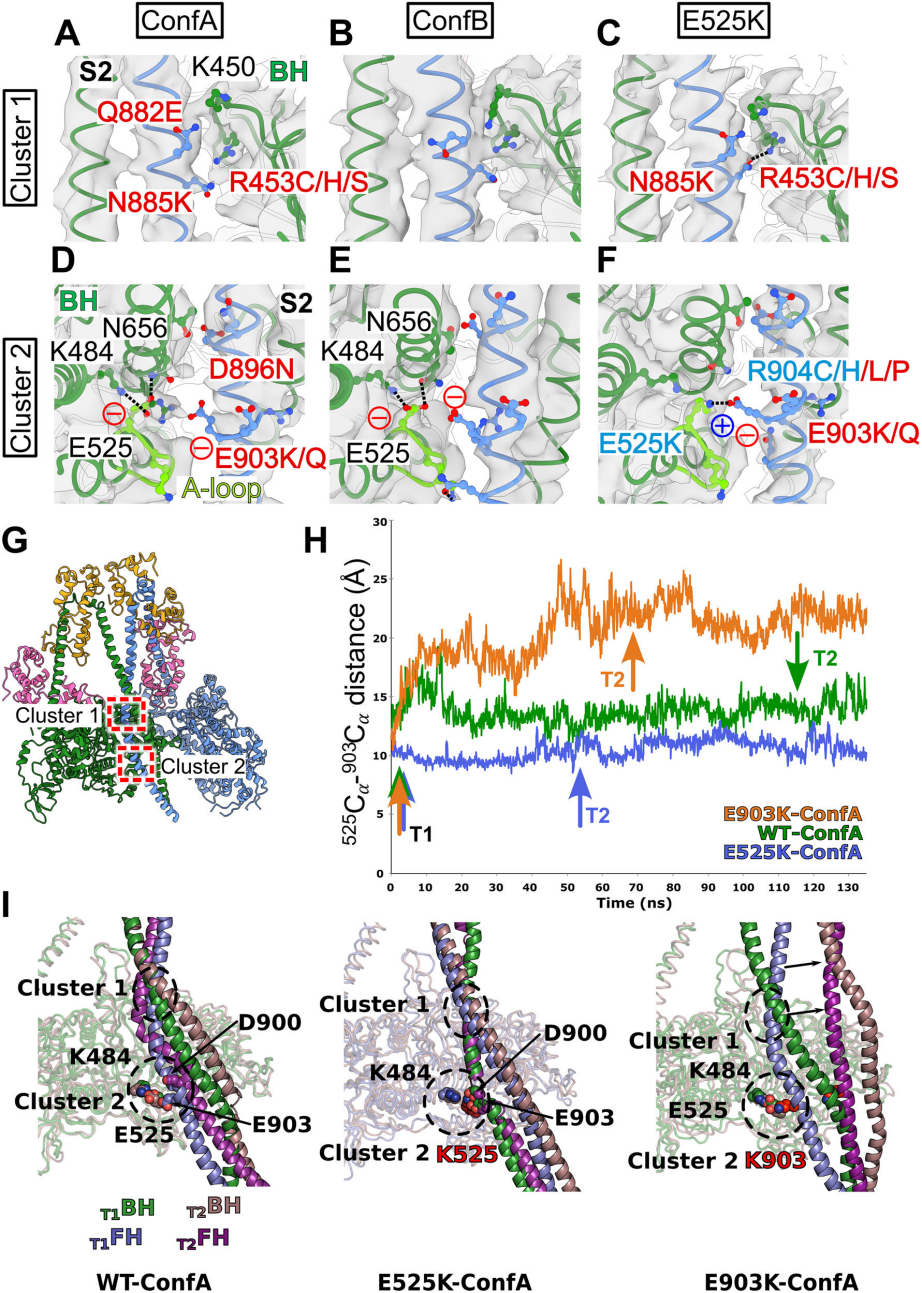
Dynamics of the S2 coiled-coil on the surface of the blocked head (BH) within the conformational ensembles of Wild-type (WT) and mutant _Car_IHM. The interaction of S2 with the BH is mediated by two clusters (1 and 2). **A–G** show the two charge clusters at the BH/S2 interface in the EM density map of Wild-type conformation A (^WT^ConfA), conformation B (^WT^ConfB), and E525K-_Car_IHM. A selection of residues are labeled when HCM or DCM mutations have been reported as pathogenic or likely pathogenic in ClinVar. HCM and DCM mutations are indicated in red and light blue, respectively. Mutations for R904 can either lead to HCM or DCM. In Cluster 2, charge of the residues at position 525 and 903 are shown to illustrate charge repulsions or attractions at the BH/S2 interface. **H** Results of the three all-atom MD simulations (WT-ConfA, DCM-E525K and HCM-E903K _Car_IHM) are shown by monitoring the ^525^C_α_-^903^C_α_ distance over time. **I** The position of the S2 at distinct representative times of the simulations are shown to illustrate the drastic differences in S2 stability when the missense E525K and E903K mutations are introduced starting from a ^WT^ConfA structure. The two times chosen for each condition, T1 and T2 are shown as arrows in panel (**H**).

The interactions stabilizing _Car_IHM have traditionally emphasized the significance of charge pairing in _Car_IHM formation and stabilization^29–31^. Indeed, pairing of negatively charged residues in Ring-1 (S2 residues E894 to D906) with positively charged areas of the BH motor domain^30^ and in Ring-2 (S2 residues E921–E935) with the FH motor domain have been described. We find instead that the native sequence of the S2 coiled-coil promotes labile interactions due to charge repulsion at the BH/S2 interface (Fig. 2A–G, Supplementary Table 3, and Supplementary Video 4). Our structures highlight the role of van der Waals (vdW) contacts and the existence of opposite charged residues on the BH/S2 interface, that explain the intrinsic instability of the S2 position in WT-_Car_IHM. These interactions involve the ^FH^S2 helix (Fig. 2A–G, Supplementary Table 3, and Supplementary Video 4) and occur in addition to weaker polar interactions between the ^BH^Loop-2 and Ring-1 residues of ^BH^S2 helix. The BH/S2 interactions were grouped into two clusters separated by a bend which occurs at residue D896 between ^WT^ConfA and ^WT^ConfB (Fig. 1B). Cluster 1 includes residues from ^FH^S2 (V878-N885) and a positively charged surface of the ^BH^Transducer, including a linker called the ^BH^HO-linker (residues 447–455) (Fig. 2A–C and Supplementary Table 3). Cluster 2 involves Ring-1 residues from ^FH^S2 with elements from the ^BH^A-loop and from the so-called ^BH^HW-helix (residues 647-664) (Fig. 2D–F and Supplementary Table 3). In both ^WT^ConfA and ^WT^ConfB, similar vdW contacts occur in Cluster 1 (Fig. 2A, B and Supplementary Table 3). Cluster 2 includes mainly vdW contacts and a few polar bonds, which differ between the two structures due to the distinct position of the S2 in each (Fig. 2D, E, Supplementary Table 3, and Supplementary Video 4). In ^WT^ConfA and ^WT^ConfB, E525 is close to the interface, but not directly involved in the contacts with S2, establishing a polar contact with ^BH^N656 and ^BH^K484 instead, as previously proposed by ref. ^32^. The DCM-causing mutation ^A-loop^E525K induces a charge reversal, altering the charge network at the ^BH^A-loop/S2 interface. This induces an electrostatic interaction between ^A-loop^K525 and ^S2^D900 and ^S2^E903 (Supplementary Fig. 8A), which changes the nature of the interactions compared to WT, not only in Cluster 2, but also in Cluster 1. Indeed, the two clusters are enriched in polar interactions in E525K-_Car_IHM compared to ^WT^ConfA and ^WT^ConfB (Fig. 2A–F and Supplementary Table 3).

The presence of missense mutations causing HCM or DCM in the two clusters demonstrates the importance of the interactions mediated by these clusters to finely control IHM stability (Fig. 2A–F and Supplementary Table 3). Predicting the effect of these mutations on the BH/S2 interface based only on the WT-_Car_IHM high-resolution structures is however, difficult due to the cooperative and labile nature of the interactions involved in IHM stability.

### Molecular dynamics simulations based on high quality CryoEM maps provide predictions on _Car_IHM stability

To further our understanding of the interaction network at the BH/S2 interface, we performed all-atom MD simulations starting from the high-resolution ^WT^ConfA structure. We also intended to test the predictive nature of MD regarding the effect of missense mutations on the stability of the BH/S2 interface and, therefore, on the sequestration of the myosin heads in the IHM. Two mutations in Cluster 2 were chosen for this study: the DCM mutation E525K, whose high-resolution structure is reported here, known to increase auto-inhibition^22,23^ and the HCM E903K mutation^33–35^, which is likely to cause motor dysregulation rather than dysfunction since it is not part of the motor domain (Fig. 2D–F). ^S2^E903 and ^A-loop^E525 are located on opposite sides of the BH/S2 interface (Supplementary Videos 2C, D and 3). In the E525K-_Car_IHM structure, ^S2^E903 establishes electrostatic interactions with ^A-loop^K525 (Figs. 1E, 2F and Supplementary Video 4). It is therefore intriguing that the reciprocal missense mutation E903K leads to an HCM phenotype, rather than a DCM phenotype like E525K. To dissect the effects of these single missense mutations, all-atom MD simulations were performed for 135 ns starting from the ^WT^ConfA structure, introducing a sidechain change for each mutant (see “Methods”) (Supplementary Video 5).

The overall root mean square deviation (RMSD) and fluctuations (RMSF) curves of the IHM motifs computed over time demonstrate that different behaviors are observed depending on the presence of mutations (Supplementary Fig. 9A, B). E525K is more stable than the WT condition, consistent with experimental CryoEM 3DFlex analysis (Supplementary Video 1). In contrast, the RMSD is significantly increased with E903K (Supplementary Fig. 9). Monitoring the position of the S2 on the BH surface also indicates that E525K stabilizes the S2 position, while E903K drastically decreases its stability (Fig. 2H, I and Supplementary Video 6). Thus, these MD simulations can predict the opposite effects on the stability of _Car_IHM structure depending on the presence of distinct missense mutations in Cluster 2. These results are remarkable as they are consistent with the clinical data that classified E525K and E903K as pathological DCM and HCM mutations, respectively^36,37^.

In detail, the in silico and CryoEM results are also consistent and complementary. The flexibility observed for each residue in the MD simulations (Supplementary Fig. 9C–E) matches the conformational variability captured by CryoEM data (Supplementary Fig. 7 and Supplementary Video 1). The MD simulations further allow visualization of the specific interactions that can occur at the _Car_IHM interfaces over time (Supplementary Videos 5 and 6). In particular, the interactions established between ^FH^Loop-2 and the S2 illustrate perfectly the concept of musical chairs: the flexible ^FH^Loop-2 forms a dynamic interface through a network of transient polar interactions. The transient nature of these interactions, combined with the flexibility of ^FH^Loop-2, makes it possible to maintain the global interface despite changes in the orientation of S2 (Supplementary Video 7). In the WT simulation, the S2 explores multiple positions within Cluster 2, forming only a few H-bonds (Supplementary Videos 5 and 6), which is consistent with the CryoEM results (Supplementary Video 1). While E525 is involved in electrostatic interactions with K484 and N656, residues ^S2^D900 and ^S2^E903 remain as free negative charges contributing to the flexibility of the S2 (Supplementary Fig. 9F). In the E525K simulation, K525 rapidly forms an interaction with ^S2^E903 promoting the formation of specific bonds between BH and S2 in both Cluster 1 and Cluster 2 (Supplementary Video 6), as observed in CryoEM maps. K525 introduces a positive charge that enables interactions with both ^S2^D900 and ^S2^E903, significantly reducing the flexibility of the S2 that remains in position (Fig. 2I, Supplementary Fig. 9B, E, G). In contrast, when the E903K mutation is introduced, major differences appear in both clusters, resulting in a modification of all contacts at the BH/S2 interface (Fig. 2H, I and Supplementary Video 6). In Cluster 2, S2 deviates in less than 40 ns from its initial position due to a lack of stabilization (Fig. 2H, I, Supplementary Videos 5 and 6). Although K903 introduces a positive charge on the S2, it cannot interact with E525 that is already involved in interactions with K484 and N656 (Supplementary Fig. 9H). A transient interaction between ^FH^K903 and ^BH^E653 occurs, but S2 continues to explore positions far from those of WT-_Car_IHM. The orientation and flexibility of the proximal S2, below the Hook and prior to Cluster 1, are also significantly affected (Supplementary Fig. 9E). This single E903K mutation results in a drastic disruption of the conformational ensembles explored by WT, which explains the destabilization of _Car_IHM. In contrast, E525K strongly biases the conformational ensemble to enrich it in conformations close to ^WT^ConfA. Our analysis shows how two mutations within the same region alter the local electrostatic network (Supplementary Fig. 9F–H), resulting in opposite effects on the stabilization of the S2 (Supplementary Videos 3, 5, and 6).

### The highly dynamic Cluster 3

**Fig. 3:**
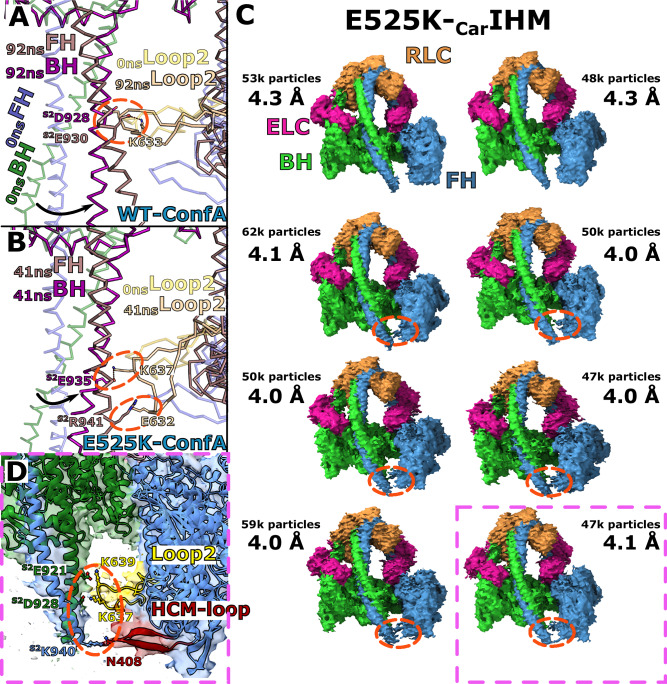
The dynamic Cluster 3 predicted by all-atom MD simulations and confirmed by CryoEM. **A**, **B**, two representative time points of the MD simulations of ^WT^ConfA and E525K are shown. The distal S2 (925–963) residues make no interaction at 0 ns. Transient interactions occur during the simulations (Supplementary Table 3 and Supplementary Video 8). Here, some interactions occurring at 92 ns for ^WT^ConfA and 41 ns for E525K are shown as example of Cluster 3 interactions. **C** The E525K CryoEM dataset was reprocessed with a series of local refinements and classifications (see “Methods” and Supplementary Fig. 10). One of the clusters from 3DVA was separated into 8 classes that show different conformations for S2, ^FH^Loop-2, and ^FH^HCM Loop. Orange rings outline Cluster 3 in each map. The resolution and the number of particles per class are shown. **D** Close-up of the EM density for Cluster 3 for the class boxed with pink dotted lines in (**C**). The distal S2, ^FH^Loop-2, and ^FH^HCM loop can be modeled to visualize the interactions that are formed. The ELCs are colored in pink, the RLCs are colored in yellow.

Besides the plasticity of the two previously mentioned clusters of interactions (Cluster 1 and Cluster 2), MD simulation of ^WT^ConfA and E525K both indicated that a third cluster – Cluster 3 – can occur between negatively charged S2 Ring-2 residues^30^ and the ^FH^Loop-2 (Fig. 3A, B, Supplementary Table 3, Supplementary Videos 5 and 8). Dynamic interactions formed rapidly, as a kink at residue A917 enabled the distal part of S2 (920-944) to approach the FH, while the proximal part of S2 maintained interactions with the BH without much change during the simulation.

We wondered whether interaction with ^FH^Loop-2 could be visualized experimentally by selecting CryoEM particles that would be most likely to exhibit interactions between actin-binding loops and S2. A series of local refinements and classifications in the CryoEM dataset of E525K-_Car_IHM resulted in eight classes of _Car_IHM maps (Fig. 3C, Supplementary Fig. 10 and “Methods”). The density in Cluster 2 is continuous and similar in all classes, while bending of S2 distal to Cluster 2 and different FH orientations are found among these eight classes. Thus, diverse FH/S2 interfaces are now distinguished among the E525K-_Car_IHM conformational ensembles (Fig. 3C). Two classes show density for both ^FH^Loop-2 and ^FH^HCM-loop, four classes only for the ^FH^Loop-2, and two classes show no density for these two loops since S2 is positioned further from the FH (Fig. 3C). Cluster 3 is thus highly dynamic, and the maps demonstrate the existence of distinct possible contacts between S2 and ^FH^Loop-2. A model was built and refined on the map showing the best density for Cluster 3 (Fig. 3D—see “Methods”). This indicates that Cluster 3 of the S2 can interact with both ^FH^Loop-2 and ^FH^HCM-loop (Fig. 3D). The conformations of both ^FH^Loop-2 and ^FH^HCM-loop vary between classes due to the flexibility of these FH loops and the presence of long side chains involved in dynamic interactions on both sides of Cluster 3. The labile nature of the FH/S2 interactions in Cluster 3 is consistent with the prediction of MD simulations (Fig. 3A, B, Supplementary Video 8).

The strength of our integrative approach is the accurate prediction of interactions using MD starting from a high-resolution structure, in which the residues are far from one another, allowing the interface to form during the simulation. Complementary to this approach, the analysis of the conformational ensembles physically imaged by CryoEM clearly demonstrates the existence of these dynamic interfaces. Both methods indicate that the interactions within Cluster 3 exhibit intrinsic dynamics, exemplified by the musical chairs phenomenon exhibited by ^FH^Loop-2 and ^FH^HCM-loop with various distal S2 Ring-2 residues, leading to formation and subsequent loss of this interface (Fig. 3C, Supplementary Video 3). Many single missense mutations found in Cluster 3, particularly within the distal S2 Ring-2, are associated with patients suffering from cardiomyopathies (Supplementary Table 3). The role of Cluster 3 in modulating _Car_IHM stability is significant, but it is not linked to simply establishing a stable interface, as previously presented^30,31^. Instead, our data show that this interface is formed by naturally transient, dynamic interactions that are precisely tuned by the S2 sequence, an essential condition to allow multiple conformations to be continuously explored. Many single missense mutations can indeed have a significant impact by altering the balance between the multiple conformations, leading to dysregulation of force production during heart contraction. The availability of heads participating in force production therefore, depends on the precise transient interactions that can occur in Cluster 3, as is also the case for the other dynamic interfaces that stabilize _Car_IHM.

### The Hook and S2 are critical to form the asymmetric _Car_IHM motif

**Fig. 4:**
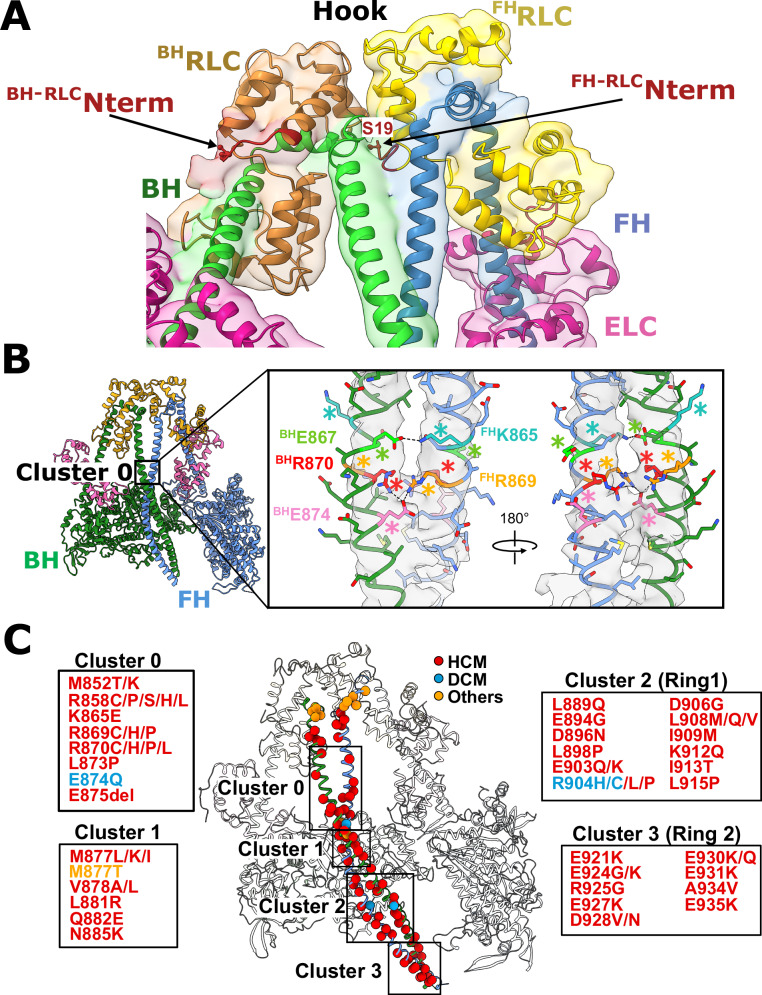
Asymmetry of S2 and the Hook in _Car_IHM. **A** The asymmetries of the RLC/RLC interface and of the coiled-coil induce different environments for each RLC. Model and map showing the Hook region and the proximal part of S2. The E525K-_Car_IHM map, enhanced in the RLC/RLC region (see “Methods”), is shown. In _Car_IHM, the ^BH^S2 residues are placed towards the C-terminus compared to the equivalent FH residues (see reporter residues represented by colored stars). The N-terminal extensions of the RLCs (^RLC^Nterm, residues 1-20) are not in the core of the interface. **B** Zoom-in of the residues of Cluster 0, illustrating the asymmetry of the internal interactions stabilizing the S2 bend (see reporter residues represented by colored stars, each pair of residues). **C** Pathogenic and likely pathogenic mutations (Clinvar) are labeled according to whether they cause hypertrophic or dilated cardiomyopathy (HCM or DCM). CM: undifferentiated cardiomyopathy. Note that several Variable of Uncertain Significance have also been identified in S2 (see Supplementary Fig. 12).

_Car_IHM corresponds to an asymmetric motif in which the BH and FH heads play complementary roles to create multiple interfaces. Despite the theoretical possibility that two distinct IHM motifs could form if either of the two heads could adopt the BH conformation (Supplementary Fig. 11), our high-resolution data demonstrate that only one of the two myosin heads can form the blocked head of the _Car_IHM motif and thus only one form of _Car_IHM is possible. This is a crucial point, because if two distinct _Car_IHM could form, only one of them would expose the appropriate asymmetric surface to allow docking on the surface of the thick filament, which is also asymmetric. This would impair regulation of the muscle by reducing the proportion of _Car_IHM structures that can be stabilized by docking onto the thick filament. Our analysis highlights the critical role of the Hook region in the formation and stabilization of this asymmetry. We used a custom mesh on 3DFlex^26^ to improve the map corresponding to the Hook region. In this map (Fig. 4A), the RLC/RLC interface is better defined and differs from previous models (Supplementary Video 9), with hydrophobic contacts offering less flexibility than the patterns observed in Clusters 1, 2, and 3 (Fig. 4A).

The structural characteristics of the Hook and the proximal S2 regions elucidate why a single type of _Car_IHM forms, characterized by an asymmetric configuration at the RLC/RLC interface. We identified several key points: (1) the rotation of the RLCs at the Hook promotes interactions between the first ^FH^RLC helix and the ^BH^S2 (residues L839-R845), without, however, allowing contacts between the ^BH^RLC and the ^FH^S2 (Fig. 4A). (2) Upstream of residue R845, the proximal S2 does not form a coiled-coil. Instead, the S2 side chains interact asymmetrically with the RLCs, stabilizing this interface^5^ (Fig. 4A). (3) The formation of the ^FH^RLC/ ^BH^S2 interface stabilizes a parallel proximal S2 coiled-coil (R845-F856), which is otherwise unstable^30^. (4) In addition, our structures highlight the asymmetric bend in S2 at residues K865-K876, involving residues from Cluster 0 (R858-K876, Fig. 4B). This bend is required for the formation of BH interactions with ^FH-S2^V878 and ^FH-S2^L881 in Cluster 1, as found in ^WT^ConfA, ^WT^ConfB, and E525K. Cluster 0 displays atypical internal residues at the d position of the coiled-coil (S866, R870), and is highly conserved in sequence (KSEARRKELEEK), although this S2 region is not involved in external interactions. The fact that missense mutations on the asymmetric S2 bend (Cluster 0) can result in cardiomyopathies (Fig. 4C and Supplementary Fig. 12) indicates that this region is not a simple connector that can be modified at will, but a stabilizing element of the asymmetric _Car_IHM structure (Fig. 4C and Supplementary Fig. 12).

With such characteristics in the S2 coiled-coil, the asymmetry established defines that only one head can reach the S2 and becomes the BH by establishing Cluster 1 and Cluster 2 interactions upon bending in Cluster 0. When Cluster 2 interactions form as found in ^WT^ConfA, interactions in Cluster 3 can also form downstream (toward the C-terminus) of the BH/S2 interface, further stabilizing distinct _Car_IHM conformations, depending on the orientation of the FH.

### Structure of the human cardiac relaxed thick filament enriched with near-atomic resolution data

The results from the above sections speak to the intrinsic stability of isolated _Car_IHM. In the sarcomere, interactions with other thick filament components also contribute to defining the precise balance between on and off states. Remarkable CryoET and CryoEM experiments distinguished the environments of three distinct _Car_IHM crowns: the horizontal Crown 1 (Cr1), the disordered Crown 2 (Cr2) and the tilted Crown 3 (Cr3) in the C-zone of the cardiac relaxed filament^24,25^ (Supplementary Fig. 13). Limits in resolution impede, however, knowledge of the nature of the interactions that stabilize the crowns and how these asymmetric motifs accommodate anchoring to different environments of the thick filament. We thus sought to optimize the thick filament structure by combining the EM density map^24^ (EMDB-29734) with our isolated near-atomic resolution _Car_IHM structures using short-term MD simulations and iterative real-space refinement (see “Methods”).

To evaluate how asymmetric _Car_IHM motifs dock onto the thick filament in distinct environments, we focused on Cr1 and Cr3, as the density attributed to Cr2 was of poor quality, most likely due to the increased rotational mobility of this crown. Initially, we docked the ^WT^ConfA and ^WT^ConfB structures, whose S2 orientations differ for Cluster 2, involved in BH binding (Fig. 1B). A good fit for S2 is observed for ^WT^ConfA for both Cr1 and Cr3, indicating that _Car_IHM conformations with Cluster 2 interactions similar to those observed for ^WT^ConfA or E525K are selected to dock both Cr1 and Cr3 on the rest of the thick filament (Supplementary Fig. 14A). In contrast, the S2 orientation of ^WT^ConfB does not match that observed in the filament (Supplementary Fig. 14A). The mobility of the S2 on the BH surface observed among the WT _Car_IHM conformational ensemble may in fact promote undocking of the crowns and their activation: when the ^WT^ConfB S2 is oriented along the axis of the thick filament, the contacts with the filament are lost (Supplementary Video 10). E525K would thus favor both _Car_IHM stability and its docking on all crowns of the thick filament.

**Fig. 5:**
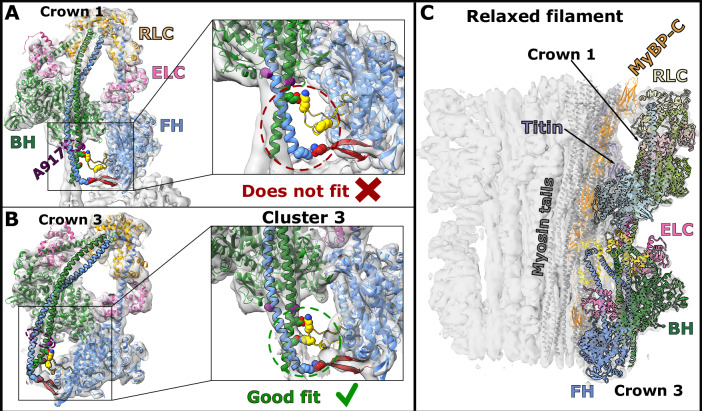
Docking of Cr1 and Cr3 and overall view of the refined atomic model of the thick filament. Density of Crown 1 (Cr1) (**A**) and Crown 3 (Cr3) (**B**) from the EMD-29734 map^24^ with the result of a rigid body fit of the _Car_IHM structure in which both ^FH^HCM and ^FH^Loop-2 are involved in interaction in Cluster 3 (as shown in Fig. 3D). A good agreement was obtained for the heads of the _Car_IHM motif for both Crown 1 and Crown 3 (correlation coefficient ~40%). For each fit, a zoom in Cluster 3 indicates that interactions do not occur in Cr1 (**A**) but are present in Crown 3 (**B**). A kink occurs in ^Cr3^S2 at position 917 but not in ^Cr1^S2 (dashed purple circle), allowing the distal part of ^Cr3^S2 to engage in interactions with FH. **C** Refined atomic model of the human relaxed cardiac thick filament describing how selected _Car_IHM conformations are able to dock in the Crown 1 and Crown 3 environments.

We next evaluated whether interactions in Cluster 3 contribute to docking. We used the _Car_IHM structure in which both ^FH^Loop-2 and ^FH^HCM-Loop are involved in interactions with S2 (Fig. 3D). The model fits well to the density of Cr3, but for Cr1, the distal part of S2 deviates from the density (Fig. 5A, B). The distal part of S2 thus differs in the two crowns downstream from A917 (Supplementary Fig. 14B). This suggests that the transient nature of Cluster 3 interactions (Fig. 3A–C) are important for the adjustment of the _Car_IHM motif to distinct environments: the involvement of the HCM-loop in Cluster 3 favors the docking of _Car_IHM in the Cr3 environment, but the fact that Cluster 3 interactions can easily be lost facilitates docking in the Cr1 environment and seems necessary for the undocking of the heads.

Once the best fit was found for the two crowns, the atomic models of MyBP-C, titin, and myosin coiled-coil tails of PDB 8G4L structure were added to obtain a model corresponding to a reconstructed filament architecture. Intermolecular interfaces, including flexible regions lacking experimental density, were completed without imposing initial contacts. The model was then optimized by cycles of real-space refinement against the 6 Å resolution EMD-29734 map^24^ and short-term MD runs (see “Methods”). The final model was analyzed to describe how Cr1 and Cr3 interact with the thick filament at the atomic level. It showed improved agreement with the EMD-29734 map (correlation coefficient 72% vs. 60% for 8G4L) (Fig. 5C). The quality of the geometry (Ramachandran favored, allowed, outliers 96.38, 3.54, and 0.08%) (Fig. 5C) allowed us to examine the internal dynamics of the crowns in their biological context (presented below). These results describe the realistic contacts that form when the crowns are docked (Supplementary Table 4), representing a significant advance required to evaluate how mutations may impact relaxation.

**Fig. 6:**
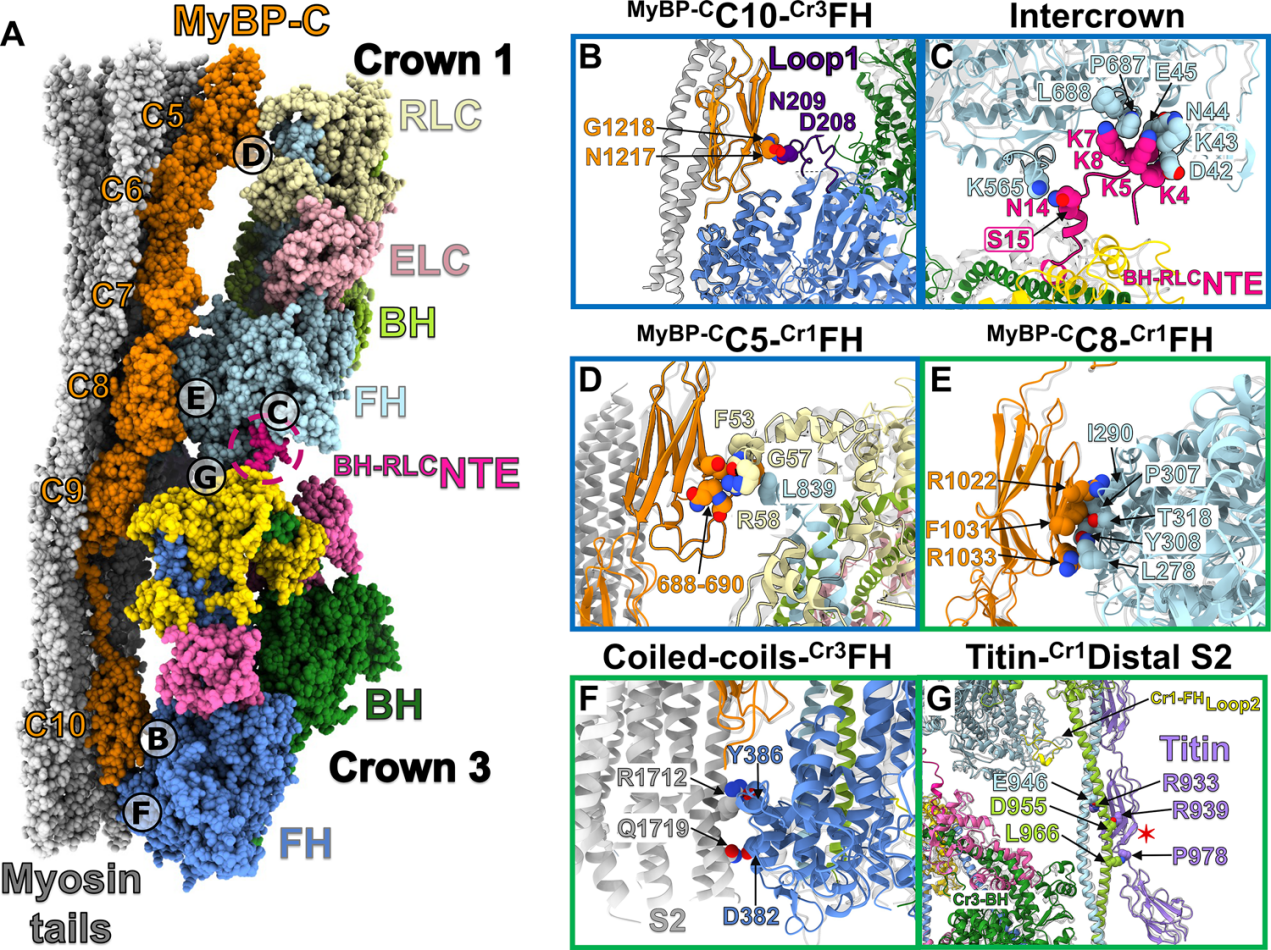
Interfaces stabilizing the crowns in the human cardiac relaxed filament model. **A** Overall model of the cardiac relaxed filament showing Crown 1 and Crown 3 (Cr1 and Cr3) in their environment. **B–G** Selected views of interfaces in the filament and how these were defined in the former PDB 8G4L^24^ (in transparent gray). Residues directly involved in interfaces are shown as spheres. **B–D** Interfaces that were not described in PDB 8G4L, now included in the filament model thanks to the approach combining the high-resolution structures of isolated _Car_IHM and short-term MD simulations. The phosphorylatable S15 from the N-terminal extension of the ^BH^RLC (^BH-RLC^NTE) is outlined in (**C**). This regulatory residue is not directly involved in interactions stabilizing the _Car_IHM, but is rather involved in the intercrown interface. **E–G** Examples of optimized interfaces that were previously suggested in PDB 8G4L. The filament model thus gains completeness and accuracy through descriptions of realistic interfaces at the atomic level. In (**G**), ^FH^Loop-2 does not form Cluster 3 interaction for Cr1 and S2 is involved in interactions with titin at this location. More distal residues of S2 (red star, 939–981), named Ring-3^30^ (Supplementary Table 4) also interact with titin.

Our reconstruction approach considers all intermolecular interfaces, including some that were not built in the initial low-resolution model (PDB 8G4L) (Fig. 6A–D and Supplementary Video 11). These interfaces comprise intrinsically disordered regions: the disordered loops of the myosin heads Loop-1 (aa 200–215) and Loop-2 (aa 625–643), the N-terminal extensions of the RLCs (^RLC^NTE, aa 1–27) as well as the MyBP-C specific disordered loop in C5, (aa 690–708), which interacts with the ^Cr1^FH-RLC (Fig. 6D and Supplementary Video 12). The involvement of Loop-1 of ^FH^Cr3 in the interaction with MyBP-C (^MyBP-C^C10/^FH^Cr3 interface) was thus revealed (Fig. 6B and Supplementary Table 4). One important improvement of the model concerns the RLC/RLC interface obtained from the high-resolution CryoEM data of the isolated _Car_IHM (Fig. 4A). This main difference from the PDB 8G4L model (Supplementary Videos 9 and 12) is significant as it impacts the prediction of contacts established by RLCs, and the position of the phosphorylatable Ser15 that regulates _Car_IHM stability (Fig. 6A, C). All interfaces were optimized, including those occurring at ^MyBP-C^C8/^Cr1^FH-U50, myosin coiled-coil tails (light meromyosin, LMM)/^Cr1^FH (primary head–head interaction site, PHHIS^8^), and Titin/^Cr1^distal S2 (aa 913-987) (Fig. 6E–G).

Among the large pool of possibilities present in the _Car_IHM conformational landscape, docking of _Car_IHM onto Cr1 and Cr3 requires the selection of different _Car_IHM conformations. For Cr1, the selected conformation is characterized by a ~10° rotation of the FH relative to ^WT^ConfA (Supplementary Fig. 14C), accompanied by small adjustments at the FH/BH interface and a different relative position of the Hook. This rotation likely results from its interaction with the C8 domain (Fig. 6A, E). In contrast, the conformation observed in Cr3 does not require such rearrangement and closely matches ^WT^ConfA, with no detectable differences: the establishment of Cluster 3 interactions, particularly those with ^FH^HCM-loop, appears sufficient to orient this _Car_IHM towards its docking site on the filament. These observations underscore the intrinsic dynamics of the _Car_IHM as a key determinant in enabling each crown to adopt specific conformations, essential to establish distinct contacts, highlighting the structural adaptability required for functional regulation of the thick filament.

### Dynamics within the relaxed thick filament and pattern of activation

We next performed all-atom MD calculations using the refined model of the thick filament to evaluate the stability of the interfaces and the dynamics of the crowns in their physiological context (Supplementary Video 11). The interactions established during the time course of the simulation were analyzed in detail and are presented in brown in Supplementary Table 4. The Titin/Cr1 coiled-coil (S2 cc) interface (Fig. 6G) involves polar interactions that are stable over time and maintains the Cr1 tail in position (Supplementary Table 4, Surface ID). In contrast, the interfaces involved in docking of the _Car_IHMs are plastic, allowing transient contacts and switching of residue partners (Supplementary Videos 13 and 14). The crowns are thus docked using several dynamic interfaces with the thick filament involving mainly their FH motor domains (Supplementary Table 5), facilitating establishment of a rapid equilibrium between on/off states of the myosin motors.

**Fig. 7:**
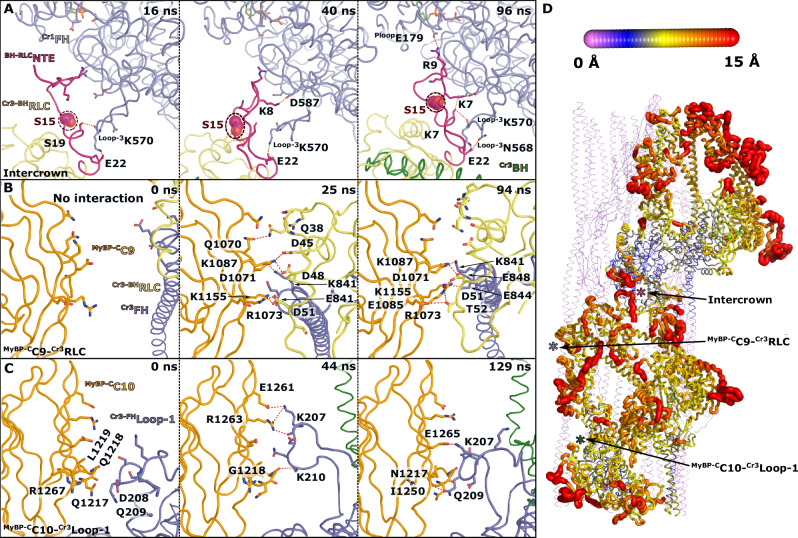
Dynamics of the human cardiac relaxed thick filament. All-atom MD experiments were performed during 140 ns starting from the near-atomic model of the filament containing Mg.ADP.P_i_ (see “Methods”). **A** shows different conformations of the N-terminal extension of the ^BH^RLC (^BH-RLC^NTE) of Crown 3 (Cr3). **B** shows how interactions between the ^Cr3-FH-^RLC and ^MyBP-C^C9 domain can form during the MD simulation when Cr3 slightly reorients as it is maintained close to the surface by intercrown interactions. **C** dynamic ^Cr3-FH^Loop-1/^MyBP-C^C10 interface. **D** displays the putty representation of the root mean square fluctuation (RMSF) of the blocked head (BH) and the free head (FH) of each crown (Cr1 and Cr3) during the course of the simulation. The mobility of the ^BH^Lever arm of Crown 1 (Cr1) is much greater than that of Cr3, due to interactions involving the ^Cr3-FH^RLC (intercrown and ^MyBP-C^C9).

The intercrown interface is the most dynamic and involves polar residues from the N-terminal extension (NTE) of the ^Cr3-BH^RLC (K7, K8, R9, and S19) interacting with different elements of the ^Cr1^FH, including residues from ^FH^SH3, ^FH^Loop-3 (e.g., N568, K570, D587), and E179 from the P-loop (Fig. 7A, Supplementary Video 15, and Supplementary Table 4). Interestingly, as is also the case for the RLC/RLC interface, the phosphorylatable S15 is not a main constituent of the intercrown interface during the simulation (Fig. 6C and Supplementary Video 15), indicating that _Car_IHM destabilization by phosphorylation occurs indirectly. During the simulation, as the ^Cr3-BH^NTE engaged in more intercrown interactions, establishing polar bonds such as ^Cr3-BH-NTE^R9 and ^Cr1-Ploop^E179, we detected six ephemeral contacts between ^Cr3-BH-RLC^S15 and ^Cr3-FH-RLC^E88 (lasting from 12–95 ns). Such an interaction would not occur if S15 were phosphorylated. In fact, the phosphorylated ^Cr3-BH-RLC^S15 could interact with ^Cr3-BH-RLC^K91 on the surface of its own RLC, thereby reducing the ability of the NTE to form intercrown contacts. The stability of both crowns would thus be affected by the ^BH^RLC phosphorylation in Cr3. This analysis indicates that Cr1 and Cr3 mutually stabilize their docking state if the RLC is not phosphorylated.

An additional interface between ^Cr3-FH^RLC and ^MyBP-C^C9 appears during the simulation, further contributing to the stabilization of Cr3 (Fig. 7B, Supplementary Table 4, and Supplementary Video 13). These contacts are likely promoted by the presence of intercrown contacts that restrain the positions of the ^Cr3^Hook on the surface of the filament, promoting interactions. Furthermore, Cr1 and Cr3 also form contacts between the ^Cr3-BH^ELC and ^Cr1-FH^Loop-3 and between the ^Cr3-FH^Converter and ^Cr1^Distal S2 region (Supplementary Table 4). The MD simulation thus illustrates how small changes in the orientation of these crowns can promote additional interactions, and it indicates that these two crowns can stabilize each other when they are both docked on the filament (Fig. 7B, C), in particular when the ^RLC^NTE is not phosphorylated, promoting formation of intercrown contacts (Fig. 7A).

As can be expected, RMSF curves of the heavy and light chains (Supplementary Fig. 15) reveal that flexibility is not homogeneous across crowns and indicate that the contacts made by the _Car_IHMs with their environment restrain their orientations and dynamics. The simulations were performed twice, revealing that these patterns are conserved in the second run (Supplementary Fig. 16). The interactions mediated by the ^FH^head constitute the primary interaction site for each crown. In contrast, ^Cr3^BH interacts with the filament via its Converter, its ELC and RLC, whilst ^Cr1^BH does not interact (Supplementary Table 5). The BH head orientation is thus more mobile than the FH. Fewer interactions are also mediated by the lever arms, and the contacts in Cluster 3 are more dynamic (Supplementary Videos 13 and 14). In addition, the ^Cr3-BH^lever arm shows reduced mobility compared to that of Cr1 due to stabilizing interactions, notably via the intercrown interactions that stabilize the Cr3 lever arm and Hook region (Fig. 7D). Cr3 appears more stable than Cr1, but this is likely biased by the additional constraints associated with the existence of intercrown contacts. These restraints contribute to increasing the stability of these two crowns compared to Cr2, which may explore more orientations due to the lack of stabilizing partners.

Overall, the nature of the contacts, their dynamics, and the stability of the surface recognized by the crowns differ and can all influence how long the heads remain docked. ^Cr1^FH is stabilized by multiple contacts involving ^MyBP-C^C5 and ^MyBP-C^C8 as well as coiled-coils of the filament core (Fig. 6D, E, Supplementary Videos 13 and 14), forming a multi-interface network that anchors the head. In contrast, ^Cr3^FH is stabilized differently, by interactions with ^MyBP-C^C10 (Fig. 6B and Supplementary Video 14) and the filament core. These findings indicate that the mesa surfaces (PHHIS, Transducer, Relay/Converter) differ in detail in how they contribute to either intrinsic _Car_IHM stabilization, docking of Cr1, or docking of Cr3 (Supplementary Table 5). The detailed analysis of the contacts observed during the MD simulation provides an unprecedented framework to understand how mutations might affect thick filament regulation and relaxation (Supplementary Table 4).

### Cardiomyopathy-causing mutations in the context of the filament

The extensive surfaces that stabilize _Car_IHM or contribute to its docking to the thick filament involve multiple residues. Yet, a single missense mutation, located in different places, can be associated with pathology. This underscores the precision of the regulation of myosin activation and function under physiological conditions. Most of the CM-associated variants have a dominant inheritance pattern. The probabilistic distribution of the MYH7 mutations in either BH or FH and on different Cr1, Cr2, or Cr3 leads to heterogeneity, repeated many times along the thick filament. Our study allows us to identify the role that a mutation could play when it occurs in these different environments (Supplementary Table 5). For example, mutations in the SH3 subdomain would primarily affect the intercrown interaction when present on Cr1 FH. They are unlikely to affect the motor function or stability of _Car_IHM. In addition, they are not involved in _Car_IHM stability or in docking if present on other heads. Interestingly, the HCM MYH7 E45 mutations to Asp, Gly, or Lys, classified as variants of unknown significance (VUS) for HCM, belong to this category (to date, no mutations of E45 are officially ascertained as pathogenic/likely pathogenic). Our study indicates that this residue is critical for the formation of the intercrown contacts (Fig. 6C and Supplementary Video 15) and thus might play a role in stabilizing Cr1 and Cr3 docking depending on RLC phosphorylation. The mechanisms by which some MYH7 VUS can impair function are thus suggested, although further studies are required to confirm these predictions.

As shown in Supplementary Table 5, most of the regions involved in Cr1 and Cr3 docking when they occur in FH are also important for _Car_IHM stability when they occur on the BH. Our simulations indicate how these regions can form distinct transient interactions. This is the case for ^FH^Loop-1, which is recruited to interact with MyBP-C C10 in Cr3 but can also interact with the ELC in the BH of all crowns to stabilize the kinked pliant region that primes the BH lever arm. Interestingly, the pathological mutations found to affect Loop-1 are located in regions that would alter its orientation, highlighting the importance of maintaining the loop’s native dynamics for myosin head sequestration. The mutations listed in Supplementary Tables 3 and 4 allow for the identification of _Car_IHM surface residues specifically involved in crown anchoring, without significantly impacting _Car_IHM stability. This analysis could prove useful for designing mutations for future experiments, enabling the evaluation of the role of thick filament anchoring in increasing _Car_IHM stability.

From analysis of the Clinvar database, we find that most pathogenic or likely pathogenic MYH7 mutations altering residues that can affect docking of the crowns correspond to internal residues close to the surface (blue highlights in Supplementary Table 4), with potential allosteric effects on the protein surface. These mutations are likely also impacting _Car_IHM stability and/or motor function. In addition, the few surface residues involved in docking can often contribute to _Car_IHM stability due to distinct interactions of that residue in both the BH and FH contexts. This is the case for the MYL2 D166 mutations to Tyr or Val that can impair docking of Cr1 on the C5 domain of MyBPC if present on the FH, but may also influence the stability of the RLC/RLC interface, if present on the BH. Thus, pathogenic MYH7 mutations that would only affect the docking of the crowns seem rare. The VUS of E45 cited above for affecting only intercrown interactions seems to be exceptions. Most mutations seem in fact to affect _Car_IHM stability, with potential effects on motor function. Further studies on S2 mutations in Cluster 3 will be of interest to evaluate their role in _Car_IHM stability, as well as their possible effects on the kinetics of formation of the BH configuration^29^, compared to their potential impact on the docking of either Cr1 or Cr3, which do not require the same residues for promoting their docking on the thick filament.

Predictions of how MyBP-C mutations in the C5-C10 subdomains affect docking of the crowns or the stability of MyBP-C on the thick filament are more straightforward. Based on the optimized model of the thick filament and on its MD simulations, we provide a detailed analysis of its interface with _Car_IHM and ^FH^RLC, providing a role for mutations of the C5 Loop and C9 subdomains whose interactions with _Car_IHM had not been previously modeled (Supplementary Tables 4,5, Fig. 7B, and Supplementary Videos 13, 14). In addition, the key residues involved in docking of each crown can be identified. The docking of Cr1 greatly depends on ^MyBP-C^C8 R1022, which leads to cardiomyopathy when mutated to His or Ser. Thus, affecting the Cr1 anchoring is sufficient to cause the disease, although this might also affect Cr3 if the role of intercrown in regulating cooperative docking of these crowns is confirmed.

## Discussion

Positioning molecular models, whether predicted or experimentally determined, within low-resolution CryoET and CryoEM maps offers significant insights into the formation of native assemblies in cellular environments. However, these low-resolution representations do not provide the detailed residue-level insights required for diagnostics and drug design. This study addresses this deficiency by integrating near-atomic resolution CryoEM structures of _Car_IHM with flexible refinement and all-atom MD within a lower resolution cryo-EM map of the relaxed thick filament. We thus provide (1) high-resolution structural information of WT-_Car_IHM, (2) how dynamics can be perturbed by single missense mutations, and (3) how _Car_IHM dynamics contribute to the docking of the myosin heads on different environments of the thick filament. The resulting improved model of the thick filament allows us to study the dynamic interactions formed to stabilize the myosin heads in relaxed conditions.

This integrative approach allows for the recovery of both structural and dynamic information regarding interfaces at the residue level within a physiological context. The findings indicate that the stability of _Car_IHM arises from a network of labile interactions capable of reconfiguration over short timescales, enabling quick transitions between inactive and active states, consistent with kinetics data^38^. Unlike a static structural stabilization of the off-state, dynamics of the _Car_IHM motif and in its interactions with the thick filament both concur to enabling rapid activation/deactivation of the heads, as required for the heartbeat.

**Fig. 8:**
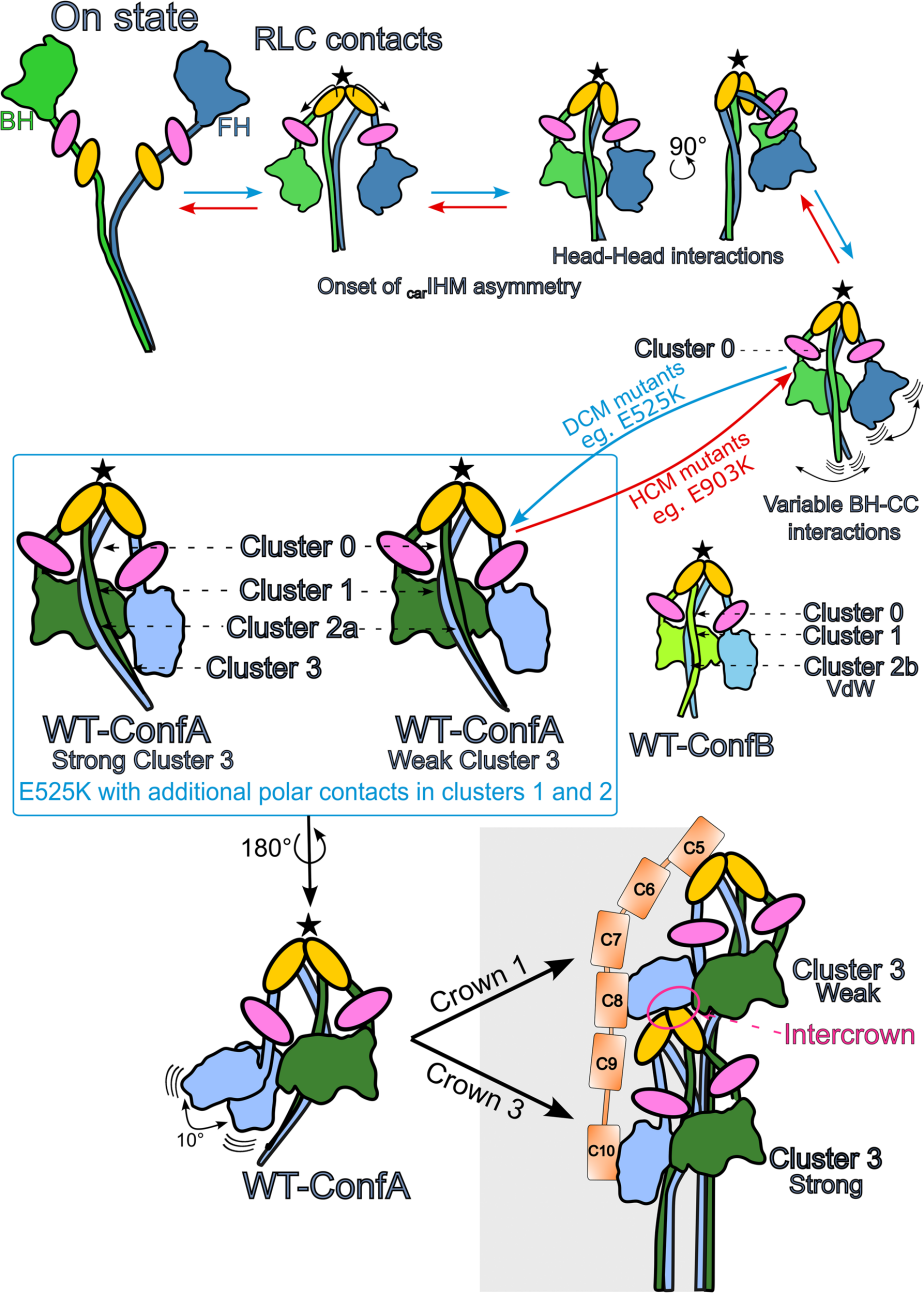
Proposed model for the formation of _Car_IHM and role of its dynamics for docking on distinct environment of the thick filament. Near-atomic resolution cryoEM structures indicate that the RLC dimerization drives IHM formation by imposing mechanical constraints that orient the lever arms in such a way to promote interactions of the two heads in an asymmetric fashion to form head/head interactions. Subsequent formation of the Clusters 0 promotes interactions of the coiled-coil with the BH. ConfA and ConfB differ mainly by the interactions in Cluster 2, and both retain flexibility in the FH orientation as well as in the orientations of the Hook region compared to the rest of the IHM. Different IHM conformations are selected thanks to this flexibility to promote docking in distinct crown-specific environments. MYH7 mutations can displace the ON-OFF equilibrium, leading to hypercontractility (HCM variants) or hypocontractility (DCM variants). The stability of Crown 1 and Crown 3 anchoring to the filament seems coupled depending on RLC phosphorylation. Indeed, intercrown interactions (pink circle) mediated by the NTE of the ^FH^RLC of Cr1 require that S15 is not phosphorylated so that it can create bonds with Crown 3.

The CryoEM maps of _Car_IHM (Fig. 3) provide a direct demonstration and clearer characterization of the role of the so-called musical chairs dynamics (as described from simulations by ref. ^21^). This concept highlights the role of long charged side chains in promoting a dynamic interface, such as the FH/BH interface in which ^BH^R403 alternate between ^FH^Y455 and ^FH^Q454, ^FH^R453 toggles between ^BH^N408 and ^BH^E409, and ^FH^H251 intermittently engages with ^BH^E409 (Supplementary Fig. 6 and Supplementary Video 2). Our integrative approach reveals that the intrinsic dynamics of _Car_IHM play a crucial role in docking capabilities across various environments. This is particularly evident in the regulation of interactions within Cluster 3, in which CryoEM data reveal that the S2 region may form distinct interactions with the ^FH^Loop-2 and ^FH^HCM loops. The formation of these interactions is vital for Cr3 docking; however, they only form occasionally when Cr1 is docked. The presence of ephemeral interactions at these dynamic interfaces facilitates the establishment of a balance between the on-state of the heads, which can be further regulated by their docking onto the thick filament (Fig. 8). Additionally, these transient interactions are essential for the rapid dissociation of the heads during Ca^2+^ influx and upon load sensing on the thick filament, allowing sufficient heads to achieve an on-state that enables effective force production once they interact with actin (Fig. 8).

Our precise modeling of the asymmetric RLC/RLC interface and S2 highlights their role in _Car_IHM formation. We reveal that this interface determines the formation of a single asymmetric _Car_IHM configuration. Pathogenic missense mutations for MYL2 support our model and indicate the crucial role of this interface for _Car_IHM stability^24^. We also show that the conservation of the charged residues of S2 (Ring-1 and Ring-2) induces dynamic interactions rather than stabilized interfaces (contrary to what was previously proposed^30,31^). We reveal the importance of Cluster 0 and Cluster 1 in _Car_IHM formation and stabilization (Fig. 4). We propose a model in which the formation of bonds between the RLCs would set the asymmetry in the motif and trigger formation of the proximal coiled-coil, inducing the capability for the S2 to bind to the BH once Cluster 0 is formed. Then distinct interactions via residues of Cluster 1 or Cluster 2 can occur leading to multiple dynamic _Car_IHM for the WT MYH7 sequence. Among these, ^WT^ConfB does not lead to the formation of interactions in Cluster 2 that are required for _Car_IHM docking to the thick filament, contrary to what is found for ^WT^ConfA. Thus, this continuum of possible dynamic _Car_IHM conformations can promote the detachment of the heads. Modeling and study of the dynamics of intrinsically disordered regions is essential for fully understanding how missense mutations can disrupt thick filament regulation. Thus, considering the dynamics is essential to fully account for how missense mutations can disrupt thick filament regulation, as it can explain the potential role of numerous VUS.

RLC phosphorylation has been shown to destabilize the off-state of purified human β-cardiac HMM^39^. Here we propose a mechanism by which RLC phosphorylation could further destabilize the off state by preventing intercrown interactions involving Cr1 and Cr3, which cooperatively stabilize the anchoring of the two crowns when not phosphorylated. Using our improved molecular models, the impact of missense mutations and post-translational modifications can be assessed with high accuracy through in silico simulations.

Kinetic and current structural studies indicate that not all heads are docked in relaxed myofibrils^6,40^, which is a critical question to understand how dual regulation works. Interestingly, our data indicate that WT _Car_IHM is not a single structure but explores multiple states of distinct stability, a subset of which would be adequate to allow docking on the thick filament (Fig. 3) in a very dynamic manner. We find that 24% of WT-IHM adopt the off-state on our grids. This is the number that has been evaluated through super-relaxed (SRX) state measurements in myofibrils, based on Mant-ATP displacement^41^. At first glance, this might suggest that _Car_IHM docking would not drastically change the equilibrium towards more heads prevented from cycling by further sequestration on the thick filament, indicating that the intrinsic _Car_IHM interactions are the most significant in setting up the rates and the equilibrium governing off-state formation (slow head cycling). However, this global measurement might be an average among multiple different behaviors of the crowns. Indeed, in the absence of mavacamten, the CryoET density of Cr3 appears better defined than that of Cr1, with Cr2 being the weakest^40^. Our model suggests that Cr1 and Cr3 may not dock with similar rates, as the _Car_IHM intrinsic bonds required for docking differ in the two crowns as well as the stability of the thick filament surface (MyBP-C, LMM, and titin docking surfaces involved). For example, the fact that the C5 subdomain may not stay stably in position^25,40^ could result in a lack of density for the BH of Cr1 due to additional orientational disorder. Further experimental studies probing how MyBP-C mutations may influence Cr1 docking can now be designed and interpreted more precisely thanks to the high-resolution models proposed by this study.

Mutations that disrupt local charge or steric complementarity alter the interaction network, making it either excessively rigid or labile. The E903K mutation weakens the BH/S2 interface and allows S2 to shift, thus increasing the likelihood of dissociation beyond normal limits and inducing HCM. The numerous manners by which a MYH7 missense mutation can result in HCM are correlated with the allosteric nature of myosin, possibly resulting in reduced stability of the PPS state required in both heads for _Car_IHM to form^42^. Fewer DCM mutations have been reported. The E525K mutation is one of them, and our study reveals why it belongs to the class of stabilizing _Car_IHM mutations. The mutation to Lys adds a positive charge to the BH/S2 interface, not only creating new interactions with D900 and E903 but also modifying the surrounding charge distribution. This reduces the conformational space explored by S2 and stabilizes the off-state without disrupting the FH mobility or Cluster 3 dynamics necessary for _Car_IHM anchoring to different thick filament environments. Thus, we predict that the mutation leads to a reduction in the number of heads that can participate in force production at the beginning of systole due to a slower opening of the _Car_IHM. However, the E525K mutation has been shown to enhance the intrinsic force produced by a crossbridge. Therefore, destabilization of E525K-_Car_IHM with activators such as Omecamtiv mecarbil or Danicamtiv would require finding the proper balance to avoid impairing relaxation, as both the drugs and the mutation slow the detachment of heads from the thin filament at the end of systole. The high-quality atomic models we provide, taking into account the multiple _Car_IHM conformations, will guide the development of essential drugs to expand the current therapeutic arsenal.

## Methods

### Production and purification of 15-heptad heavy meromyosin constructs

Recombinant WT and E525K mutant human β-cardiac myosin 15-hep HMM (res 1-942) were expressed and purified as described previously^29^. Briefly, myosin heavy chain (MYH7) was co-expressed with the human ventricular essential light chain (MYL3) carrying an N-terminal FLAG tag cleavable by TEV protease using adenoviruses in murine C2C12 myoblasts (ATCC) differentiated into myotubes; adenoviruses were generated in HEK293T cells (ATCC) using the AdEasy Vector System (Qbiogene Inc, Carlsbad, CA, USA) and purified by cesium chloride gradient centrifugation. MYH7 constructs contained a C-terminal GSG-RGSIDTWV affinity tag, and a GCN4 leucine zipper placed immediately before the C-t tag ensured dimerization of the two-headed constructs. Cells were harvested 96 hours post infection and immediately flash-frozen in liquid nitrogen (LN_2_). All steps for protein purification were carried out at 4 °C. Cell pellets were thawed, supplemented with 3 mM ATP, 1 mM DTT, 1 mM PMSF, Roche Protease Inhibitors, and 0.5% Tween-20, and lysed by Dounce homogenization. The clarified lysate was incubated with anti-FLAG resin, washed, and then the bound endogenous mouse RLC was depleted and replaced with His-tagged human RLC (purified from *E. coli*) as described previously^29^. Excess unbound RLC was washed away, and the resin was incubated with TEV protease overnight, cleaving the FLAG tag on ELC and His tag on RLC. The dissociated myosin in the supernatant was subjected to anion exchange chromatography. Fractions were pooled, and aliquots were flash frozen in LN_2_.

### Grid preparation

15-hep E525K-HMM was mixed with 0.5 mM Mg.ATP and let incubate for 15 min prior to the preparation of EM grids. Three µL of 15-hep E525K-HMM at 300 µg/mL (10 mM imidazole pH 7.5, 2 mM MgCl_2_, 0.5 mM Mg.ATP, and 8 mM NaCl) were applied on Lacey grids (200 mesh) beforehand glow discharged at −15 mA for 30 s (EasyGlow Pelco). The excess of sample was blotted for 1 s and immediately plunged-frozen in liquid ethane. A Vitrobot IV (ThermoFisher) robot plunger was used to prepare the CryoEM grid specimen.

### CryoEM data collection (E525K)

The grid was first screened on a 200-kV Glacios TEM (ThermoFisher) at the PICT platform at the Institut Curie and later sent to the European Synchrotron Radiation Facility for data collection with CM01 300-kV Titan-KRIOS TEM (ThermoFisher). CM01 was equipped with a Gaton K3 direct electron detector. The acquisition was supervised by the software EPU (ThermoFisher), and was performed at 105,000x magnification for a resulting pixel size of 0.84 Å. Each movie had 46 frames and received a total dose of ~50 e/Å^2^ (i.e., ~1.1 e/Å^2^/frame). A total of 28,398 gain-referenced movies were recorded with a defocus range set between −0.8 and −2.2 µm.

### E525K-_Car_IHM single particle analysis data processing

The processing of the data was entirely performed with CryoSPARC^43^starting with beam-induced motion correction (Patch Motion Correction) and contrast transfer function estimation (Patch CTF). Ten two-dimensional templates were generated based on the previous map of _Car_IHM to automatically pick particles (Template Picker). After curating exposures, inspection of the picks, 9.31 million down-sampled particles were extracted from 19,902 movies. Two rounds of 2D classification and selection filtered the pool of particles down to 3.73 million. A subset of pure particles was used to generate four ab initio 3D models. The full stack of particles was dispatched to the different models in the first heterogeneous refinement and further cleaned in a second round. Particles corresponding to all classes of _Car_IHM were input to 3DFlex pipeline^26^. The particles populating the best IHM-looking class (0.68 million) were reextracted and refined by non-uniform refinement to the resolution of 3.02 Å (FSC = 0.143).

After looking at the results from molecular dynamics, we wanted to look for the existence of Cluster 3 (see “Results”, section “The highly dynamic Cluster 3”). The results of E525K-_Car_IHM (Supplementary Video 1) 3DFlex analysis were used to investigate Cluster 3, as this contains more particles in conformations allowing for visualization of S2/FH interactions. Although 3DFlex indicates that E525K-_Car_IHM is less flexible than the WT, the mutant retains plasticity in the head-head region, with FH exploring different orientations (Supplementary Fig. 7A, B and Supplementary Video 1). The following procedure provided the 8 classes of E525K, showing the FH-BH contact Cluster 3 shown in Fig. 3 and explained in Supplementary Fig. 10: all the 1.5 million particles from the second round of heterogeneous refinement described above were first aligned by homogenous refinement, followed by local refinement focused on the BH. A mask around the FH was used in a subsequent 3D Variability Analysis (3DVA)^43^ whose Component 0 correlates with the butterfly movement of E525K-_Car_IHM as seen in the 3DFlex results (Supplementary Video 1). Particles from only one of the 3DVA clusters (416 k particles) were used in local refinement focusing on the FH, followed by 3D classification focusing on the BH. This result was obtained with a sacrifice in resolution: the final number of classes was defined as 8, as this was expected to provide around 50 k particles per class, global resolution varied between 3.9 and 4.4 Å depending on the class. Each of the maps shown in Fig. 3 is the result of a non-uniform refinement using each of the 8 classes from 3D classification.

Improvement of the map of the Hook region was obtained with the same set of 1.5 million particles and was rebalanced based on 3D alignment error. 645 k particles were kept. 3DFlex was run with a custom mesh comprised of 3 elements: FH + ELC, BH + ELC, and RLCs+S2. For the connectivity setup, the BH was chosen as pivotal point for the other two elements. Training was performed with 32 hidden units, latent centering strength 2.

To show the flexibility of the S2 (Supplementary Video 3 Part 2) the 1.5 million particles set was analyzed by 3DVA using a broad mask around the S2 only, Intermediates mode, 6 Å filter resolution.

### WT-_Car_IHM de novo data processing

The same 5154 movies collected at ESRF’s CM01 facility using a Titan Krios microscope (ThermoFisher) as described in ref. ^5^ were reprocessed in CryoSPARC^44^ using the following workflow: extraction of 3.2 mi particles with a 432 pixels box down sampled to 108 pixels (apix = 3.5 Å), 3 rounds of heterogeneous refinement for cleaning, re-extraction of 314,426 particles with a 336 pixels box down sampled to 288 pixels. This new particle set was analyzed by 3DFlex^26^ after real space cropping to 240 pixels, and Fourier cropping to 96 pixels (final apix 2.45 Å). A standard mesh with 20 tetra cells was applied, and the default 41 maps from Flex Generate were used for preparing the movies, as shown in Supplementary Video 1. Maps corresponding to the first and last frames of those movies were used in consecutive rounds of heterogeneous refinements until the two extreme conformations could be separated: 106,681 particles were used in a non-uniform refinement for obtaining Conformation A; 31,026 particles were used for Conformation B.

### Model building and refinement of the _Car_IHMs

The previously solved structure of _Car_IHM (PDB code 8ACT^5^) was fitted in each map: ^WT^ConfA, ^WT^ConfB and E525K with the tool fit in map from UCSF Chimera^45^. In each map, the structure was interactively refined into the map in Coot, using the function real space refine zone^46^. Steps of real space refinement were performed against the maps with the Phenix suite^47^. Between the steps, independent runs of minimization were performed with GROMACS (version 2018.3)^48^ to improve the statistics at the interfaces and reach realistic interactions in the regions that are less defined in the map. The CHARMM-GUI/Quick MD simulator module was used to rebuild and minimize the system^49,50^. In all the minimization steps, the model was relaxed in a box with explicit water (TIP3P), salt (150 mM KCl) in a box consisting of an edge of 232 Å, with the CHARMM36m force field^51^. A run of real space refinement was performed in the end. Such an approach combining real space refinement and independent runs of minimizations was already used on previous structures, including _Car_IHM structure and allows to significantly improve both the geometry and the fit in the CryoEM maps at various resolutions (e.g., refs. ^5,21,52^). For refinement statistics, see Supplementary Table 1.

### Fit in the map with improved density for the Hook region

To determine whether the phosphorylatable N-terminal extension of FH-RLC (^FH-RLC^NTE) is part of the RLC/RLC interface, we reprocessed the data to enhance the RLC/RLC region. The refined E525K-_Car_IHM model was fitted to the map. In parallel, Crown 3, including the heavy chains and the light chains, was extracted from the model of the relaxed human cardiac filament (PDB code 8G4L^24^) and fitted in the improved map using the entire motif or only the RLCs. The fits were performed with the function fit in map from the software UCSF Chimera^45^.

### General parameters used for all-atom molecular dynamics

For all the molecular dynamics experiments performed in this work, 500 ps NVT equilibration runs were performed (time step 0.001 ps with the integrator md) with GROMACS (version 2023.3)^48^. Restrains were set for backbone and side chains atoms to 400 kJ mol^−1^ nm^−2^ and 40 kJ mol^−1^ nm^−2^, respectively. Hydrogen bonds were constrained using the LINCS algorithm.

### All-atom molecular dynamics of isolated _Car_IHM

Three conditions were examined in all-atom MD simulations: WT-ConfA that started from the coordinates of the CryoEM structure ^WT^ConfA; E525K (E525K-ConfA) in which the side chain of residue E525 was manually changed for a lysine; and E903K (E903K-ConfA) in which the side chain of residue E903 was manually changed for a lysine. Each simulation was performed twice, to ensure the reproducibility of the observations (see the section “Reproducibility and Statistics”). The models were built and optimized using all-atom systems on CHARMM36m forcefields^51^. All the models were built in a box consisting of a cube with an edge 232 Å. The shortest periodic distance between the protein and the box was 5.1 nm at 0 ns. All simulations were performed in the same condition (150 mM KCl, pH 7.5 with one protonation of the histidine residue, system HSD) and contained the _Car_IHM model and TIP3 explicit water molecules. Long-range interactions were computed using the particle mesh Ewald (PME) method^53^, thus facilitating the calculation of the energies.

The simulations were performed using constant pressure and temperature ensemble (NPT). The temperature and the pressure of the system were fixed at 310.15 K with the Nosé-Hoover thermostat and 1 bar with the Parrinello-Rahman barostat^53,54^. The MD production time step was set to 2 fs (dt = 0.002 ps) for them all. WT-ConfA, E525K and E903K systems contain 1,242,557; 1,258,385 and 1,226,383 atoms, respectively. The simulations were performed over 135 ns with GROMACS (version 2023.3)^48^.

### Model building and refinement of the model of the filament at near-atomic resolution

The model of the filament was built based on the CryoEM medium resolution structure of the relaxed thick filament bound to Mavacamten (PDB code 8G4L)^24^ and on the high-resolution CryoEM structure of _Car_IHM ^WT^ConfA. Two copies of ^WT^ConfA were fitted in the CryoEM density of the relaxed human filament containing Crown 1 (CrH in ref. ^24^) and Crown 3 (CrT) with Chimera^45^ and ChimeraX^55^(“fit in map module”) (EMDB-29734). All the starting coordinates for other elements, such as the coiled-coil constituting the filament or MyBP-C and Titin, come from the model 8G4L. The missing structural elements, such as the loop formed by residues 686-711 from the C5 domain of MyBP-C, myosin Loop-1 (residues 200–215), or the N-terminal extension of the RLC (residues 1–23), were rebuilt with Modeller (v 10.1)^56^ provided with Chimera. The system was built and optimized with QuickMD simulator from CHARMM-GUI^49,50^. An initial step of 1 ns simulation was performed, the model was relaxed in a box consisting with a cube of an edge of 365 Å and a volume of 365 Å^3^, with explicit water (TIP3P) with the CHARMM36m force field^51^, salt (150 mM KCl, pH 7.5). The shortest periodic distance between the protein and the box was 2.17 nm at 0 ns. During this run, the backbone of the filament was restrained, keeping the Cα of the entire model maintained with constraints of 400 kJ mol^−1^ nm^−2^, except for the flexible interfaces that were free. This allowed computing of realistic interfaces in these regions that were not rebuilt in the initial model 8G4L^24^, although they are part of the interfaces.

The system was built and optimized with QuickMD simulator from CHARMM-GUI^49,50^. An initial step of 1 ns simulation was performed, the model was relaxed in a box consisting of an edge of 365 Å and a volume of 365 Å^3^, with explicit water (TIP3P) with the CHARMM36m force field^51^, salt (150 mM KCl, pH 7.5). GROMACS (version 2023.3)^48^. During this run, the backbone of the filament was restrained, with the exception of the flexible interfaces: loop 686-711 from the C5 domain of MyBP-C; myosin Loop-1 (residues 200-215) or the N-terminal extension of the RLC. This allowed the computing of realistic interfaces in these regions that were not rebuilt in the initial model 8G4L^24^.

Steps of real-space refinement were performed against the map in the Phenix suite. Between the steps, independent runs of minimizations were performed to improve the statistics at the interfaces (head/head, cc/head, Hook region, and intermolecular interfaces with partners) with the same methodology as described for isolated _Car_IHM. A final run of real space refinement was performed in the end, allowing reaching high-quality geometry (Ramachandran 96.38; 3.54 and 0.08% for the favored, allowed, and outliers angles, respectively) and a correlation coefficient of 69% between the model and the map.

### All-atom molecular dynamics of the cardiac relaxed filament

The starting point for the all-atom MD simulations performed on the filament was based on the atomic coordinates of the relaxed filament. The model was built and optimized using CHARMM36m forcefield (software CHARMM-GUI)^49–51^. The system was built using a box consisting of a cube with an edge of 365 Å and a volume of 365 Å^3^. The shortest periodic distance between the protein and the box was 2.17 nm at 0 ns. It contained all atoms of the thick filament, TIP3 waters with 150 mM KCl, pH 7.5, and one single protonation of the histidine (HSD form). Thus, the long-term simulation was performed using 4,613,271 atoms, 310.15 K of temperature and 1 bar of pressure. The simulation was performed with GROMACS (version 2023.3)^48^ for 140 ns. During minimization, to maintain the overall structure of the filament, constraints of 40 kJ mol^−1^ nm^−2^ were kept, maintaining the Cα of MyBP-C, Titin, and the coiled-coils constituting the core of the filament. Crowns 1 and 3, including the heavy chains, the ELCs, and RLCs, were free of constraints. The simulations were performed twice to ensure the reproducibility (see Statistics and Reproducibility).

### Trajectory analysis

Trajectories were generated and computed with gmx trajconv from Gromacs. Frames of 0.1 ns steps were extracted from the original recorded simulations (sampled at 10 ps) for the analysis. All the trajectories were adjusted on the first frame by a superimposition on the backbone of the motor domains (1–710) for _Car_IHMs and on the backbone of MyBP-C, Titin, and the contextual coiled-coils in the filament, using the macrocommand of VMD (RMSD trajectory tool, version 1.9.4a53, June 29, 2021), this allowed the extraction of the RMSD plots. RMSF were obtained with MDAnalysis^57^ and further used to compute the sausage putty representations^58^ in PyMOL.

All trajectories were analyzed in detail to describe the time evolution of the interfaces. For each interface presented, representative frames illustrating this evolution were selected and assembled into movies (Supplementary Videos 7, 13–15). Interactions observed in the molecular dynamics simulations but not present in the initial models are highlighted in brown in Supplementary Table 4.

### Statistics and reproducibility

All trajectories generated by molecular dynamics simulations (isolated _Car_IHM and relaxed filaments) were performed in duplicate to ensure reproducibility. The RMSD values obtained from the two replicates for the three isolated _Car_IHM (WT, E525K, and E903K) are presented in Supplementary Data 1. Both sets of simulations led to the same conclusions: (i) E903K is more dynamic than WT and E525K, and (ii) the mutations alter the charge network of Cluster 2, with K525 stabilizing the coiled-coil position by establishing electrostatic interactions with negatively charged residues located on S2 (Supplementary Data 1).

Simulations of the cardiac relaxed thick filament were also performed in duplicate to ensure reproducibility. In these simulations, the flexible interfaces (loop 686–711 from the C5 domain of MyBP-C, myosin Loop-1 (residues 200–215), and the N-terminal extension of the RLC) were rebuilt independently in each replicate (Supplementary Fig. 16, Supplementary Data 3 and 4). Raw RMSD and RMSF data from both simulations are provided in Supplementary Data 2–4, and both simulations led to the same conclusions.

### Reporting summary

Further information on research design is available in the Nature Portfolio Reporting Summary linked to this article.

## Supplementary information

Supplementary Information

Description of Additional Supplementary Files

Supplementary Data 1

Supplementary Data 2

Supplementary Data 3

Supplementary Data 4

'Supplementary Video 1'

Supplementary Video 2

Supplementary Video 3

Supplementary Video 4

Supplementary Video 5

Supplementary Video 6

Supplementary Video 7

Supplementary Video 8

Supplementary Video 9

Supplementary Video 10

Supplementary Video 11

Supplementary Video 12

Supplementary Video 13

Supplementary Video 14

Supplementary Video 15

Reporting Summary

Transparent Peer Review File

## Source data

Source Data

## Data Availability

The atomic model generated in this study have been deposited in the PDB^60^ under accession codes: 9TPL 10.2210/pdb9TPL/pdb (^WT^ConfA); 9TPK 10.2210/pdb9TPK/pdb (^WT^ConfB); 9TPJ 10.2210/pdb9TPJ/pdb (E525K). The cryoEM map of of ^WT^ConfA, ^WT^ConfB, E525K have been deposited in the EMDB database^61^ under the accession numbers EMD-56108 https://www.ebi.ac.uk/pdbe/entry/emdb/EMD-56108 (^WT^ConfA); EMD-56107 https://www.ebi.ac.uk/pdbe/entry/emdb/EMD-56107 (^WT^ConfB); EMD-56106 https://www.ebi.ac.uk/pdbe/entry/emdb/EMD-56106 (E525K), respectively. The CryoEM dataset collected on E525K is available at the ESRF as Lannes, L. (2027). CryoEM-BAG France: Structural Biology using cryoEM in France [Dataset]. European Synchrotron Radiation Facility under [doi.org/10.15151/ESRF-ES-1521652352]. The source data underlying Fig. 2H, Supplementary Fig. 9A, B, Supplementary Fig. 15 A–F, and Supplementary Fig. 16A–F are provided as Source Data file. Source data are provided with this paper.
