# Supplementary material for: Dynamics of the β-cardiac myosin auto-inhibited state explain cardiomyopathy pathogenesis
Source: Nat Commun. 2026 Jun 4;17:5502. doi: 10.1038/s41467-026-73572-5 (PMC13287786; doi:10.1038/s41467-026-73572-5)
Supplement: Supplementary file 1 — Supplementary Information [file 41467_2026_73572_MOESM1_ESM.pdf]

Dynamics of the β-cardiac myosin auto-inhibited state explain cardiomyopathy pathogenesis

Daniel Auguin[1,2]*, Laurie Lannes[1]*, Carlos Kikuti[1]*, Nour Ayoub[1], Marie Juillé[1], Stéphane Réty[3], Neha Nandwani[4,5], Divya Pathak[4,5], Kathleen M. Ruppel[4,5], James A. Spudich[4,5], Julien Robert-Paganin[1,#], Anne Houdusse[1,#,%].
* These authors contributed equally,
# These authors jointly supervised this work

%Corresponding author: anne.houdusse@curie.fr ORCID : 0000-0002-8566-0336

Supplementary information

Supplementary Figures

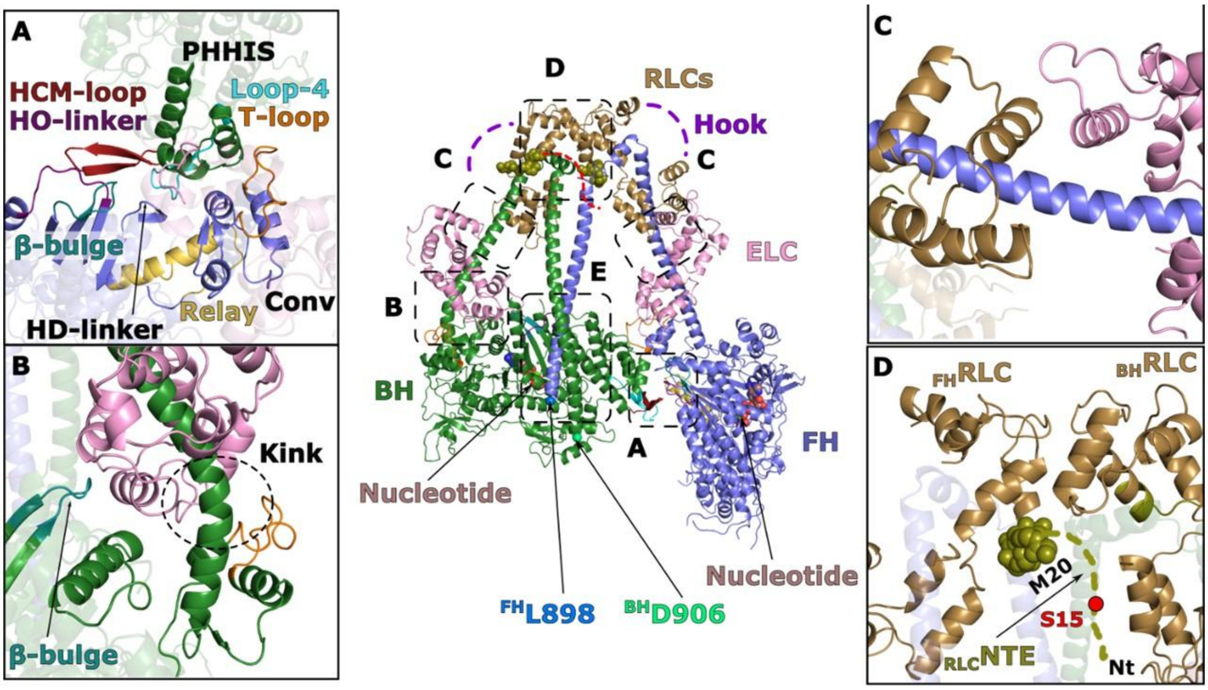

Supplementary Figure 1 – Interfaces stabilizing ${}_{Car}$IHM. β-cardiac myosin consists of two heavy chains (green and blue), each binding an essential light chain (ELC, pink) and a regulatory light chain (RLC, yellow). The first structure of the human cardiac interacting-heads motif (${}_{Car}$IHM) was previously solved at 3.8 Å resolution by CryoEM (PDB code 8ACT[1]). The ${}_{Car}$IHM motif is asymmetric. It is characterized by a blocked head (BH) and a free head (FH) interacting together and folding back on the S2 coiled-coil (S2). The structure revealed how the double-headed folded-back conformation stabilizes the inactive state through five main interfaces. **(A)** The head-head interface, involving actin-binding elements (^{BH}HCM-loop, ^{BH}Loop-4), the Primary Head-Head interaction site (^{BH}PHHIS), elements of the transducers of both heads (HO-linker, β-bulge) and elements of the Converter (^{FH}Top-loop/^{FH}T-loop). **(B)** The BH/^{BH}ELC interface established due to a kink in the BH lever arm. **(C)** The ELC/RLC interface in the lever arms of both heads. **(D)** The ^{BH}RLC/^{FH}RLC interface at the Hook region, located at the end of the lever arm and defined as a flexible hinge[2]. Unlike what has been described in the smooth muscle myosin IHM[3–5], the phosphorylatable N-terminal extension of the ${}_{FH}$RLC (${}_{RLC}$NTE) is not directly part of the ^{BH}RLC/^{FH}RLC interface. Indeed, a large part of the NTE interface is disordered in ${}_{Car}$IHM (in red dotted lines). **(E)** The BH/S2 interface also plays an important role for stabilizing the OFF-state, however heterogeneity in the positioning of the end of the S2 coiled-coil prevented the visualization of the interactions after the residues ^{BH}898 and ^{FH}D906 in this first model (PDB 8ACT). A close-up of **(E)** is not presented here, as this interface was flexible and not described in detail in the previous work[1]. In the present study, the improved quality of the ${}_{Car}$IHM structures enables us to better define this interface.

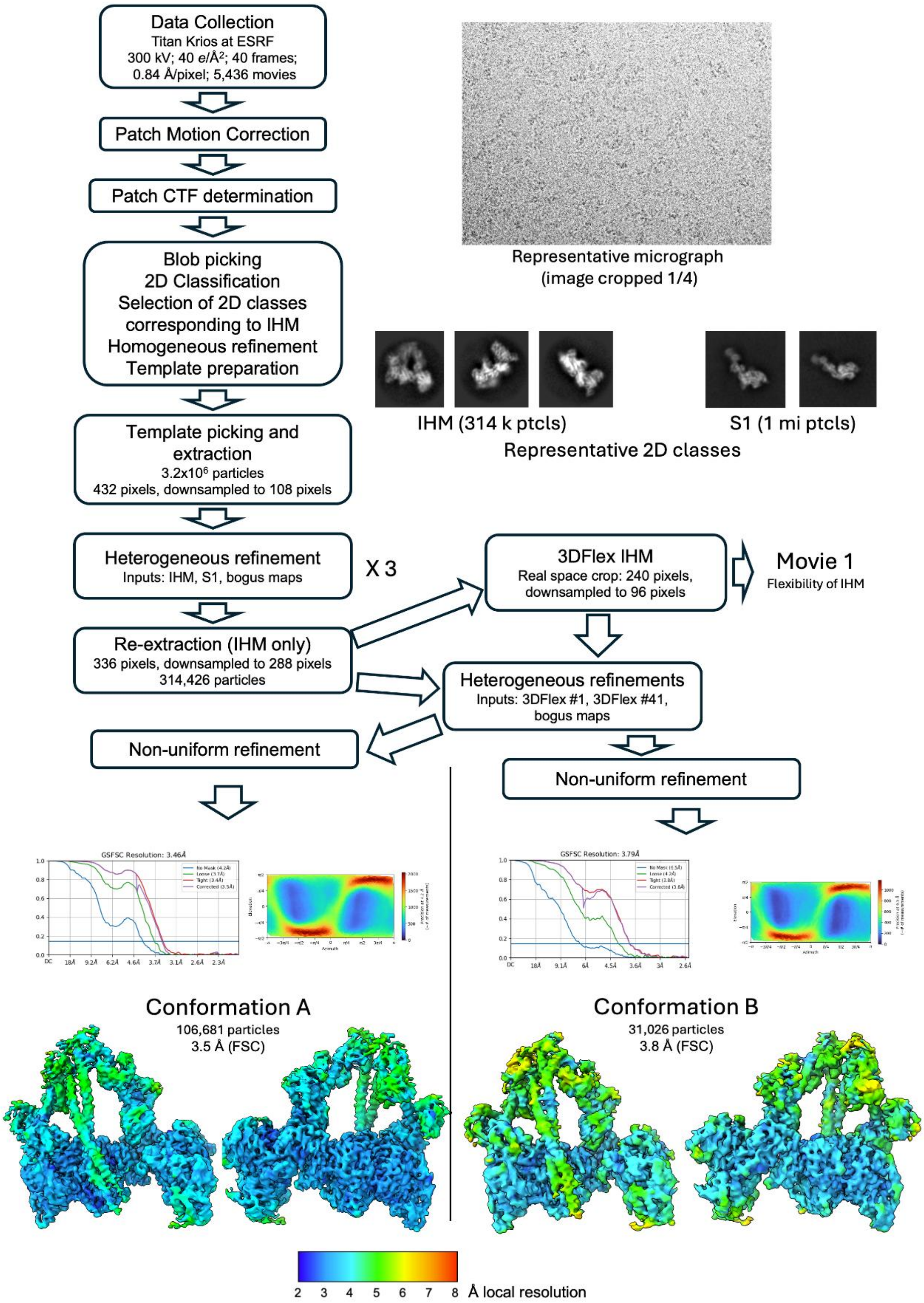

Supplementary Figure 2 – Data processing workflow for the WT $_{Car}$IHM from which conformations A and B were obtained (see Methods). All steps were performed with CryoSPARC v4.2.0+230302. ^{WT}ConfA and ^{WT}ConfB are two distinct conformations, but several other classes of intermediate conformations can be explored. The refined atomic models of ^{WT}ConfA and ^{WT}ConfB, superimposed on their respective final CryoEM map, are shown. Mi: million, ptcls: particles, FSC: Fourier shell correlation.

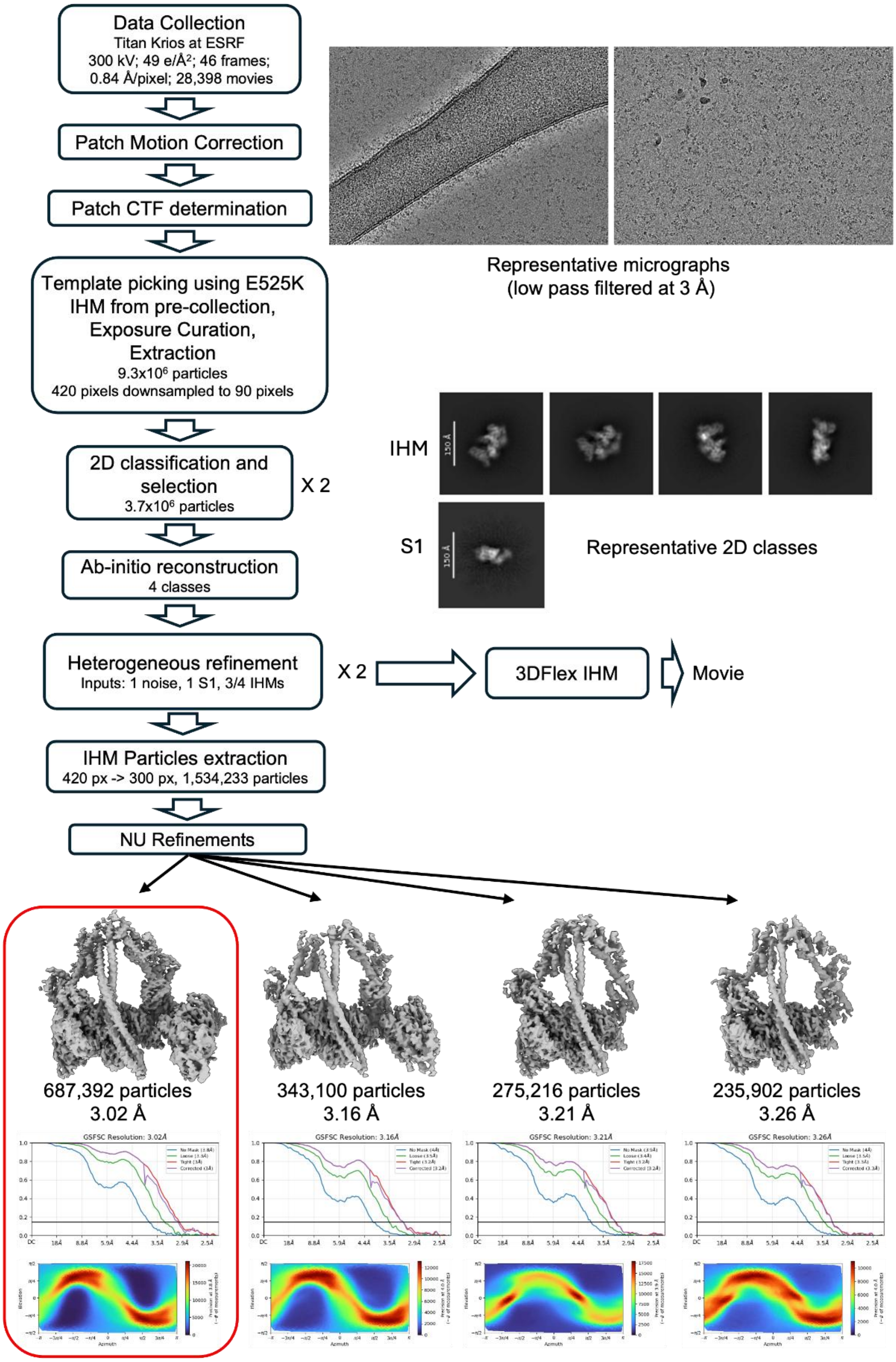

Supplementary Figure 3 – Processing of the CryoEM E525K-$_{Car}$IHM dataset provided distinct structures. All particles indicated a similar position for S2. In contrast, the position of the FH varies among particles, as indicated by the comparison of the first two structures and the fact that the FH density was not indicated for some potential maps. However, it is interesting to note that density was present for the Hook region, even when the FH density was absent. This suggests that the orientation of the FH is the most variable, while the BH/S2 and the RLC/RLC interface are more stable.

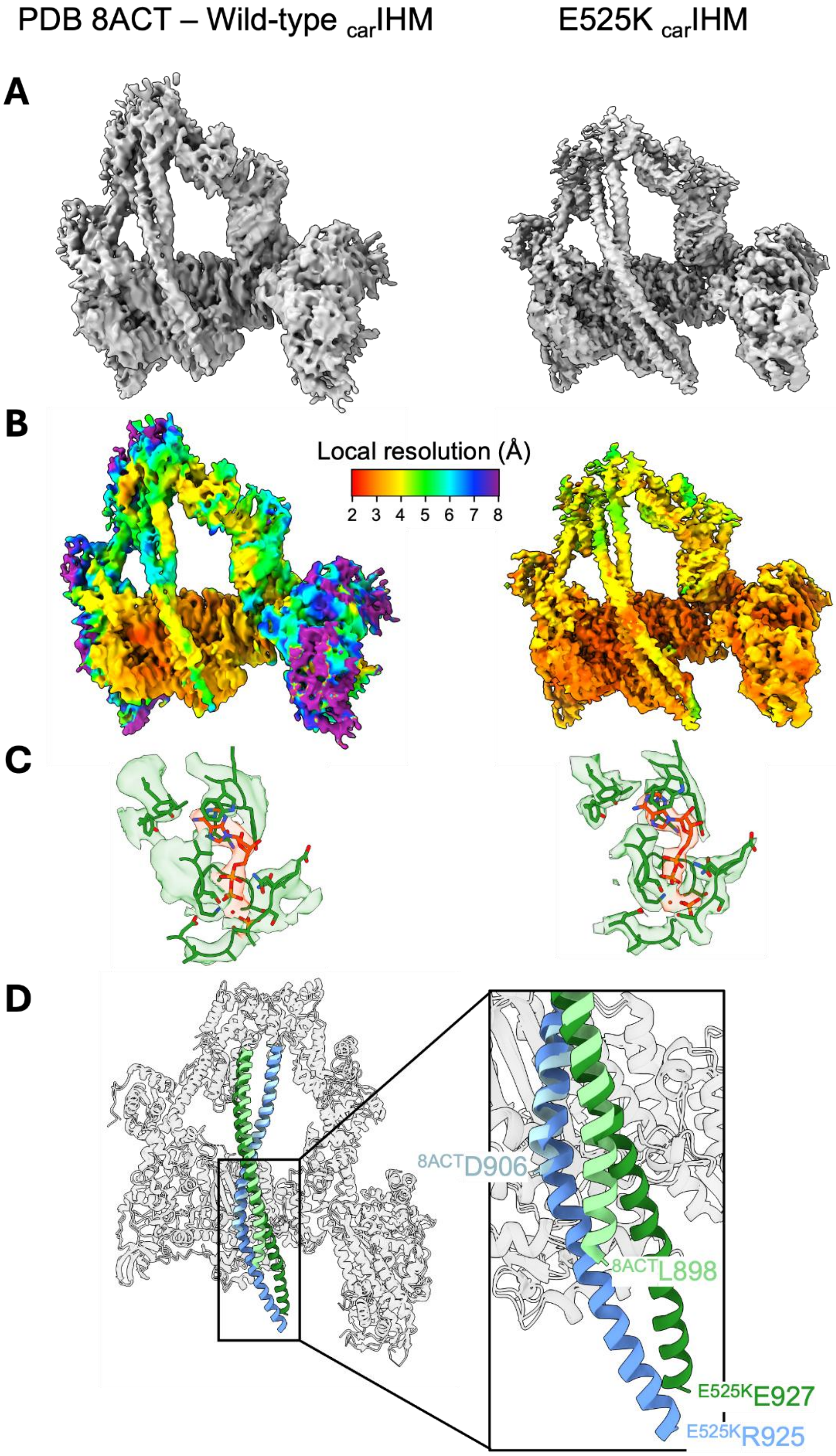

Supplementary Figure 4 – Comparison of the CryoEM map of the published wild-type $_{Car}$IHM structure PDB code 8ACT[1] with the CryoEM map of the $_{car}$IHM E525K mutant. (A) CryoEM maps. **(B)** CryoEM maps colored according to their local resolution. **(C)** Map-model overlays in the catalytic site of the BH. **(D)** Overlay of the atomic model of 8ACT and E525K.

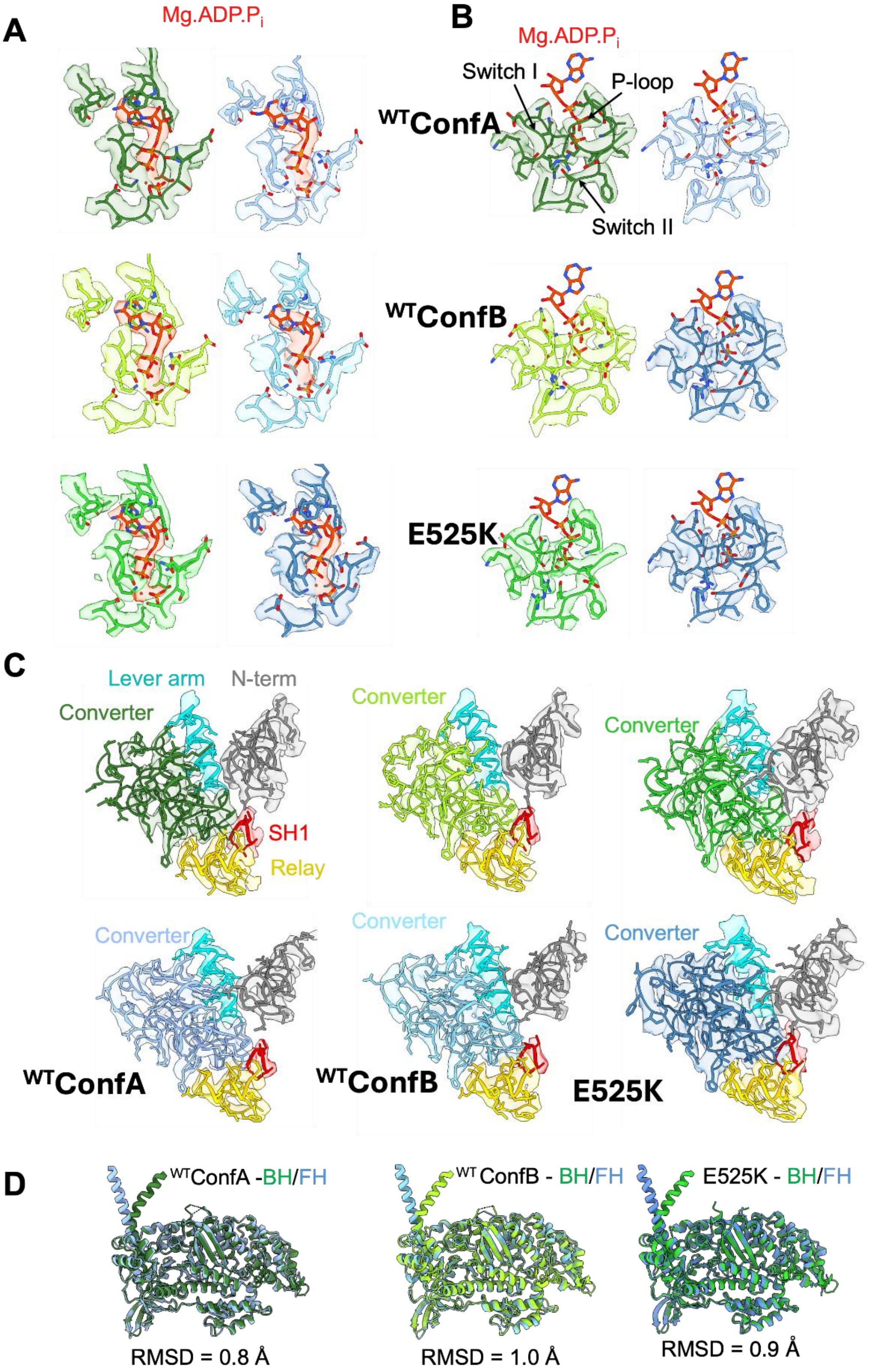

Supplementary Figure 5 – High-resolution features of the CryoEM maps of WT- and E525K-$_{Car}$IHM. **(A)** ATP hydrolysis products are present in the catalytic site. **(B)** The P_i backdoor is closed. **(C)** The Converter and the lever arm are primed. **(D)** Superposition of the BH and FH motor domains, respectively ($^{ConfA}RMSD_{1-780}$ = 0.749 Å and $^{ConfB}RMSD_{1-780}$ = 0.994 Å; $^{E525K}RMSD_{1-780}$= 0.895 Å). **(A-B)** the cryoEM map is colored green for BH and blue for FH. N-term: N-terminal subdomain.

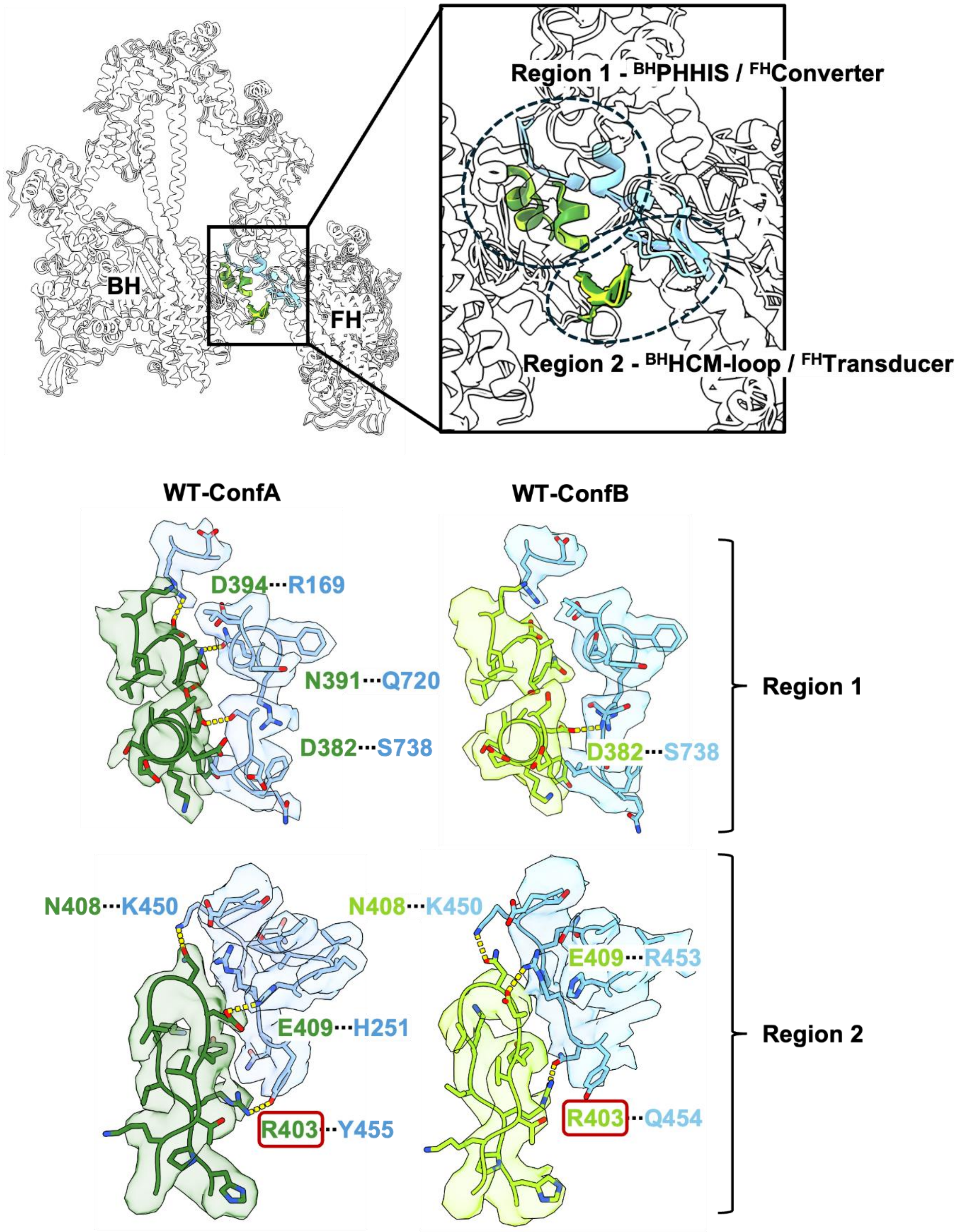

Supplementary Figure 6 – Differences at the BH/FH interface of the ^{WT}ConfA and ^{WT}ConfB $_{Car}$IHM structures. Bottom: Fit of the atomic models to CryoEM maps of two regions of the BH/FH interface. H-bonds between the BH and FH are shown as yellow dashed lines and the involved residues are indicated on the side. The complete list of BH/FH interactions is shown in Supplementary Table 3. The lack of interaction in ^{WT}ConfB in region 1 and the reshuffling of the H-bonding partners in region 2 explain the shift in the FH position of ^{WT}ConfB compared to ^{WT}ConfA. R403, a mutation hotspot in the HCM-loop, interacts differently in the two structures.

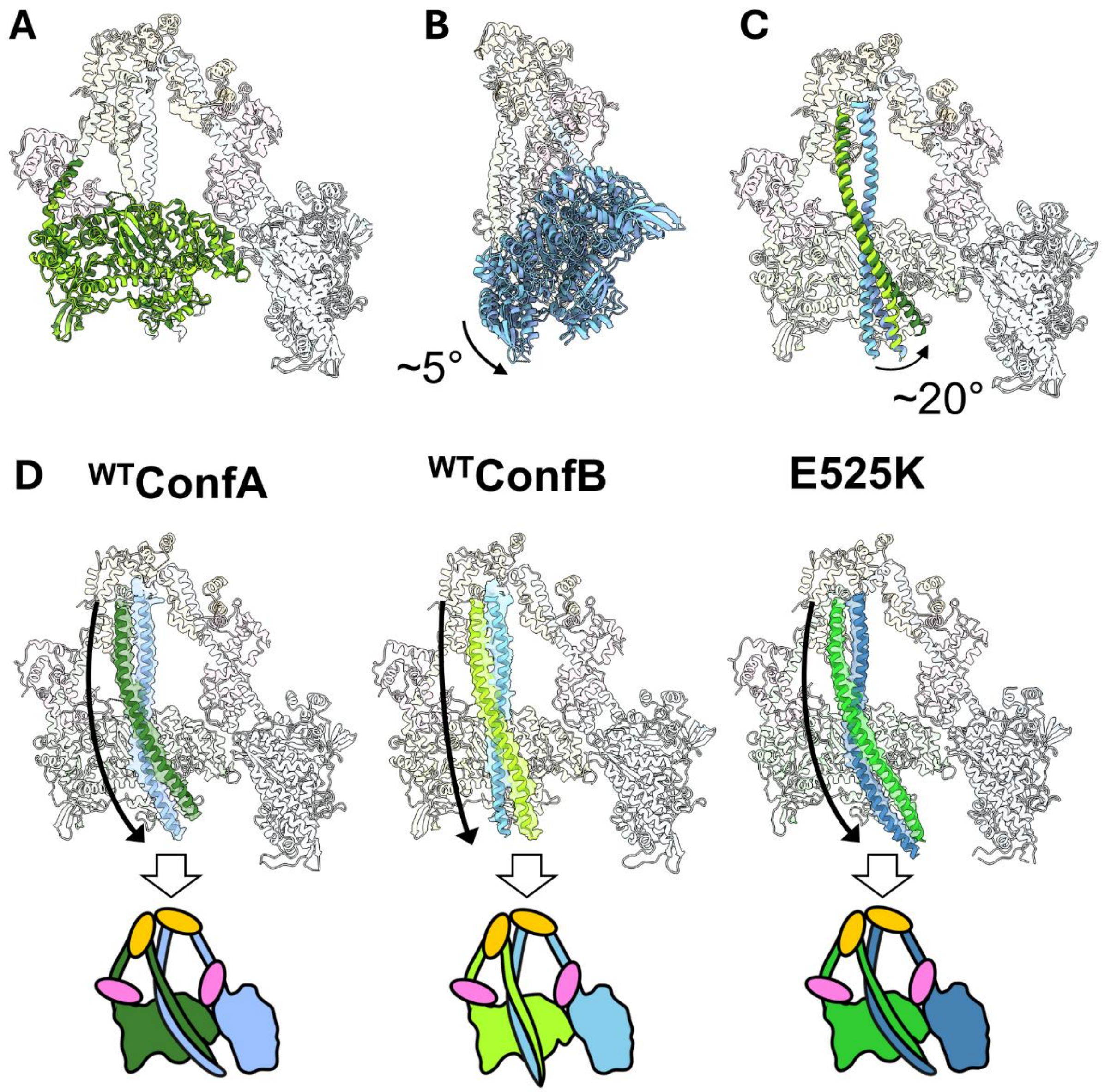

Supplementary Figure 7 – Comparison of two WT $_{Car}$IHM conformations ^{WT}ConfA and ^{WT}ConfB. (A) The BHs of the two conformations are very similar. **(B)** The FH changes its orientation relative to the rest of the structure by at least 5°, which is consistent with the plasticity of the network of interactions found at the BH/FH interface (**Supplementary Fig. 6**, **Supplementary Movie 2A**). **(C)** The position of S2 on the BH differs by a tilt of 20° when ^{WT}ConfA and ^{WT}ConfB are compared. **(D)** Fit of the S2 coiled-coil in the CryoEM maps of ^{WT}ConfA, ^{WT}ConfB and E525K $_{Car}$IHM. The position of S2 in E525K resembles that of ^{WT}ConfA. **(A)-(C)** Superposition of the ^{WT}ConfA and ^{WT}ConfB $_{Car}$IHM aligned along the BH.

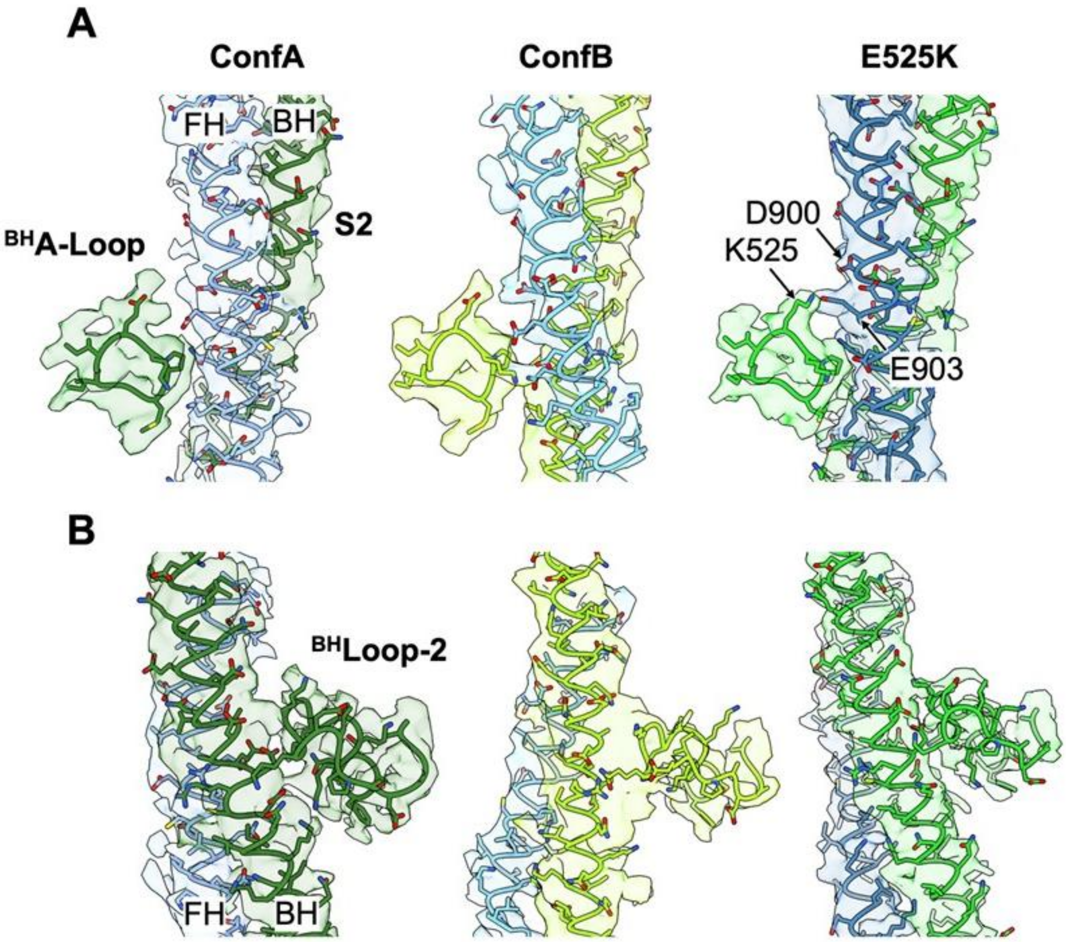

Supplementary Figure 8 – Distinct interactions of S2 with the ^{BH}Activation-loop (A-loop) and ^{BH}Loop-2 of $_{Car}$IHM ^{WT}ConfA, ^{WT}ConfB and E525K. (A) Interaction of the ^{BH}A-loop with S2 in the three structures. Each interface is defined in density. **(B)** Interaction of the ^{BH}Loop-2 with S2 in the three structures. The map density is weaker for the ^{BH}Loop-2, although it clearly interacts with the S2.

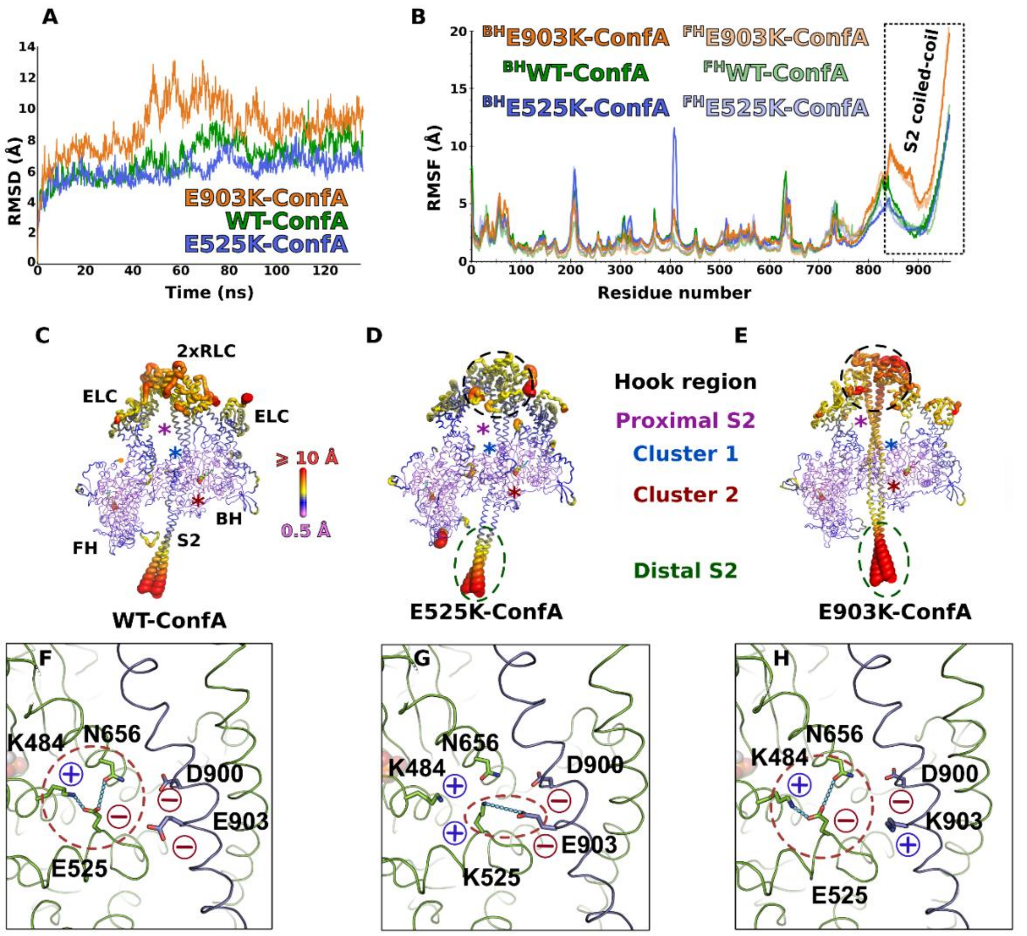

Supplementary Figure 9 – All atom molecular dynamics of WT, E525K and E903K ConfA $_{Car}$IHMs. (**A)** Root-mean square deviation (RMSD) plotted against time for the three all-atom MD simulations. **(B)** Shows the RMSF in the three conditions. The FH curve is shown with transparency and the position of the S2 coiled-coil is indicated. **(C, D)** and **(E)** display the putty representations of the root-mean square fluctuation (RMSF) for the entire simulation duration (135 ns). The regions of highest degree of flexibility are located in the Hook and lever arms as well as in the distal part of S2. Differences are found across simulations in the flexibility observed at the BH/S2 interface. **(D)** The introduction of the E525K mutation results in a significant decrease in S2 flexibility and, to a lesser extent, in the lever arm. **(E)** The introduction of the E903K mutation induces a drastic increase in S2 flexibility, especially in the proximal region, compared to ^{WT}ConfA **(C)**. The intrinsic flexibility of the lever arm and Hook region is observed in both experimental CryoEM and in silico simulations, confirming that our MD simulations can predict the location and magnitude of flexibility observed in the CryoEM data analyzed by 3DFlex (**Supplementary Movie 1**). Clusters 1 and 2 are shown with a blue and a dark red star, respectively. A purple star indicates the proximal S2 region in which Cluster 0 interactions stabilize the S2 bend for WT and E525K, but not E903K. Note the difference in dynamics in this region for the E903K simulation. **(F, G)** and **(H)** illustrate the consequences of the mutations of E525K or E903K to explain why they lead to distinct stability for the $_{Car}$IHM motif. **(F)** In the WT, E525 interacts with K484 and N656, thus being stabilized. The coiled-coiled is not fully stabilized and only vdW interactions can form. **(G)** The introduction of K525 changes the network of interactions, polar bonds can form with two negatively charged residues: E903 or D900 (see **Supplementary Movie 3**), thus stabilizing the position of the S2 in an orientation close to ConfA. **(H)** When K903 is introduced, a positive charge is found on the coiled-coil. However, on the BH surface, E525 is involved in interactions with K484 and N656. Thus, no polar bond is formed between the BH and S2 coiled-coil. Contrary to E525K, E903K thus induces a destabilization of the S2 because it has no negative charge to interact with. See **Supplementary Data 1** for raw data.

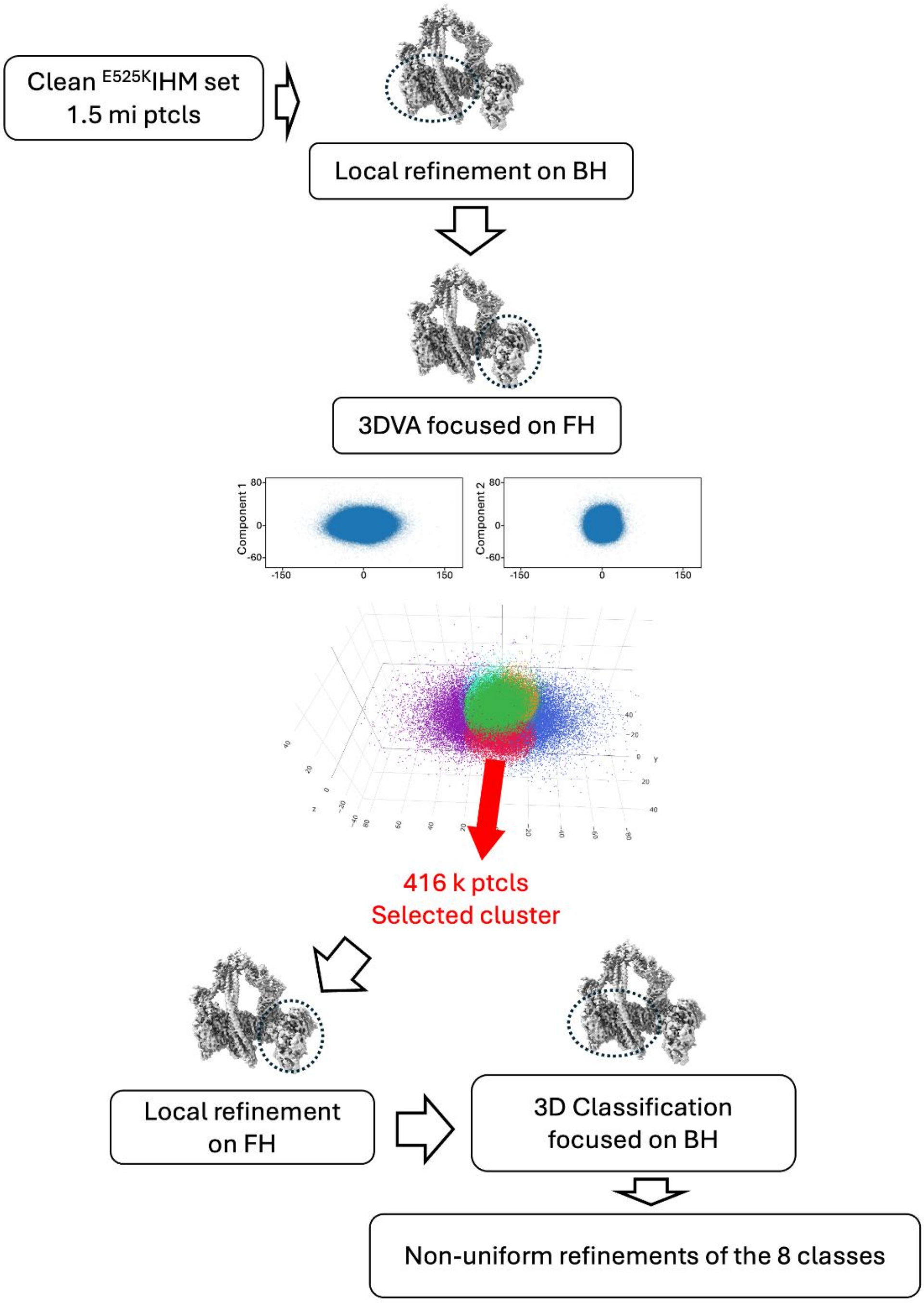

Supplementary Figure 10 – The workflow used for the identification of the dynamic Cluster 3 of $^{E525K}{}_{Car}$IHM. This workflow generated the maps shown in Figure 3. On the 3D Variability Analysis (3DVA) plots, the values correspond to the latent dimensions of each component. The blue dotted plots are cross-sections of the 3D plot below, showing the clusters in colors. The red cluster was chosen for further classifications. See **Methods** for a detailed explanation. Mi: million, 3DVA: 3D variability analysis.

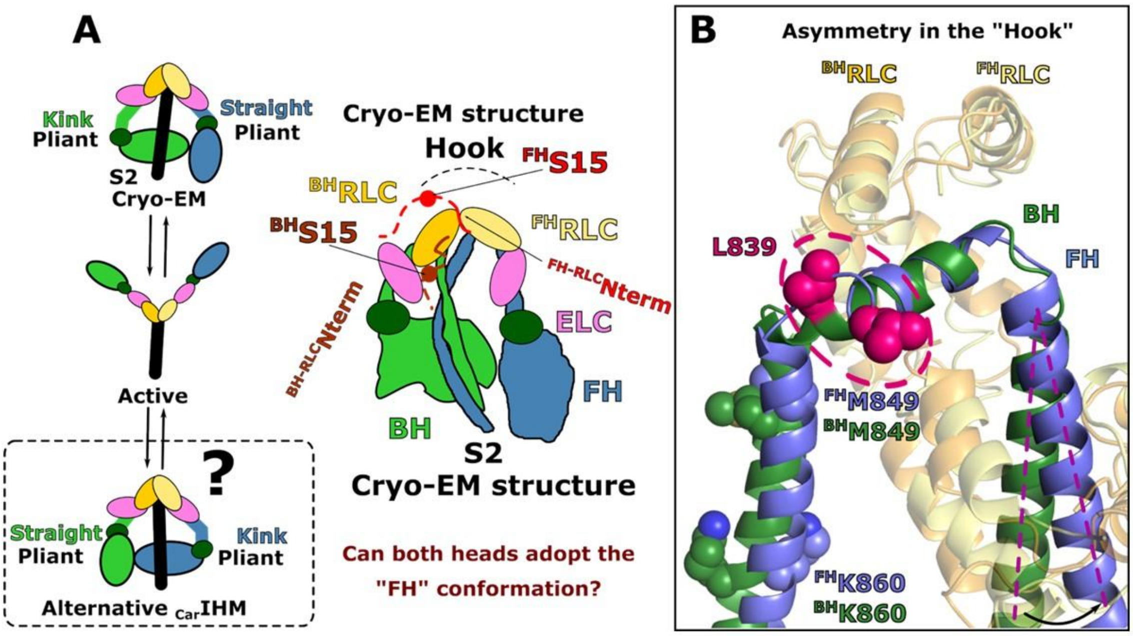

Supplementary Figure 11 – The asymmetry of $_{Car}$IHM. (A) Left: Two cardiac myosin heads are assembled via the S2 coiled-coil. The drawings illustrate two hypothetical $_{Car}$IHM configurations that would be expected if either head could act as a BH. Note that the $_{Car}$IHM surface visible in this view, with S2 in front of the BH, is the surface that would be associated with the thick filament surface. Importantly, it is asymmetric and distinct in the top and bottom drawings. Thus, only one of these configurations can dock with the thick filament surface. **Right:** Structural studies indicate that there is only one $_{Car}$IHM structure. This means that only one head can act as a BH. The two RLCs establish different stabilizing interactions with S2. The last residues of the N-terminal extension of the ^{FH}RLC ($^{FH-RLC}$NTE, aa 1-20) are oriented close to the RLC/RLC interface, although the N-terminal region of this extension is disordered, hence the $^{BH-RLC}$NTE is not part of the RLC/RLC interface. For both RLCs, CryoEM density indicates that the phosphorylatable S15 (red sphere) is not directly involved in strong stabilizing interactions. **(B)** Superimposition of the Hook region of the two heads (BH and FH) using the Cα of the N-terminal lobe of the RLC. A slight asymmetry in the helices of the coiled-coil is observed. It begins at residue L839 (circled in hot pink) and is maintained throughout the S2 helix. The positions of M849 and K860 are shown as spheres to illustrate this asymmetry. A difference in the orientation of the lever arm occurs, as shown with deep purple dashed lines with an arrow. Altogether, these differences occur upon the formation of the RLC/RLC interface and result in the asymmetry of $_{Car}$IHM.

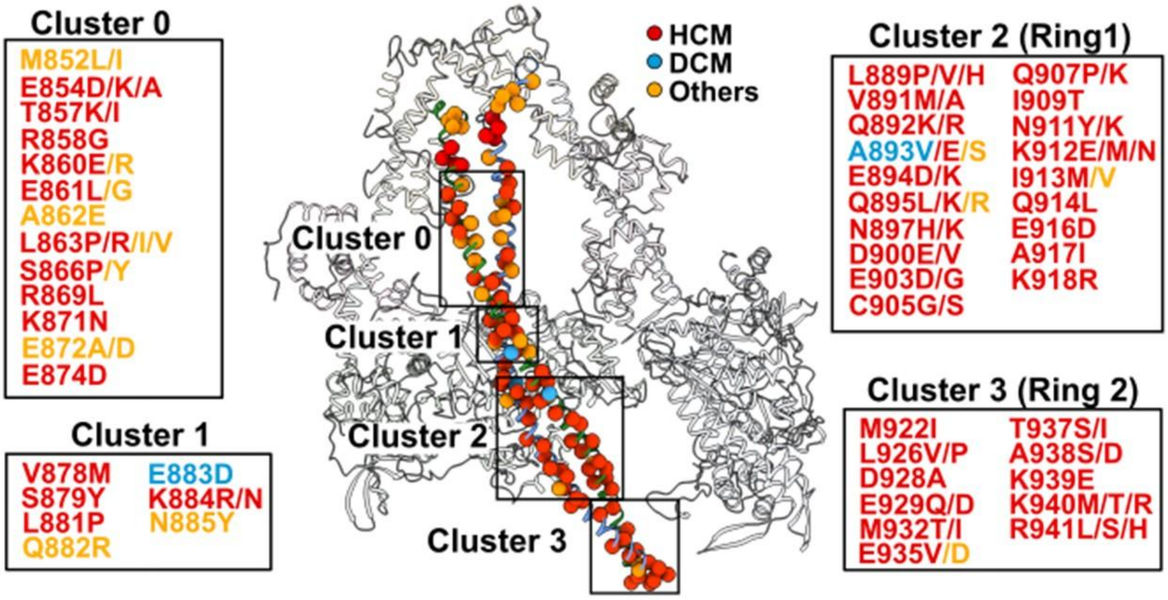

Supplementary Figure 12 – Variants of Unknown Significance (VUS) in the S2 region according to ClinVar. Each variant is annotated based on its reported association with hypertrophic or dilated cardiomyopathy (HCM in red; DCM in blue). VUS for uncharacterized cardiomyopathies or other muscle disorders are reported in orange.

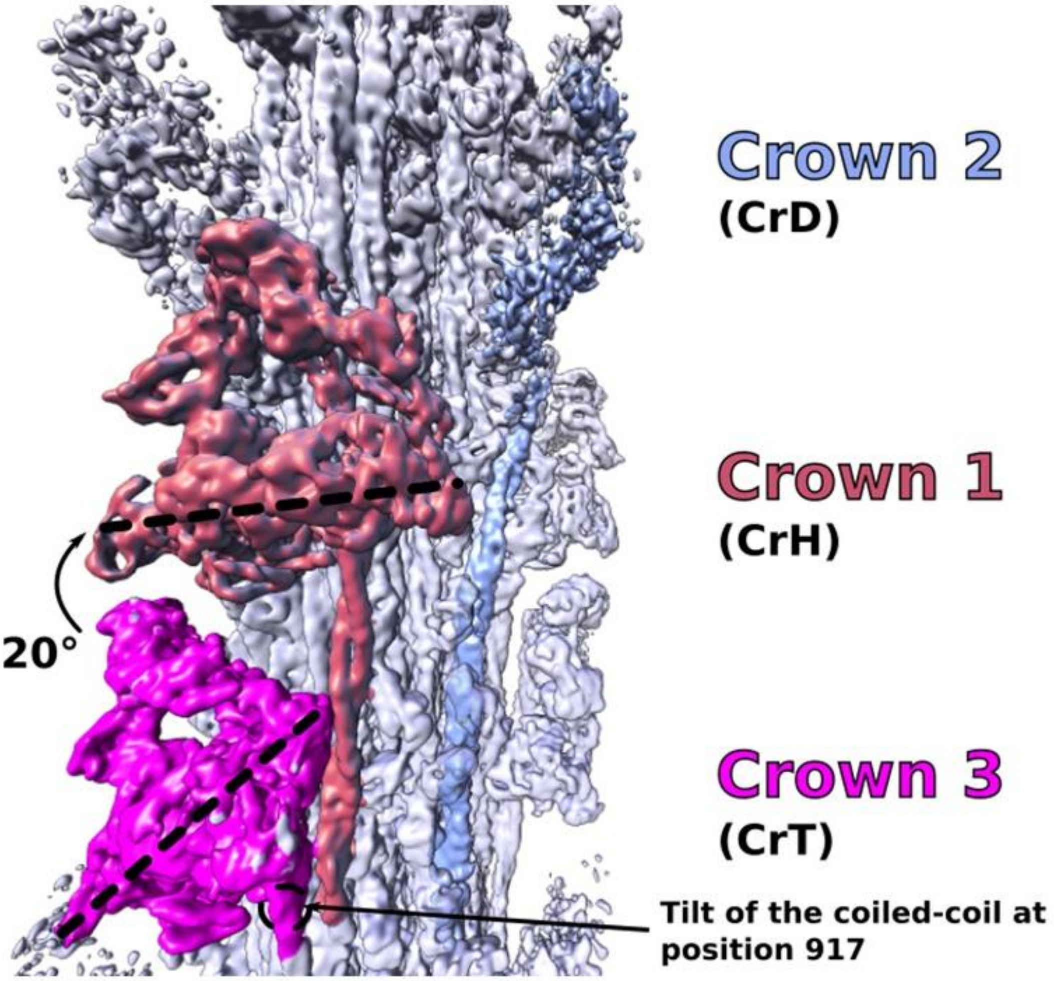

Supplementary Figure 13 – Three-fold pseudo-symmetry of the relaxed cardiac filament, illustrated by the 6 Å resolution map (EMD-29722[6]). Three crowns can be identified: a disordered **Crown 2** whose density appears fragmented (Cr2, or CrD), a horizontal **Crown 1** (Cr1, or CrH) and a tilted **Crown 3** (Cr3, or CrT). Cr3 is tilted by approximately 20° relative to Cr1 due to a bend in the S2 coiled-coil at the position 917. The map is contoured at 0.118. CrT: tilted crown, CrH: horizontal crown, CrD: disordered crown. CrT, CrH, CrD is the nomenclature used in[6].

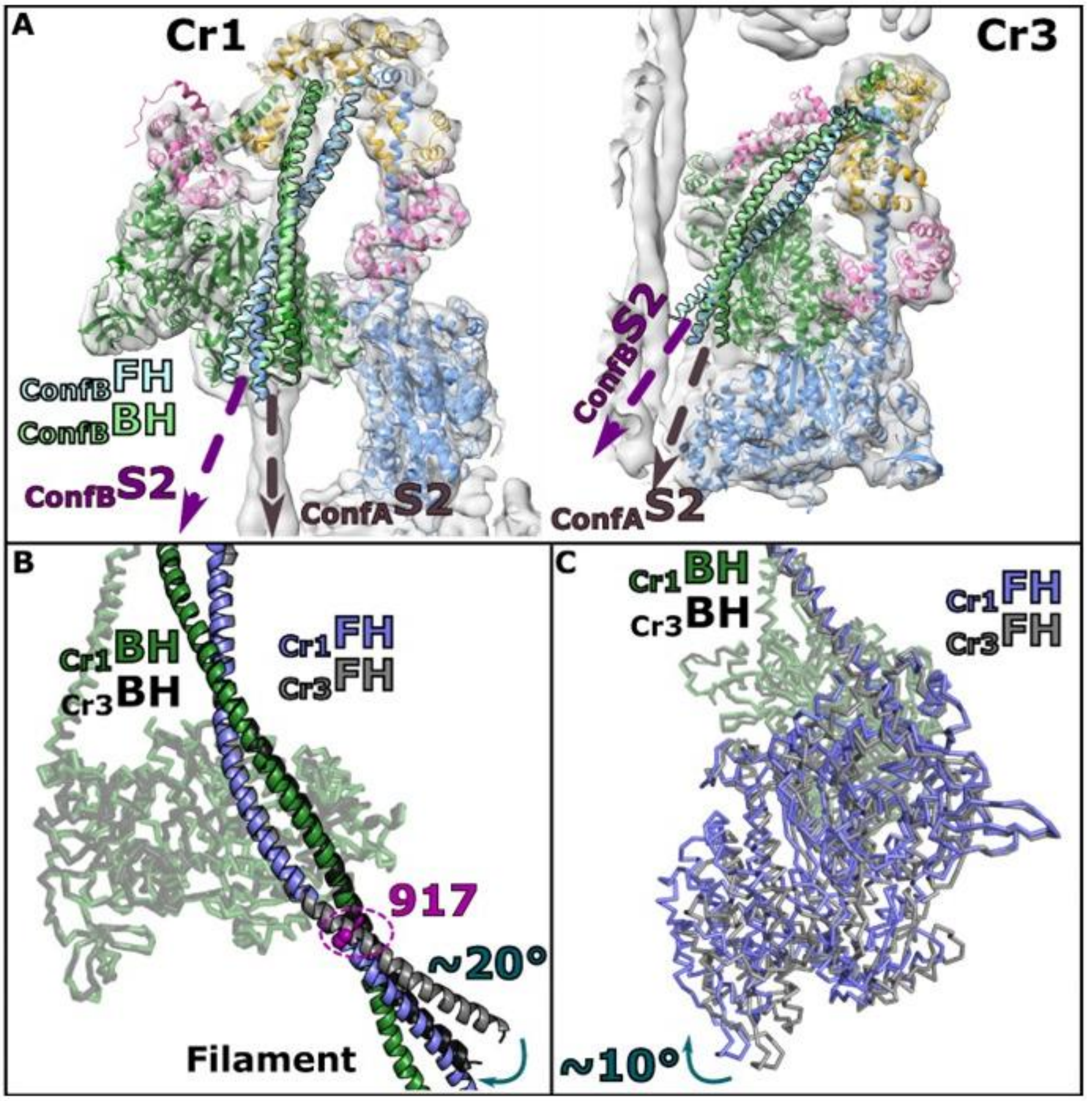

Supplementary Figure 14 – Comparison of Crowns 1 and 3. **(A)** Density of Crown 1 (Cr1) and Crown 3 (Cr3) from EMD-29734[6] with rigid body fitting of $_{Car}$IHM structures ^{WT}ConfA and ^{WT}ConfB. A good fit is obtained with ^{WT}ConfA in both crowns. When ^{WT}ConfB is positioned, the S2 coiled-coil deviates from the density observed at the BH interface due to the differences observed on the interactions of Cluster 2 (linked to a kink observed at D896, see Fig. 1B, 1C). **(B)** and **(C)** Superimposition of Cr1 and Cr3. The ELCs are colored in pink and the RLCs in yellow. **(B)** The orientation of the coiled-coils of Cr1 and Cr3 deviates by ~20° downstream of Cluster 2, at position 917. **(C)** The relative angle between the BH and the FH varies by ~10° between Cr1 and Cr3. In all panels, the structures are superimposed on the BH motor domain (residues 1-710). The light chains are omitted in B and C for clarity.

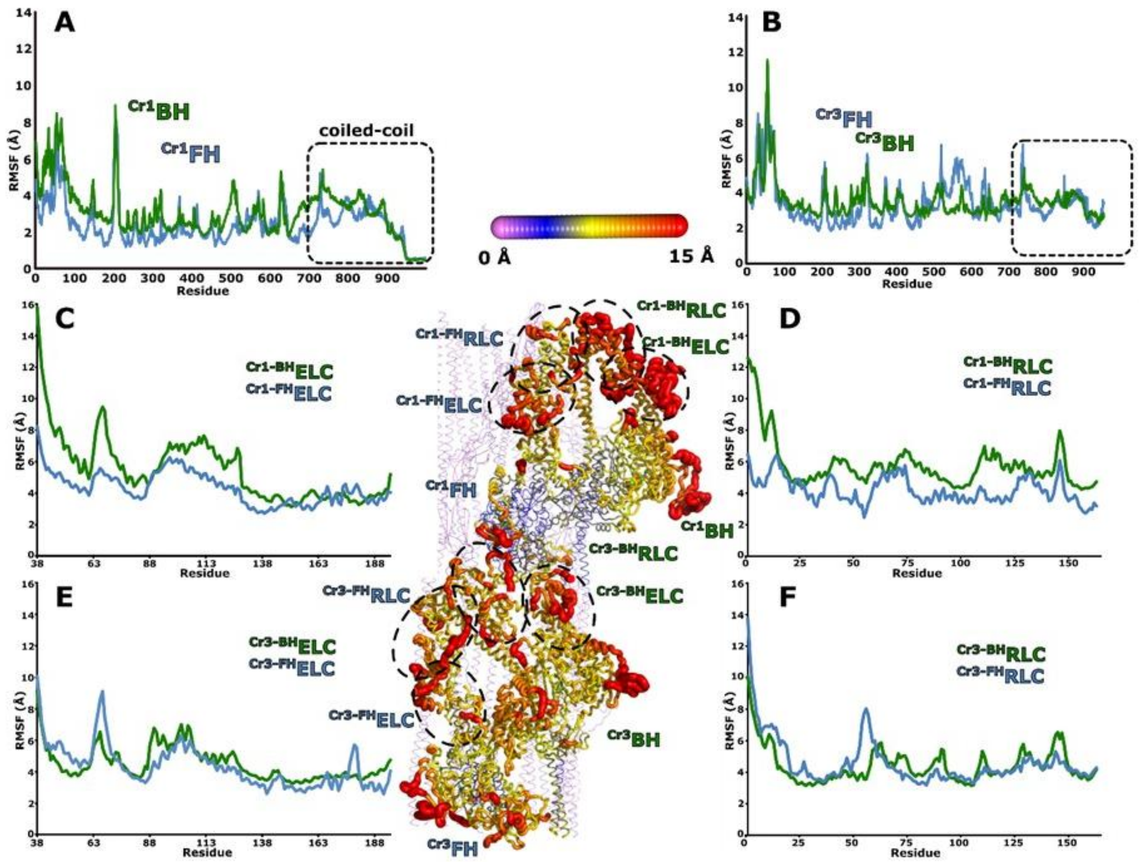

Supplementary Figure 15 – Dynamics of the Crown 1 (Cr1) and Crown 3 (Cr3) light chains within the human cardiac relaxed filament. **(A)** and **(B)** show the root mean square fluctuations (RMSF) of the blocked head (BH) and the free head (FH) of each crown (Cr1 and Cr3) during the time course of the simulation (in Å). **(C)** and **(D)** show the RMSF curves of ELCs and RLCs of the BH and the FH from Crown 1: $^{Cr1\text{-}BH}$ELC, $^{Cr1\text{-}FH}$ELC, $^{Cr1\text{-}BH}$RLC, $^{Cr1\text{-}FH}$RLC. **(E)** and **(F)** show the RMSF curves of the BH and the FH from Crown 3: $^{Cr3\text{-}BH}$ELC, $^{Cr3\text{-}FH}$ELC, $^{Cr3\text{-}BH}$RLC, $^{Cr3\text{-}FH}$RLC. In the center, a putty representation of the RMSF of the all-atom molecular dynamics of the relaxed human cardiac filament is displayed. See **Supplementary Data 2 and 3** for raw data.

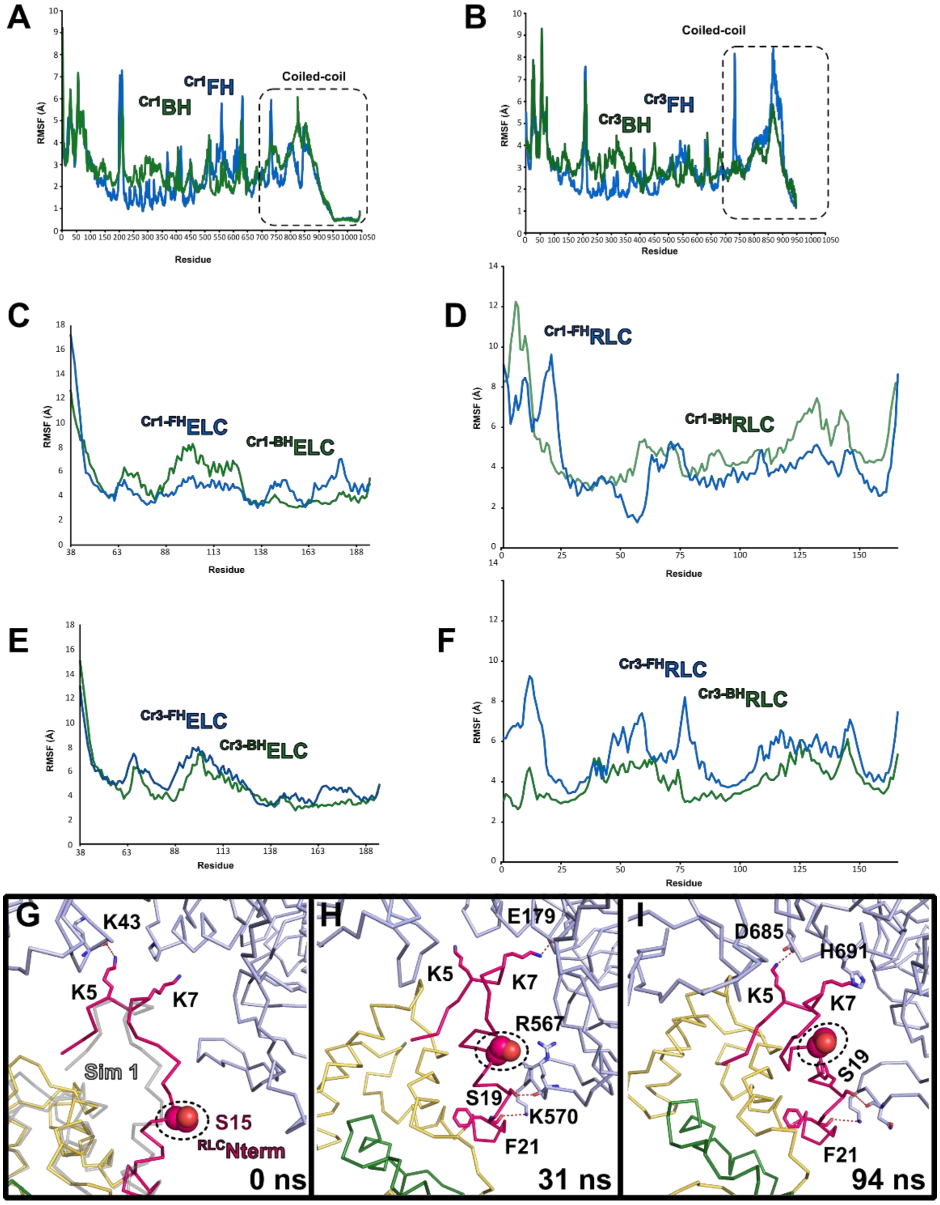

Supplementary Figure 16 – Molecular dynamics simulations of human relaxed cardiac filament are reproducible. RMSF curves for the BHs and the FHs (**A**, **B**), the ELCs (**C**, **E**) and the RLCs (**D**, **F**) of Crown 1 (Cr1) and 3 (Cr3) are shown for a replicate experiment of the dynamics of the filament (see Supplementary Fig. 15). As in the first experiment, the FH is more stabilized compared to the BH in the two heads and the same patterns are observed, leading to the same conclusions. (**G, H, I**) Three representative frames showing the N-terminal extension of the ^{BH}RLC from Crown 3 (Cr3) (^{RLC}Nterm) during the duplicate molecular dynamics simulation. Three time points (0, 31, and 94 ns) are shown to illustrate that, despite being built differently compared to the first simulation (Sim 1, shown in gray in **G**), ^{RLC}Nterm establishes contacts with similar regions at the intercrown interface during the simulation (see Fig. 7A). The same conclusions can therefore be drawn from both thick filament dynamic simulations. See Supplementary Data 2, 3 and 4 for raw data.

Supplementary Tables

Supplementary Table 1 – CryoEM collection parameters and model building and refinement statistics.

	E525K	WT-ConfA	WT-ConfB
Microscope	Titan KRIOS G3	Titan KRIOS G3	
Voltage (kV)	300	300	
Detector	K3	K3	
Magnification (kx)	105	105	
Total electron exposure (e^-/A^2)	49	39.2	
Defocus range (um)	-0.8, -2.2	-0.8, -2.2	
Pixel size (Å)	0.84	0.84	
Initial number of particles	3.7 million	3.2 million	
Final particle number	687,392	106,681	31,026
Symmetry imposed	C1	C1	C1
Map resolution (Å) at FSC threshold 0.143	3.0	3.5	3.8
Map local resolution range (Å) at FSC threshold 0.143	2.6 – 5.5		
EMD-ID	TBD	TBD	TBD
Initial model used (#PDB)	8ACT	8ACT	8ACT
Model resolution (Å) at FSC threshold 0.5 (masked)	3.4	3.7	4.1
# of non-H atoms	19336	19146	19191
# of protein residues	2386	2376	2369
# of ligands	6	6	6
# of waters	0	0	0
Bond length RMSD (Å)	0.004	0.004	0.003
Bond angles RMSD (deg)	0.692	0.646	0.631
Molprobity score	1.87	1.92	2.01
Clash score	13.11	12.70	15.01
Rotamer outliers (%)	0.05	0	0.05
Ramachandran (%)			
- Outliers	0	0.04	0
- Allowed	3.62	4.32	4.68
- Favored	96.38	95.63	95.32
CC (mask)	0.74	0.77	79
CC (volume)	0.72	0.77	78
#PDB	TBD	TBD	TBD
RMSD stands for root mean square deviation.			

Supplementary Table 2 – Matrix comparing the root mean square deviations of different heads aligned on the motor domain (residue 1-780). It includes the comparison of myosin heads in ConfA, ConfB and E525K, using Cα of the motor domain (residues 1-780). For each model, the heads are compared with a classical PPS conformation (PDB code 8QYP[8]). It demonstrates that both the BH and the FH are similar in each structure but also that in all the models both the BH and the FH are similar to the canonical PPS conformation.

Model 1	Model 2	$RMSD_{1\text{-}780}$ (Å)
WT ConfA:		
8QYP	ConfA-BH	0.693
8QYP	ConfA-FH	0.839
ConfA-BH	ConfA-FH	0.749
WT ConfB:		
8QYP	ConfB-BH	0.845
8QYP	ConfB-FH	0.994
ConfB-BH	ConfB-FH	0.994
E525K:		
8QYP	E525K-BH	0.782
8QYP	E525K-FH	0.894
E525K-BH	E525K-FH	0.895

Supplementary Table 3 – Interactions stabilizing cardiac IHM

Differences in the existence or type of interaction between the two WT conformations are outlined with a light salmon background. When distinguishable, atom names are given according to the PDB convention. Abbreviations: 'sc': side chain; 'mc': main chain; 'CO': carbonyl; 'vdW': van der Waals. **Cardiomyopathy-causing mutations** predicted to destabilize the interfaces are indicated. The number of stars close to each mutation corresponds to the ClinVar annotation: "no classification or no assertion criteria provided" (0 stars); "criteria provided, single submitter" (1 star); "criteria provided, multiple submitters" (2 stars); "reviewed by expert panel" (3 stars); and "practice guideline" (4 stars); HCM ❷; DCM ❸; other CM ❹. The Pathological and likely pathological variants ("Patho/LP"), as well as the Variants of Unknown Significance (VUS) according to Clinvar are indicated.

(i) Interactions between the Motor domains

BH HCM-loop / FH Transducer (HO-linker and β-bulge)				
**BH**	**FH**	**Conformation A**	**Conformation B**	**Patho/LP variants**
$_{\text{HCM-loop}}$R403	$_{\text{HO-linker}}$Q454<br>$_{\text{HO-linker}}$Y455	sc – NE2 (Polar)<br>NH2 – OH (**H-Bond**)	sc – sc (**H-Bond**)<br>sc – Phenyl (stacking)	R403W/Q/G/L***❷<br>VUS: R403P**❹
$_{\text{HCM-loop}}$N408	$_{\text{HO-linker}}$E448<br>$_{\text{HO-linker}}$K450<br>$_{\text{HO-linker}}$R453<br>$_{\text{HO-linker}}$R453	OD1 – OE2<br>OD1 – NZ (**H-Bond**)<br>sc – sc (vdW)<br>mc – mc	ND2 – OE2<br>OD1 – NZ (**H-Bond**)<br>sc – sc (Polar)<br>mc – mc	K450N**❷<br>R453C/S/H/L***❷<br>VUS: N408K**❷<br>K450R/E**❷
$_{\text{HCM-loop}}$E409	$_{\beta\text{-bulge}}$R249<br>$_{\beta\text{-bulge}}$H251<br>$_{\text{HO-linker}}$R453	OE1 – NH2 (**H-Bond**)<br>OE1.OE2 – NE2 (**H-Bond**)<br>No interaction	No interaction<br>No interaction<br>OE1 – NH1 (**H-Bond**)	R249P/L/Q**❷<br>R453C/S/H/L***❷❹<br>VUS: H251Y*❷<br>E409G*❷
$_{\text{HCM-loop}}$Y410	$_{\text{HO-linker}}$Q454	Phenyl - sc	Phenyl - sc	None<br>VUS: Y410H*❷

BH Loop4 / FH Relay				
**BH**	**FH**	**Conformation A**	**Conformation B**	**Patho/LP variants**
$_{\text{Loop4}}$K367<br>$_{\text{Loop4}}$Q368<br>$_{\text{Loop4}}$E374	$_{\text{Relay}}$K503	sc – NZ (vdW)<br>No interaction<br>OE1 – NZ (**H-Bond**)	No interaction<br>OE1 – NZ (**H-Bond**)<br>sc – NZ (Polar)	K367N❹<br>VUS: Q368P*❷<br>E374K/A/V*❷

BH PHHIS / FH Converter				
**BH**	**FH**	**Conformation A**	**Conformation B**	**Patho/LP variants**
		**Intramolecular BH**		
$_{\text{HJ-helix}}$L302<br>$_{\text{HM-helix}}$D382<br>$_{\text{HM-helix}}$K383<br>$_{\text{HM-helix}}$Y386<br>$_{\text{HN-helix}}$S392	-	L302-sc – K383-sc<br>L302-sc – Y386-sc<br>D382-OD1 – S392-OG (**H-Bond**)	L302-sc – K383-sc (closer)<br>L302-sc – Y386-sc (farther)<br>D382 – Y386 vdW	L302Q* ❹<br>D382N/G*❷<br>Y386C***❹<br>S392L❸<br>VUS: D382H/A*❷❹<br>K383R***❷<br>S392T*❷
		**BH – FH interactions**		**Patho/LP variants**
$_{\text{HJ-helix}}$L302	$_{\text{T-loop}}$I736	sc – sc (vdW)	sc – sc (vdW)	L302Q* ❹<br>I736T***❷<br>VUS: I736F*❹
$_{\text{HM-helix}}$T378	$_{\text{Conv}}$S738	OG1 – OG (**H-Bond**)	No interaction	S738R❷<br>VUS: S738T/G/N***❷❹
$_{\text{HM-helix}}$E379	$_{\text{T-loop}}$D737<br>$_{\text{T-loop}}$S738	OE1 – OD1<br>OE1 – OG	No interaction<br>No interaction	S738R❷<br>VUS: E379*❷<br>D737Y***❷<br>S738T/G/N***❷❹

$_{\text{HM-helix}}$D382	$_{\text{T-loop}}$I736<br>$_{\text{T-loop}}$S738	sc – sc (vdW)<br>OD2 – OG (**H-Bond**)	sc – sc (vdW)<br>OD2 – N (**H-Bond**)	D382N/G*❷<br>I736T***❷<br>S738R❷<br>VUS: D382H/A*❷❹<br>I736F*❹<br>S738T/G/N***❷❹
$_{\text{HM-helix}}$K383	$_{\text{T-loop}}$I736	No interaction	NZ – CO (Polar)	I736T***❷<br>VUS: K383R***❷<br>I736F*❹
$_{\text{HM-helix}}$Y386	$_{\text{T-loop}}$G733<br>$_{\text{T-loop}}$I736	OH – mc (Polar)<br>sc – sc (vdW)	OH – CO (**H-Bond**)<br>sc – sc (vdW)	Y386C***❹<br>G733V/E**❷<br>I736T***❷<br>VUS: G733R*❹<br>I736F*❹
$_{\text{HN-helix}}$N391<br>$_{\text{HN-helix}}$S392	$_{\text{Conv}}$Q720<br>$_{\text{Conv}}$G716	ND2 – OE1 (**H-Bond**)<br>OG – mc (Polar)	No interaction<br>No interaction	S392L❸<br>G716R***❷<br>VUS: N391S**❷<br>S392T*❷
$_{\text{HN-helix}}$A393	$_{\text{Conv}}$L714<br>$_{\text{Conv}}$G716<br>$_{\text{Conv}}$D717	CB – sc (vdW)<br>CB – mc (vdW)<br>CB – sc (vdW)	No interaction<br>CB – mc (vdW)<br>CB – sc (vdW)	G716R***❷<br>D717V/G/A*❷❹<br>VUS: A393T/D**❷<br>L714P/F/I**❹<br>D717E*❷
**BH PHHIS / FH HD-linker and ELC**				**Patho/LP variants**
$_{\text{HN-helix}}$K397	$_{\text{HD-linker}}$D168<br>$_{\text{HD-linker}}$E170	NZ – CO (**H-Bond**)<br>No interaction	No interaction<br>NZ – OE2 (Polar)	None<br>VUS: D168N**❷<br>E170G*❹<br>K397N**❷
$_{\text{HN-helix}}$D394	$_{\text{HD-linker}}$R169<br>$_{\text{Conv}}$Q720	OD1 – NH2 (**H-Bond**)<br>OD2 – NE2 (Polar)	No interaction<br>No interaction	R169S/G*❷<br>D394E**❷<br>VUS: None
$_{\text{HU-helix}}$ K611	$_{\text{ELC-loop3}}$E143-N145	sc – mc (vdW)	sc – mc (vdW)	R143Q/W/G***❷<br>K611N*❷<br>VUS: K145E**❷

(ii) Proximal S2 (Cluster 0)

Non-canonical intra-coiled-coil Interactions within the proximal region of S2 that contribute to stabilize the bend required to form the IHM, observed in the $_{\text{Car}}$IHM WT-ConfA, WT-ConfB and E525K CryoEM structures as shown in **Fig. 4B**.

Residue in BH	Residue in FH			Patho/LP variants
	^{WT}**Conformation A**	^{WT}**Conformation B**	**E525K**	
^{BH}M849	^{FH}M849 (vdW)<br>^{FH} M852 (vdW)	^{FH}E848 (vdW)<br>^{FH}M849 (vdW)<br>^{FH}M852 (vdW)	No interaction	E848G** ❷<br>M849T ❷<br>M852K/T**❷<br>VUS:<br>M852L/V*❹
^{BH} K853	^{FH} M852 (vdW)<br>^{FH}E855 (Polar)<br>^{FH} F856 (vdW)	^{FH}E848 (Polar)<br>^{FH}M852 (vdW)	^{FH}E848 (Polar)<br>^{FH}M852 (vdW)	E848G** ❷<br>M852K/T**❷<br>L859P*❷<br>VUS:<br>M852L/V*❹<br>F856L*❹<br>L859F*❹
^{BH}F856	^{FH}E855 (vdW)<br>^{FH}F856 (vdW)	^{FH}M852 (vdW)<br>^{FH}E855 (vdW)	^{FH}E855 (vdW)<br>^{FH}F856 (vdW)	M852K/T**❷<br>L859P*❷

	^{FH}L859 (vdW)	^{FH}F856 (vdW)<br>^{FH}L859 (vdW)	^{FH}L859 (vdW)	VUS: M852L/V*❹<br>F856L*❹<br>L859F*❹
^{BH}K860	^{FH}E855 (Polar)<br>^{FH}L859 (vdW)	^{FH}E855 (Polar)<br>^{FH}L859 (vdW)	^{FH}L859 (vdW)	L859P*❷<br>VUS: L859F*❹<br>K860E/R**❷❹
^{BH}L863	^{FH}L859 (vdW)<br>^{FH}L863 (vdW)	^{FH}L859 (vdW)<br>^{FH}K860 (vdW)<br>^{FH}L863 (vdW)	^{FH}L859 (vdW)<br>^{FH}A862 (vdW)<br>^{FH}L863 (vdW)	L859P*❷<br>VUS: K860E/R**❷❹<br>A862V/E/G**❹<br>L863R/P*/I/V**❷❹
^{BH}E867	^{FH}A862 (vdW)<br>^{FH}S866 (Polar)	^{FH}A862 (vdW)<br>^{FH}K865 (vdW)	^{FH}K865 (H-bond)<br>^{FH}S866 (Polar)	K865E***❷<br>VUS: A862V/E/G**❹<br>S866P/Y**❷❹<br>E867K**❹
^{BH}R870	^{FH}S866 (vdW)<br>^{FH}R869 (vdW)<br>^{FH}R870 (vdW)	^{FH}S866 (vdW)<br>^{FH}R869 (vdW)<br>^{FH}R870 (vdW)	^{FH}R869 (vdW)	R869H/C/P***❷<br>R870P/L/H/C***❷<br>VUS: S866P/Y**❷❹<br>R869L*❷
^{BH}L873	^{FH}L873 (vdW)	^{FH}L873 (vdW)<br>^{FH}M877 (vdW)	^{FH}R870 (vdW)<br>^{FH}L873 (vdW)	R870P/L/H/C***❷<br>L873P**❷<br>M877K/L/I/T ***❷❹<br>VUS: None
^{BH}E874	^{FH}R869 (**H-bond**)	^{FH}R869 (Polar)	^{FH}R869 (**H-bond**)	R869H/C/P***❷<br>E874Q* ❸<br>VUS: R869L*❷<br>E874D*❷

(iii) BH/S2 interactions

**Cluster 1** – shown in Figure 2a (CryoEM structures)				
**BH Mesa**	**^{WT}ConfA S2**	**^{WT}ConfB S2**	**E525K S2**	**Patho/LP variants**
$_{Transducer}$R249	^{FH}N885 (Polar)	^{FH}N885 (Polar)	^{FH}Q892 (Polar);<br>^{FH}N885 (Polar)	R249P/L/Q**❷<br>VUS: N885Y*❹<br>Q892R/K*❷
$_{HO}$E448	^{FH}N885 (Polar)	No interaction	^{FH}N885 (Polar)	N885K**❷<br>VUS: N885Y*❹
HO-linker (K450-Q451-R452)	^{FH}V878<br>^{FH}L881	^{FH}V878 (vdW)<br>^{FH}L881 (vdW)<br>^{FH}Q882 (vdW)	^{FH}M877 (vdW)<br>^{FH}L881 (vdW)<br>^{FH}N885 (Polar)	K450N**❷<br>V878A/L**❷<br>L881R*❷<br>Q882E**❷<br>M877K/L/I/T ***❷❹<br>VUS: K450R/E**❷<br>Q451E**❷<br>P452S**❷<br>V878M**❹<br>L881M/P**❷<br>Q882R*❹<br>N885Y*❹
$_{HO-linker}$R453	^{FH}L881 CO (Polar)<br>^{FH}N885 (**H-bond**)	^{FH}L881 CO (**H-bond**)<br>^{FH}N885 (Polar)	^{FH}L881 (vdW)<br>^{FH}N885 (**H-bond**)	R453C/S/H/L***❷❹<br>L881R*❷<br>VUS: L881M/P**❷<br>N885Y*❹
**Cluster 2** – shown in Figure 2b (CryoEM structures)				
**BH Mesa**	**^{WT}ConfA S2**	**^{WT}ConfB S2**	**E525K S2**	**Patho/LP variants**
$_{HQ}$D522	No interaction	^{FH}E903 (Polar)	No interaction	E903Q/K**❷<br>VUS: D522V/G/E*❸❷❹<br>E903D/G ❷
$_{A-Loop}$E/K525	No interaction	No interaction	^{FH}E903 (Polar)	E525K**❸

	[BH environment:<br>^{BH}K484 (**H-bond**)<br>^{BH}N656 (**H-bond**)]	[BH environment:<br>^{BH}K484 (**H-bond**)<br>^{BH}N656 (**H-bond**)]	[BH environment:<br>^{BH}F480 (vdW)<br>^{BH}K484 (vdW)<br>^{BH}I521 (vdW)<br>^{BH}N656 (Polar)]	E903Q/K**❷<br>VUS: K484N*❷<br>I521T**❷<br>N656K**❷<br>E903D/G ❷
$_{A\text{-}Loop}$K526	No interaction	^{FH}D906 (**H-bond**)<br>^{FH}Q907 (**H-bond**)	^{FH}D906 (Polar)	D906G***❷<br>VUS: K526E*❷<br>Q907P/K**❷
$_{A\text{-}Loop}$P527-M528	^{FH}E903 (vdW)<br>^{FH}D906 (vdW)<br>^{FH}Q907 (vdW)<br>^{FH}K910 (vdW)	^{FH}Q907 (vdW)	^{FH}D906 (vdW)<br>^{FH}Q907 (vdW)<br>^{FH}K910 (vdW)	M528K❷<br>E903Q/K**❷<br>D906G***❷<br>VUS: P527T/H/L/S **❷❹<br>Q907P/K**❷<br>E903D/G ❷
$_{HLH\ loop}$M539-F540	^{BH}I913 (vdW)<br>^{BH}Q914 (vdW)<br>^{BH}A917 (vdW)	No interaction	^{BH}K190 (vdW)<br>^{BH}I913 (vdW)<br>^{BH}Q914 (vdW)<br>^{BH}A917 (vdW)	M539L/T/V❷<br>F540L**❸<br>I913T*❷<br>VUS: I913M/V*❷❹<br>Q914L/H**❷❹<br>A917I*❷
$_{Loop2}$K633	^{BH}E903-R904 mc (vdW)<br>^{BH}Q907 (**H-bond**)	^{BH}E903 (CO **H-bond**)<br>^{BH}R904 (vdW)	^{BH}D900 (**H-bond**)<br>^{BH}R904 (vdW)	E903Q/K**❷<br>R904C/H***❸<br>VUS: D900E/V*❷<br>E903D/G ❷<br>Q907P/K**❷
$_{Loop2}$G634	^{BH}E903 (vdW and **H-bond**)	No interaction	^{BH}E903 (**H-bond**)	E903Q/K**❷<br>VUS: G634C*❷<br>E903D/G ❷
$_{Loop2}$K635	No interaction	^{BH}D896 (vdW)<br>^{BH}D900 (vdW)	^{BH}D896 (**H-bond**)	D896N*❷<br>VUS: D900E/V*❷
$_{Loop2}$K637	^{BH}E902 (**H-bond**)	^{BH}A899 (vdW)<br>^{BH}E903 (vdW)	^{BH}E902 (**H-bond**)<br>^{BH}E903 (**H-bond**)<br>^{BH}D906 (**H-bond**)	E903Q/K**❷<br>D906G***❷<br>VUS: K637N**❹<br>E903D/G ❷
$_{vLoop2}$K640	^{BH}D906 (Polar)<br>^{BH}K910 (vdW)	No interaction	No interaction	D906G***❷<br>VUS: K640N/T*❷
$_{HW}$R652	^{FH}E903 (Polar)	No interaction	^{FH}E903 (vdW)<br>^{FH}Q907 (Polar)	R652G/K**❷<br>E903Q/K**❷<br>VUS: Q907P/K**❷<br>E903D/G ❷
$_{HW}$E653	No interaction	^{FH}D900 (Polar)	^{FH}D900 (Polar)	None<br>VUS: D900E/V*❷
$_{HW}$N656	No interaction	^{FH}D900 (Polar)	$_{FH}$E903 (**H-bond**)	E903Q/K**❷<br>VUS: N656K**❷<br>D900E/V*❷<br>E903D/G ❷
$_{HW}$K657	^{FH}D896 (**H-bond**)	^{FH}L889 (vdW)<br>^{FH}Q892 (vdW)<br>^{FH}D896, (vdW)	^{FH}Q892 (Polar)	K657N/Q*❷<br>D896N*❷<br>VUS: K657M**❹<br>L889P/H/V*❷<br>Q892R/K*❷
$_{HW}$T660	^{FH}Q895 (**H-bond**)<br>^{FH}D896 (**H-bond**)	^{FH}Q892 (**H-bond**)<br>^{FH}Q895 (vdW)<br>^{FH}D896 (**H-bond**)	^{FH}D896 (vdW)<br>^{FH}Q895 (Polar, vdW)	T660N**❷<br>D896N*❷<br>VUS: T660S**❷<br>Q892R/K*❷<br>Q895L/K/R/E **❷❹
$_{HW}$N661	^{FH}Q892 (**H-bond**)	^{FH}Q892 (vdW)	^{FH}Q892 (**H-bond,** vdW)	None<br>VUS: N661T*❷

				Q892R/K*❷

Cluster 3 – shown in Figure 3 (MD simulations and low resolution CryoEM)<br>Highly dynamic interface between S2 and ^{FH}Loop-2 revealed by MD simulations, confirmed by CryoEM. The most stable interactions observed are listed below, with the most frequently interacting residues in **bold**.<br>*NPI: no persistent interactions found during the time of the MD calculations.				
**BH Mesa**	**^{WT}ConfA S2**	**^{WT}ConfB S2**	**E525K S2**	**Patho/LP variants**
N408	NPI*	^{FH}R941	NPI*	None<br>VUS: N408K**❷<br>R941S/L/H*❷
K633	^{BH}E944	NPI*	NPI*	None<br>VUS: None
K637	**^{BH}D928**, ^{BH}E935	^{BH}E924, **^{BH}E927**, **^{BH}D928**, **^{BH}E931**	NPI*	E924G/K**❷<br>E927K**❷<br>D928V/N**❷<br>E931K**❷<br>E935K*❷<br>VUS: K637N**❹<br>D928G/A**❹<br>E935V/D/Q***❷❹
A638 (CO)	NPI*	^{BH}R925	NPI*	R925G*❷<br>VUS: A638D/V/S/T**❷
K639	NPI*	**^{BH}E931**, ^{BH}E935	NPI*	E931K**❷<br>E935K*❷<br>VUS: K639T/E/N**❷<br>E935V/D/Q***❷❹
K640	**^{BH}D928**, **^{BH}E931**, ^{BH}E932	^{BH}E924, **^{BH}D928**, **^{BH}E931**	NPI*	E924G/K**❷<br>D928V/N**❷<br>E931K**❷<br>VUS: K640N/T*❷<br>D928W/G/A**❷❹<br>M932I/T**❷

(iv) RLC/RLC interactions (from Cryo-EM maps) Figure 4A

Due to limiting resolution of the maps of this region, the interactions are less clear.

**^{BH}RLC/BH**	**^{FH}RLC**	**Conformation A**	**Conformation B**	**Patho/LP variants**
$_{RLC\ \alpha1}$Q38	$_{RLC\ loop2}$N78, F79, T80	Contacts	Contacts	T80N**❷<br>VUS: Q38K*❹<br>N78D**❷
$_{RLC\ \alpha1}$R40	$_{RLC\ \alpha1}$R40-G42	(**H-Bond** and vdW)	(**H-Bond**)	None<br>VUS: R40T/M**❷
$_{RLC\ \alpha2}$D51, A55	$_{RLC\ \alpha4}$T80-T84<br>$_{RLC\ nter}$F21	(vdW)<br>Contacts	(**H-Bond**)<br>(vdW)	A55P*❷<br>T80N**❷<br>VUS: D51Y*❹
$_{RLC\ \alpha2}$A55$_{CO}$, L56	$_{RLC\ nter}$S19	Contacts	A55$_{CO}$ (**H-Bond**)	A55P*❷<br>VUS: S19F/C**❷
$_{RLC\ \alpha2}$R58	$_{RLC\ nter}$S19	(**H-Bond**)	(**H-Bond**)	R58L/Q**❷<br>VUS: S19F/C**❷
$_{IQ}$P838	$_{RLC\ \alpha1}$Q23$_{CO}$, Q27	Contacts	Contacts	MYH7 / MYL2:<br>Q27R❷<br>MYH7 VUS:<br>P838Q/L***❷❹

				MYL2 VUS: Q27H*❹
$_{IQ}$L839	$_{RLC\ nter}$M20-F21<br>$_{RLC\ α1}$K30	Contacts	Contacts	MYH7 / MYL2: None<br>MYH7 VUS: None<br>MYL2 VUS: M20V** ❷
$_{IQ}$R845	$_{RLC\ α1}$Q27<br>$_{RLC\ α1}$Q25$_{CO}$, E28	(**H-Bond**)<br>Contacts	Contacts<br>Contacts	MYH7 / MYL2:<br>Q27R❷<br>MYH7 VUS:<br>R845T/K/G**❷<br>MYL2 VUS:<br>Q27H*❹<br>E28G/D**❷

(v) ELC/RLC interactions in BH and FH

^{BH}ELC	^{BH}RLC	Conformation A	Conformation B	Patho/LP variants
$_{ELC\ α1}$M59	$_{RLC\ linker3}$R129	No interaction	(**H-Bond**)	MYL2/MYL3: None<br>MYL2 VUS: None<br>MYL3 VUS: M59L*❷
$_{ELC-\ Loop\ 1}$ L60$_{CO}$, P65, E68	$_{RLC\ Linker\ 3}$ T125-Q126	Contacts	Contacts	MYL2/MYL3: None<br>MYH7 VUS: None<br>MYL2 VUS:<br>T125K/M**❷<br>Q126E**❷<br>MYL3 VUS:<br>L60V**❷<br>P65A/T
$_{ELC-\ loop1}$ R63, T64, P65	$_{RLC\ α6}$T125, Q126	Contacts	Contacts	MYL2/MYL3: None<br>MYL2 VUS:<br>T125K/M**❷<br>Q126E**❷<br>MYL3 VUS:<br>R63P/C/H**❷❹<br>T64I**❷<br>P65A/S*❷
$_{ELC-\ loop1}$P65, E68	$_{RLC\ linker3}$R129	Contacts<br>P65 (**H-Bond**)	Contacts	MYL2/MYL3: None<br>MYL2 VUS: None<br>MYL3 VUS: P65A/S*❷

Supplementary Table 4 – List of inter- and intramolecular interactions of the human cardiac relaxed filament (see **Supplementary Movie 11**). In black, interactions that are present in the refined model of the filament, in brown interactions that form upon the Long-term MD calculation. ❶ Note that the contacts in the PDB 8G4L model significantly differ. They are listed for comparison: interactions found in our model are shown in blue, while interactions absent from our model are shown in red. **Cardiomyopathy-causing mutations** predicted to destabilize the interfaces are indicated on the right. White background marks mutations altering direct contacts and blue background marks those close to the interface with indirect structural and/or dynamics effects. Each mutation is color-coded by type: HCM ②; DCM ③; other CM ④. The number of stars next to each mutation corresponds to the ClinVar annotation: "no classification or no assertion criteria provided" (0 stars); "criteria provided, single submitter" (1 star); "criteria provided, multiple submitters" (2 stars); "reviewed by expert panel" (3 stars); and "practice guideline" (4 stars). The Pathologic and likely pathologic mutations according to ClinVar are labeled "Patho/LP" and some Variants of Unknown Significance (VUS) are noted when they correlate with contacts proposed in this study. The most frequently interacting residues are shown in bold and # indicates the residues that can form attractive polar bonds. Interactions listed were analyzed from one of the molecular dynamics simulations, to provide an example of the diversity of contacts that can occur during the simulation. They do not correspond to an exhaustive list of contacts that the crowns can form.

Titin / CROWN 1 Tail

**Surface ID**<br>**TTN – Titin A-band**<br>^{Titin}**T9-T11 domains**<br>**778-1084**	**MYH7 – Crown 1:** MYH7 Tail: **912-981**<br>**Interface of low plasticity**<br>**anchoring the cc Tail of Crown 1, just downstream of Cluster 3**<br>S2 residues (912-931) form interactions with Titin rather than with $^{Cr1-FH-}$Loop2 (weak Cluster 3). S2 residues (939-981) interact with Titin including via salt-bridges between negatively charged ^{S2}Ring 3 residues (E944-E958)[9].	**Patho/LP variants**	**Figures/ Movies**
**T9 (778-877)**		K912R/Q* ❷ ❹<br>L915P** ❷<br>E921K** ❹<br>E924K/G** ❷<br>E927K** ❷<br>D928V/H/N** ❷ ❹<br>E931K** ❹<br>E949K*** ❷<br>with titin R933<br>D953H* ❷<br><br>MYH7 VUS:<br>K912N/M/E** ❷<br>E916D/K** ❷ ❹<br>D928G/A** ❹<br>E935V/D/Q*** ❷ ❹<br>K939Q/E** ❹<br>R941L/H/P/S** ❷ ❹<br>E949G/V**❹<br>K951R** ❷<br>R952T** ❷<br>D955Y* ❹<br>E958K** ❹<br>E965K/V**❷ ❹<br>A970S/V** ❷ ❹<br>K974N/R* ❷ ❹<br>L978P/V* ❷ ❹<br>E980K/D* ❹	Figure 6G
^{Titin}**K797** ❶	$^{FH-CC}$**E931**, $^{FH-CC}$E927, $^{FH-CC}$D928 #		
^{Titin}Q836	$^{FH-CC}$K912, vdW $^{FH-CC}$K920		
^{Titin}T837	vdW $^{FH-CC}$K920		
^{Titin}S839	vdW $^{FH-CC}$E924, $^{FH-CC}$E916, $^{FH-CC}$K920		
^{Titin}**K841** ❶	$^{FH-CC}$**E924**, $^{FH-CC}$**E927**, $^{FH-CC}$D928, $^{FH-CC}$E931#		
**T10 (878-977)**			
^{Titin}T893	$^{BH-CC}$K966		
^{Titin}K894	$^{BH-CC}$E965, $^{BH-CC}$K966		
^{Titin}**E911**	$^{FH-CC}$**K939**		
^{Titin}L913	vdW $^{FH-CC}$K942		
^{Titin}**R933**	$^{FH-CC}$**E946**, $^{FH-CC}$**E949**, $^{FH-CC}$R941, $^{FH-CC}$D945, $^{FH-CC}$E949, $^{FH-CC}$D953 #		
^{Titin}G936	$^{FH-CC}$E946		
^{Titin}**E937**	$^{BH-CC}$**K951**, $^{BH-CC}$R952, $^{FH-CC}$E946 #		
^{Titin}**R939** ❶	$^{BH-CC}$**D955**, $^{BH-CC}$**E958** #		
^{Titin}**R941** ❶	$^{BH-CC}$**E958**, $^{BH-CC}$D955, ^{FH}D953 #		
^{Titin}**E958** ❶	$^{FH-CC}$**K942**, $^{FH-CC}$R941 #		
^{Titin}N959	$^{FH-CC}$R941, $^{FH-CC}$K942		
^{Titin}A960	$^{FH-CC}$R941, $^{FH-CC}$K942		
^{Titin}**D977**	vdW $^{BH-CC}$**K966**		
**T11 (978-1084)**			
^{Titin}**P978**	$^{BH-CC}$**K966**		
^{Titin}Y980	$^{BH-CC}$K966		

[Titin]S1003	[BH-CC]E981		
[Titin]K1004	[BH-CC]E980, [BH-CC]E981<br>vdW [BH]N977		
[Titin]P1005	[BH-CC]N977		
**[Titin]I1006** ❶	vdW **[BH-CC]K974**, **[BH-CC]L978**, [BH-CC]N977		
**[Titin]Y1007** ❶	vdW **[BH-CC]K966**, **[BH-CC]A970**, **[BH-CC]K974**		
**PDB 8G4L**	**(*Dutta et al. 2023*[6], deposited model)**	PDB 8G4L shows fewer predicted contacts, with none involving domain T9 of titin, although some interactions with domains T10 and T11 of titin were predicted.	
	Titin D9 at 5 Å from Cr1 cc – no interaction		
[Titin-T10]R933, I935	[BH-CC]E946		
[Titin-T10]E937	[BH-CC]K951		
[Titin-T11]I1006	[BH-CC]K974, [BH-CC]N977		

CROWN 1

**Surface IA1**<br>**[MyBP-C]C8 domain**<br>**966-1063**	**Myosin HC MYH7, Crown 1:**<br>**[Cr1 FH]U50 278-325**<br>**Interface of low plasticity (vdW)**	**Patho/LP variants**	**Figures/ Movies**
[MyBP-C-C8]V991	**[HJ.HJ'-linker]N306**	MYH7:<br>R272G* ❷<br>R281T** ❹<br>I285T* ❸<br>L301R* ❷<br>L302Q* ❹<br>E317G* ❷<br>T318P* ❷<br>V320M/E** ❸ ❷<br>A321S* ❷<br>V338M*** ❷<br>Y350N* ❹<br><br>MyBPC3: None<br><br>MYH7 VUS:<br>R272S ❹<br>L277V/P* ❷ ❹<br>A279T/V** ❷<br>R281K** ❷<br>I285V/L** ❷ ❹<br>K293R* ❷<br>P295T** ❹<br>M300L/R/T ** ❹<br>L301P* ❹<br>T304S** ❹<br>N306H/K** ❷ ❹<br>P307L/S** ❹ ❷<br>F312L/V** ❷ ❹<br>I313V/F/L** ❷ ❹<br>S314P* ❷<br>Q315L/R* ❷ ❹<br>E317Q* ❷<br>T318N** ❷<br>S322T*** ❷<br>I323S/V/T/M** ❷ ❹<br>D324G* ❷<br>D325Y/G/V** ❷<br>A326S/G/P/T** ❷ ❹<br>L329F/I/P ❷<br>A331T/P ** ❹ ❸<br>D333V/G/E/N** ❷ ❹	Figure 6E<br>Supp. Movie 13<br>Left, Upper panel
**[MyBP-C-C8]N992** ❶	[HJ.HJ'-linker]N305, **[HJ.HJ'-linker]N306**		
[MyBP-C-C8]E1017	[HI-linker]K278, [HJ'-linker]T319, [HJ' -linker]V320		
[MyBP-C-C8]E1018	[HI-linker]K278 #		
**[MyBP-C-C8]S1020** ❶	**[HJ'-linker]Y308**, [HJ'.HK-linker]V320		
**[MyBP-C-C8]R1022** ❶	**[HI helix-]L290**, [HI helix-]S291, **[HJ.HJ'-linker]N305**, **[HJ'.HK-linker]T318**, **[HJ'.HK-linker]T319**, [HJ'.HK-linker]V320, [HJ'.HK-linker]A321, [HJ'.HK-linker]S322, [HJ'.HK-linker]I323, [HK helix-]D324, [HK helix-]D325 #		
[MyBP-C-C8]S1024	[HI linker-]K293, [HJ.HJ'-linker]N305		
[MyBP-C-C8]T1026	[HI helix-]K293		
[MyBP-C-C8]D1027	**[HI helix-]K293**, [HJ.HJ'-linker]N305 #		
[MyBP-C-C8]I1029 ❶	[HJ.HJ'-linker]N305		
[MyBP-C-C8]F1031 ❶	[HJ.HJ'-linker]N305, [HJ.HJ'-linker]N306, [HJ.HJ'-linker]P307, [HJ.HJ'-linker]Y308		
**[MyBP-C-C8]R1033** ❶	**[HI helix-]K278**, **[HJ'-linker]Y308**, [HJ' helix]I313, [HJ' helix]S314, [HJ' helix]Q315, [HJ' helix]G316, [HJ' helix]T318		

		A335S/V** ❷<br>V338A/E*** ❷<br><br>MYBPC3 VUS:<br>N992K** ❷<br>E1017K** ❹<br>E1018V/Q** ❷<br>R1022H/S ❷ ❹<br>S1024R** ❷<br>T1026I* ❷<br>D1027G/N** ❷ ❹<br>I1029N** ❹<br>R1033W/Q** ❷ ❹	
**PDB 8G4L**	**(*Dutta et al. 2023*[6], deposited model)**	PDB 8G4L significantly differs from the model obtained after energy minimization and the contacts observed during the MD simulation.	
R1022<br>I1029, F1031	T318, T319<br>N306, Y308		

**Surface IA2**<br>**Cr3 distal S2 and Cr2 LMM tail**<br>**1115-1134, 1602-1615**	**Myosin HC, Crown 1:**<br>**$^{Cr1\ FH}$PHHIS (HCM-Loop) 390-411 + 611**<br>**Interface of lowest plasticity**<br>**(vdW and charges)**<br>**(musical chairs)**	**Patho/LP variants**	**Figures/ Movies**
**Cr2 Tail**		MYH7:<br>A355T/S** ❹ ❷<br>R369Q*** ❸<br>Y386C*** ❹<br>D394E** ❷<br>G398R* ❷<br>R403W/G/Q/L***<br>❷ ❹<br>V406M** ❹<br>G407R/V/C** ❹ ❷<br>K611N* ❷<br>A1603P** ❹<br><br>MYH7 VUS:<br>A355P* ❷<br>R369W** ❹<br>N391S** ❷<br>D394H* ❹<br>K397N** ❹<br>G398E*** ❷<br>R403P** ❹<br>V406A* ❷<br>N408K** ❹<br>E409G/K* ❷ ❹<br>Y410H* ❷<br>V411F* ❹<br>E1116Q* ❷<br>E1119D** ❷<br>E1120A* ❷<br>E1121D/V** ❷<br>E1123K/A* ❷<br>R1126G/C/L/H** ❷❹<br>R1129T* ❷<br>K1131N* ❷<br>K1134R* ❷<br>A1603V/T** ❸ ❹<br>with Cr2 tail D1314	Supp. Movie 13 Right, Upper panel
**^{Cr2}A1603** ❶	**$^{HN-helix}$N391**		
**^{Cr2}R1606** ❶	**$^{HN-helix}$N391** #		
^{Cr2}E1610 ❶	$^{HN-helix}$K397, (^{Cr3}E1120) #		
**Cr3 Tail**			
^{Cr3}E1116	$^{HN-helix}$N391, $^{HU-helix}$K611, ^{Cr2}R1606 #		
**^{Cr3}E1119**	vdW **$^{HCM-loop}$Y410**, $^{HCM-loop}$R403, #<br>**$^{HCM-loop}$K405, $^{HU-helix}$K611** #		
^{Cr3}E1120 ❶	$^{HN-helix}$K397, $^{HN-helix}$D394, (^{Cr2}E1610) #		
^{Cr3}E1123 ❶	**$^{HCM-loop}$R403**, $^{HCM-loop}$K405, $^{HCM-loop}$Y410 #		
**^{Cr3}R1126** ❶	vdW **$^{HCM-loop}$Y410**, $^{HCM-loop}$N408		
**^{Cr3}T1127** ❶	**$^{HCM-loop}$V411**, **$^{HCM-loop}$N408**, $^{HCM-loop}$Y410		
^{Cr3}R1129	$^{HCM-loop}$N408		
^{Cr3}K1131 ❶	$^{HCM-loop}$E409 #		
^{Cr3}K1134 ❶	**$^{HCM-loop}$E409** #		

		R1606P/G/H/C**❷❹<br>E1610K** ❹	
**PDB 8G4L**	**(*Dutta et al. 2023*[6], deposited model)**	PDB 8G4L significantly differs from the model built here, showing different contacts and involving additional residues during the dynamic simulation	
$^{\text{Cr3}}$E1116<br>$^{\text{Cr3}}$E1119<br>$^{\text{Cr3}}$E1123<br>$^{\text{Cr3}}$R1126	$^{\text{HU-helix}}$K611<br>vdW $^{\text{HCM-loop}}$Y410<br>vdW $^{\text{HCM-loop}}$Y410<br>vdW $^{\text{HCM-loop}}$E409		

**Surface IB**<br>$^{\text{MyBP-C}}$**C5 domain**<br>**658-770**	**MYL2 Myosin RLC, Crown 1:** $^{\text{Cr1 FH}}$**RLC**<br>**Dynamic loop in the Interface**<br>**(vdW – Disordered C5 loop accommodates)**	**Patho/LP variants**	**Figures/ Movies**
$^{\text{MyBP-C-C5}}$**Q684** ❶<br>$^{\text{MyBP-C-C5}}$**I687** ❶<br><br>$^{\text{MyBP-C-C5}}$**T688** ❶<br>$^{\text{MyBP-C-C5}}$**G690** ❶<br>$^{\text{MyBP-C-C5}}$**K692** ❶<br><br>$^{\text{MyBP-C-C5}}$R696<br>$^{\text{MyBP-C-C5}}$**E710** ❶<br>$^{\text{MyBP-C-C5}}$**W711** ❶	$^{\text{RLC-Nlobe}}$**F53**<br>$^{\text{RLC-Nlobe}}$**A54**, $^{\text{RLC-Nlobe}}$L56, $^{\text{RLC-Nlobe}}$**G57**, $^{\text{RLC-Nlobe}}$**R58**<br>$^{\text{RLC-Nlobe}}$**G57**, $^{\text{RLC-Nlobe}}$**R58**<br>$^{\text{RLC-Nlobe}}$**R58**<br>$^{\text{RLC-Cter}}$**E163**, $^{\text{RLC-Cter}}$D166, $^{\text{RLC-Clobe}}$N154, $^{\text{RLC-Cter}}$G162, ^{Hook}E844 #<br>$^{\text{RLC-Clobe}}$D145, $^{\text{RLC-Clobe}}$D151 #<br>$^{\text{RLC-Nlobe}}$**R58**, ^{Hook}K835 #<br>^{Hook}**L839**, $^{\text{RLC-Nlobe}}$**F53**, $^{\text{RLC-Nlobe}}$**A54**, ^{Hook}K835	MYL2:<br>R58L/Q** ❷ ❹<br>H161R ❷<br>G162E** ❹<br>D166Y/V** ❹<br><br>MYBPC3:<br>D770H/N** ❷<br><br>MYH7:<br>K835T*❷<br><br>MYL2 VUS:<br>A54S/V** ❷ ❹<br>G57R/E** ❷ ❹<br>R58G ❷<br>D145E/G/Y/N** ❷❹<br>H161Q** ❷<br>E163Q/K* ❷<br>D166A/H/N** ❷ ❹<br><br>MYBPC3 VUS:<br>Q684K* ❹<br>T688M/K** ❷<br>G690V* ❹<br>N691Y* ❷<br>K692Q** ❹<br>P694R/L** ❷ ❹<br>W711S/L* ❷ ❹<br>D770E** ❷<br><br>MYH7 VUS:<br>K835M/T** ❷❹	Figure 6D Supp. Movie 11 Supp. Movie 12 and Supp. Movie 14 Left, Upper panel
**PDB 8G4L**	**(*Dutta et al. 2023*[6], deposited model)**	PDB 8G4L significantly differs from the model built here, showing different MyBP-C C5 loop and RLC/RLC interface	
$^{\text{MyBP-C-C5}}$T688, Q689<br>$^{\text{MyBP-C-C5}}$D709, E710<br>$^{\text{MyBP-C-C5}}$K715	^{RLC}L56, ^{RLC}D166cter<br>^{RLC}A55co, ^{RLC}G57<br>vdW ^{RLC}P838		

**Surface IC ***<br>$^{\text{Cr1 BH and FH}}$**S2**<br>**918-936**<br>**(Cluster 3)**	**Myosin HC, Crown 1:** $^{\text{Cr1-FH}}$ **Loop2 624-645**<br>Not built in PDB 8G4L<br>**Interface that forms upon Long-term MD calculations via Loop2 exploration**<br>**Highly dynamic interface**<br>Only Loop-2 is involved in Cluster 3	**Patho/LP variants**	**Figures/ Movies**

❶<br>[S2-BH]E921<br>[S2-BH]R925<br>[S2-BH]L926<br>[S2-FH]L926<br>[S2-BH]D928<br>**[S2-BH]**E929<br><br>[S2-FH]E929<br>**[S2-FH]E930**<br><br>[S2-BH]M932<br>[S2-BH]N933<br>[S2-FH]N933	[Loop2]K633, [Loop2]K637, [Loop2]K640, #<br>[Loop2]E632, [Loop2]K637, [Loop2]K639 #<br>[Loop2]A638<br>[Loop2]A638<br>[Loop2]K633, [Loop2]K637 #<br>[Loop2]K633, [Loop2]K635, **[Loop2]G636**, [Loop2]K637,<br>[Loop2]A638, **[Loop2]K639** #<br>[Loop2]K635, **[Loop2]K639** #<br>[Loop2]K628, [Loop2]K635, [Loop2]G636, **[Loop2]K639**,<br>[Loop2]K640 #<br>[Loop2]K633<br>[Loop2]K635<br>[Loop2]K635, **[Loop2]K639**	MYH7:<br>L620P* ❹<br>G641A** ❷<br>E921K* ❹<br>E924K/G** ❸ ❷<br>R925S/G* ❸ ❷<br>E927K** ❷<br>D928N/V/H** ❷❹<br>E930Q/K** ❸ ❹<br>E931K** ❹<br>with titin K933<br>E949K*** ❷<br>with titin R933<br><br>MYH7 VUS:<br>A629T/V** ❷<br>G634C* ❷<br>G636R/S* ❷ ❹<br>K637N* ❹<br>A638T/S/D/V** ❷ ❹<br>K639E/T/N** ❷<br>K640T/N* ❷<br>S642L/W*** ❷<br>L926V/P* ❷<br>D928G/A** ❹<br>E929Q/D** ❷<br>M932T/I** ❷ ❹	Figure 6G<br>Supp. Movie 11 and Supp. Movie 14 Right Upper panel

CROWN 3

Surface IIIA1<br>**[MyBP-C]C10 domain**<br>**1178-1274**	**Myosin HC, Crown 3:**<br>**[Cr3 FH]U50 292-350**<br>**Interface of medium plasticity**<br>**(vdW and few charges)**<br>Few contacts are present at this interface and they are created and lost along the long-term MD simulation	**Patho/LP variants**	**Figures/ Movies**
[MyBP-C-C10]K1242 ❶<br>[MyBP-C-C10]C1244<br>**[MyBP-C-C10]F1246**<br>**[MyBP-C-C10]V1272** ❶<br>[MyBP-C-C10]Q1274<br>Cter ❶	[HJ-helix]E296, vdW [HL-helix]E344 #<br>vdW [HK-helix]D337<br>vdW **[HK-helix]N334**, **[HK-helix]D337**, [HK-helix]V338<br>vdW **[HI-linker]K293**<br>vdW **[HK-linker]M330**, [HI-linker]K293	MYH7:<br>V338M*** ❷<br><br>MYBPC3:<br>L1238P** ❷<br>D1247G* ❹<br>G1260V* ❹<br><br>MYH7 VUS:<br>K293R* ❷<br>M330I** ❹<br>N334K/D* ❷ ❹<br>V338A/E*** ❷<br>E344K/V** ❷<br><br>MYBPC3 VUS:<br>K1240V/N* ❷ ❹<br>K1242E/N** ❷ ❹<br>C1244R/Y* ❷ ❹<br>F1246S** ❹<br>D1247Y/N** ❷ ❹	Figure 6B

		G1260D/S/R** ❷<br>V1272L/M/A* ❷ ❹	
**PDB 8G4L**	(*Dutta et al. 2023*[6], deposited model)	Fewer contacts in PDB 8G4L	
MyBP-C-C10 F1246	vdW HK-helix N334, HK-helix D337, HK-helix V338		

**Surface IIIA2**<br>**Cr2 LMM tail**<br>**Distal region**<br>**1700-1724**	**Myosin HC, Crown 3:**<br>**Cr3 FH U50 292-350 + 382-386**<br>**Interface of relatively low plasticity**<br>**(vdW and few polar interactions)**	**Patho/LP variants**	**Figures/ Movies**
Cr2-CC I1707 (CC8)<br>Cr2-CC E1708 (CC7) ❶<br>**Cr2-CC E1708 (CC8)** ❶<br>Cr2-CC E1711 (CC8) ❶<br>**Cr2-CC R1712 (CC7)**<br><br>**Cr2-CC Q1714 (CC8)** ❶<br><br>Cr2-CC S1718 (CC8) ❶<br>**Cr2-CC Q1719 (CC7)** ❶	vdW HJ-helix D299, HJ-helix M300<br>HM-helix Y386<br>**Cr2-CC K1416** #<br>vdW HJ-helix D299, HJ.HJ'-linker N305<br>**HM-helix Y386**, vdW **HJ-helix M300**, HJ-helix L302,<br>HJ-helix D299 #<br>**HJ-helix L302**, vdW HM-helix K383, HJ-helix D299,<br>HJ.HJ'-linker N305, HM-helix Y386<br>HJ.HJ'-linker N305<br>**HM-helix D382**	MYH7:<br>L302Q* ❹<br>K351E/T/N** ❷❺<br>D382N/G* ❷<br>S384Y ❹<br>Y386C*** ❷<br>L387F* ❷<br>R1712W/Q*** ❷<br><br>MYH7 VUS:<br>D299G/H/N* ❷<br>M300R/L/T** ❹<br>K351M* ❷<br>E379A* ❷<br>D382H/A* ❷ ❹<br>K383R*** ❷<br>S384P* ❷<br>L387V* ❷<br>K1416E** ❹<br>I1707F/ T/V** ❹<br>E1708K/V* ❷ ❹<br>E1711G/D/K** ❷ ❹<br>V1713M* ❹<br>Q1719R** ❹	Figure 6F
**PDB 8G4L**	(*Dutta et al. 2023*[6], deposited model)	Contacts differ significantly from those in PDB 8G4L.<br><br>Our model indicates that the charges on either side of the interaction can be repulsive (E1711 / D299)	
Cr2-CC E1705	HJ-helix M300		
Cr2-CC I1707 (CC8)	HJ-helix D299, HJ-helix M300, HJ-helix L302		
Cr2-CC E1708	HJ-helix M300		
Cr2-CC E1711	HJ-helix T304		
Cr2-CC R1712	HJ-helix L302		
Cr2-CC Q1714	HJ-helix L302, E379		
Cr2-CC L1715	HJ-helix T304		

**Surface IIIA3**<br>**MyBP-C C10 domain**<br>**1178-1274**	**Myosin HC, Crown 3:**<br>**Cr3 FH Loop1 198-216**<br>Not built in PDB 8G4L<br>**Proposed by dynamics**<br>**Dynamic loop in the Interface**<br>Intrinsically disordered loop that creates polar bonds (musical chairs), increasing the interaction surface while having high plasticity	**Patho/LP variants**	**Figures/ Movies**
❶<br>**MyBP-C-C10 N1217**	**Loop1 Q209**, Loop1 D208, Loop1 S210	MYH7:<br>V139L* ❷<br>R143Q/G/W***	Figure 6B,

$^{\text{MyBP-C-C10}}$**G1218**	vdW $^{\text{Loop1}}$**Q209**, $^{\text{Loop1}}$D208, $^{\text{Loop1}}$S210, $^{\text{Loop1}}$G212	❷ ❹<br>A199T/E/V** ❷ ❹<br>A200V** ❷<br>I201T** ❹<br>R204H** ❷<br>T215I** ❷<br>E217Q* ❹<br>V338M*** ❷<br><br>MYBPC3: None<br><br>MYH7 VUS:<br>I201F* ❷<br>R204C/S*** ❷ ❹<br>K206Q/R** ❷ ❹<br>D208A/V** ❷ ❹<br>Q209K/H** ❷<br>S210R* ❷<br>G212S/C* ❷❹<br>K213Q/N** ❹<br>V338A/E/L*** ❷ ❹<br><br>MYBPC3 VUS:<br>G1218A/V* ❷ ❹<br>D1220A/N** ❷ ❹<br>R1254G** ❹<br>E1261K/Q** ❹<br>R1263L/Q/P** ❷ ❹<br>E1265K/V* ❷ ❹<br>R1267H/G/C** ❹	Figure 7C, Supp. Movie 11 and Supp. Movie 14 Left, Lower panel
$^{\text{MyBP-C-C10}}$**L1219**	vdW $^{\text{Loop1}}$**Q209**,		
$^{\text{MyBP-C-C10}}$D1220	$^{\text{Loop1}}$K213 #		
$^{\text{MyBP-C-C10}}$I1250	vdW $^{\text{Loop1}}$Q209		
$^{\text{MyBP-C-C10}}$R1254	vdW $^{\text{Loop1}}$K206		
$^{\text{MyBP-C-C10}}$E1261	$^{\text{Loop1}}$K207 #		
$^{\text{MyBP-C-C10}}$**R1263**	$^{\text{Loop1}}$**Q209**, $^{\text{Loop1}}$D208, vdW $^{\text{Loop1}}$K207 #		
$^{\text{MyBP-C-C10}}$E1265	$^{\text{Loop1}}$K206, $^{\text{Loop1}}$K207 #		
$^{\text{MyBP-C-C10}}$**R1267**	$^{\text{Loop1}}$**D208**, $^{\text{Loop1}}$Q209 #		

**Surface IIIC**<br>**Cr3 S2 and Cr1 distal S2 CC**<br>**925-950 and 1040-1050**<br>**(Cluster 3)**	**Myosin HC, Crown 3:**<br>$^{\text{Cr3 FH}}$ **Loop2 624-645 and**<br>$^{\text{Cr3 FH}}$ **HCM-Loop 401-415**<br>Involvement of both elongated loops is consistent with CryoEM map<br>**Highly dynamic interface**<br>(Two elongated loops with musical chairs)	**Patho/LP variants**	**Figures/ Movies**
❶		MYH7:<br>M539L/T/ V** ❷ ❹<br>L620P* ❹<br>G641A** ❷<br>D928N/V/H** ❷ ❹<br>E930Q/K** ❸ ❹<br>E931K** ❹<br>with Cr1 tail K1026<br>E935K* ❷<br><br>MYH7 VUS:<br>G634C* ❷<br>G636R/S* ❷ ❹<br>K637N** ❹<br>A638T/S/D/V** ❷ ❹<br>K639E/T/N** ❷<br>K640T/N* ❷<br>S642L/W*** ❷<br>D928A/G** ❹<br>E929Q/D** ❷<br>M932I/T** ❷ ❹<br>E935V/Q/D*** ❷ ❹<br>T937S/I/N* ❷ ❹	Supp. Movie 11 and Supp. Movie 14 Right, Lower panel
$^{\text{Cr3-S2-BH}}$D928	$^{\text{Loop2}}$K640, $^{\text{Loop2}}$K637, #		
$^{\text{Cr3-S2-BH}}$**E929**	$^{\text{Loop2}}$**K639**, $^{\text{Loop2}}$K637 #		
$^{\text{Cr3- S2-FH}}$**E930**	$^{\text{Loop2}}$**K639**, $^{\text{Loop2}}$K640		
$^{\text{Cr3-S2-BH}}$E931	$^{\text{Loop2}}$K640		
$^{\text{Cr3-S2-BH}}$**M932**	vdW $^{\text{Loop2}}$**A638**		
$^{\text{Cr3-S2-FH}}$N933	$^{\text{Loop2}}$A638, $^{\text{Loop2}}$K639		
$^{\text{Cr3-S2-BH}}$E935	$^{\text{Loop2}}$K633, $^{\text{Loop2}}$KG34, $^{\text{Loop2}}$K637 #		
$^{\text{Cr3-S2-FH}}$T937	$^{\text{Loop2}}$K639		
$^{\text{Cr3-S2-FH}}$R941	$^{\text{Loop2}}$E628, $^{\text{Loop2}}$E632, $^{\text{Loop2}}$A638 #		
$^{\text{Cr3-S2-FH}}$E944	$^{\text{Loop2}}$K633 #		

		A938D/S** ❷<br>K939E/Q** ❹<br>R941L/H/P/S** ❷ ❹	
$^{\text{Cr3-S2-FH}}$E931<br>$^{\text{Cr3-S2-FH}}$A934<br>$^{\text{Cr3-S2-FH}}$T937<br>**$^{\text{Cr3-S2-FH}}$A938**<br>$^{\text{Cr3-S2-FH}}$K939 ❶<br>$^{\text{Cr3-S2-FH}}$R941<br>**$^{\text{Cr3-S2-FH}}$K942** ❶<br>$^{\text{Cr3-S2-FH}}$D945 ❶	$^{\text{HCM-loop}}$N408<br>$^{\text{HCM-loop}}$G407<br>vdW $^{\text{HCM-loop}}$T406<br>**$^{\text{HCM-loop}}$G407**, $^{\text{HCM-loop}}$N408, **$^{\text{HCM-loop}}$E409**<br>$^{\text{HCM-loop}}$E409 #<br>vdW $^{\text{HCM-loop}}$K405, $^{\text{HCM-loop}}$G407, $^{\text{HCM-loop}}$Y410<br>$^{\text{HCM-loop}}$N408, **$^{\text{HCM-loop}}$E409** #<br>$^{\text{HCM-loop}}$K413 #	MYH7:<br>R403W/G/Q/L*** ❷ ❹<br>V406M** ❹<br>G407R/V/C** ❷ ❹<br>E931K** ❹<br>R1045S/L*** ❷ with Cr1 tail K1026<br><br>MYH7 VUS:<br>R403P** ❹<br>V406A* ❷<br>N408K** ❹<br>E409G/K* ❷ ❹<br>Y410H* ❷<br>T937S/I/N* ❷ ❹<br>A938D/S** ❷<br>K939E/Q** ❹<br>R941L/H/P/S** ❷ ❹<br>K1042R** ❹<br>K1043R** ❹<br>R1045C*** ❷	Supp. Movie 11 and Supp. Movie 13 Right, Lower panel
$^{\text{Cr1-CC}}$K1042 ❶<br>$^{\text{Cr1-CC}}$K1043 ❶	$^{\text{HCM-loop}}$E409 #<br>$^{\text{HCM-loop}}$E409 #		
**PDB 8G4L**	**(*Dutta et al. 2023*[6], deposited model)**	PDB 8G4L and our model do not show interactions between the HCM loop and S2. In our refined model, the HCM loop is about 6 Å away from this docking surface, and only Loop-2 interacts in Cluster 3. However, additional interactions involving these regions appeared during MD simulations.	
$^{\text{S2}}$R941<br><br>No interaction with $^{\text{Cr1-CC}}$K1042-$^{\text{Cr1-CC}}$1043	$^{\text{HCM-loop}}$G407 CO, $^{\text{HCM-loop}}$N408<br>Loop-2 was not modelled in PDB 8G4L		

**Surface IIIB** * **$^{\text{MyBP-C}}$C9 domain**	**MyL2 Myosin RLC, Crown 3:**<br>**$^{\text{Cr3-FH}}$RLC-Nlobe and $^{\text{Cr3-FH}}$Hook**<br>Interface not seen in CryoEM map<br>**Dynamic interface – appears during the long-term MD calculation, musical chairs**	**Patho/LP variants**	**Figures/ Movies**
❶<br>$^{\text{MyBP-C-C9}}$Q1070<br>**$^{\text{MyBP-C-C9}}$D1071**<br>**$^{\text{MyBP-C-C9}}$R1073**<br>$^{\text{MyBP-C-C9}}$E1085<br>**$^{\text{MyBP-C-C9}}$K1087**<br>$^{\text{MyBP-C-C9}}$Q1090<br>**$^{\text{MyBP-C-C9}}$K1155**	^{RLC}Q38, ^{RLC}N39, ^{RLC}R40<br>**^{Hook}K841**, ^{RLC}Q38 #<br>**^{RLC}D51**, ^{RLC}T52, vdW ^{RLC}F53, ^{RLC}A54 #<br>^{RLC}T52<br>**^{RLC}D45**, **^{RLC}D48**, ^{RLC}D51, ^{RLC}Q38 #<br>^{RLC}Q38, ^{RLC}N39<br>**^{Hook}E844**, **^{Hook}E848** #	MYH7:<br>P838L/Q*** ❹❷<br>E846K** ❹ Hook with K837<br>K847E/T*** ❷<br>E848G** ❹<br><br>MYL2 / MYBPC3:<br>None<br><br>VUS– MYH7:<br>K841N** ❷<br>E846G* ❹<br><br>MYL2 VUS:<br>Q38R* ❹<br>R40M/T** ❷<br>D45N/E** ❷ ❹<br>D48Y/N/H/E** ❹	Figure 7B and Supp. Movie 13 Left, Lower panel

		D51Y* ❹<br>A54T/S/V** ❷<br><br>MYBPC3 VUS:<br>Q1070R/H** ❷ ❹<br>D1071A/V** ❷<br>R1073Q/W** ❹<br>Q1090R** ❹<br>K1155E** ❷	

CROWN 1 / CROWN 3

Intercrown – 1<br>**Myosin HC, Crown 3:**<br>$^{\text{Cr3-BH-RLC}}$**NTE**<br>**1-20**	**Myosin HC, Crown 1:**<br>$^{\text{Cr1 FH}}$**SH3, P-loop, Switch-II, Loop-3**<br>**Dynamic interface, only possible when both crowns are docked**	**Patho/LP variants**	**Figures/ Movies**
❶		MYL2:<br>F18L** ❷<br>E22K** ❹<br><br>MYH7:<br>I467N/T* ❷ ❹<br>I524T/M** ❷<br>G571R* ❷<br>G584R/V/S/C*** ❷ ❹<br>D587H* ❷<br><br>MYL2 VUS:<br>K4R* ❹<br>K5R/E** ❹<br>K7R* ❹<br>K8R** ❷<br>R9K* ❷<br>G11R* ❹<br>N16D/S** ❹ ❷<br>F18I ** ❷<br>S19F/C** ❹<br><br>MYH7 VUS:<br>E45G/K/D** ❷ ❹<br>E179A/K** ❹<br>F465L* ❹<br>F468L* ❹<br>D469E/N** ❸❷<br>I524V/N ** ❹<br>R567H/G*** ❸❷<br>N568D/S/K** ❷ ❹<br>I585V/F* ❷ ❹<br>D587N/Y** ❷<br>E677K* ❷<br>P687T/H** ❷ ❹	Figure 6C, Figure 7A, Supp. Movie 11 and Supp. Movie 15
$^{\text{RLC-NTE}}$K4	$^{\text{SH3}}$D42, $^{\text{Loop-3}}$R567 #		
$^{\text{RLC-NTE}}$**K5**	$^{\text{SH3}}$**D42**, $^{\text{SH3}}$**K43**, $^{\text{L50-anchors Loop-3}}$D587 #<br>^{NTE}**N686**, $^{\text{Loop-3}}$N564, $^{\text{Loop-3}}$R567, $^{\text{Loop-3}}$N568		
$^{\text{RLC-NTE}}$A6	$^{\text{Loop-3}}$R567, $^{\text{near Loop-3}}$I585		
$^{\text{RLC-NTE}}$**K7**	^{NTE}**P687**, $^{\text{Switch-II}}$F468, $^{\text{Switch-II}}$D469, $^{\text{L50-anchors Loop-3}}$D587 #		
$^{\text{RLC-NTE}}$**K8**	$^{\text{SH3}}$**E45**, ^{Wedge}Y582, $^{\text{near Loop-3}}$I585, $^{\text{L50-anchors Loop-3}}$D587 #		
$^{\text{RLC-NTE}}$R9	$^{\text{P-loop}}$**E179**, $^{\text{Switch-II}}$D469, ^{NTE}E677, #<br>$^{\text{Switch-II}}$F465, $^{\text{Switch-II}}$I467, $^{\text{Switch-II}}$F468,<br>^{Wedge}A583, ^{Wedge}I585		
$^{\text{RLC-NTE}}$G11	$^{\text{Switch-II}}$I467		
$^{\text{RLC-NTE}}$N16	$^{\text{Loop-3}}$G571		
$^{\text{RLC-NTE}}$S19	$^{\text{Switch-II}}$D469, $^{\text{Loop-3}}$N568, $^{\text{Loop-3}}$K570,<br>$^{\text{Loop-3}}$G571, $^{\text{Loop-3}}$K572		
$^{\text{RLC-NTE}}$F21	$^{\text{Loop-3}}$K570		
$^{\text{RLC-NTE}}$E22	$^{\text{Loop-3}}$N568, $^{\text{Loop-3}}$K570 #		
$^{\text{RLC-NTE}}$Q23	$^{\text{Loop-3}}$K570, $^{\text{Loop-3}}$G571		
**PDB 8G4L**	**Suggestions (*Dutta et al. 2023*[6], Fig. 6d)**	The NTE was not modeled in PDB 8G4L. While some suggestions of residues involved in possible contacts were provided, they are fewer and differ significantly from	
N-terminus RLC sequence	^{NTE}D42, ^{NTE}E45, ^{NTE}D74, $^{\text{Switch-II}}$E466, $^{\text{Switch-II}}$D469, $^{\text{L50-anchors Loop-3}}$D587, ^{NTE}E677, ^{NTE}D685		

		those proposed by MD simulations	

Possible transient interactions between Cr1 and Cr3<br>**Myosin HC, Crown 1:**<br>$^{\text{Cr1-FH}}$**Loop-3 557-570**	**MYL3 Myosin $^{\text{Cr3-BH}}$ELC, Crown 3:**<br>$^{\text{Cr3-BH}}$**ELC 42-53**<br>*Interface not seen in CryoEM map*<br>**Dynamic – appears during the long-term MD calculation, musical chairs**	**Patho/LP variants**	**Figures/ Movies**
❶<br>$^{\text{Loop-3}}$K559<br>$^{\text{Loop-3}}$R567<br>$^{\text{Loop-3}}$K565<br>$^{\text{Loop-3}}$A561<br>$^{\text{Loop-3}}$G558	**vdW ^{ELC}E45 which interacts with ^{ELC}K43**<br>^{ELC}E49 #<br>^{ELC}E49 #<br>vdW ^{ELC}P48<br>vdW ^{ELC}T47	MYL3 / MYH7: None<br><br>MYL3 VUS:<br>K43E* ❹<br>E45Q** ❹<br>F46L** ❹<br>T47I** ❷<br>P48R/L/A* ❷ ❹<br>E49D** ❹<br><br>MYH7 VUS:<br>G558S* ❹<br>A561T** ❹<br>R567H/G *** ❸❷	

Possible transient interactions between Cr1 and Cr3<br>**Myosin HC, Crown 1**	**Myosin MYH7, Crown 3:**<br>$^{\text{Cr3-BH}}$**Converter**<br>*Interface not seen in CryoEM map*<br>**Highly Dynamic – few contacts – appears during the long-term MD calculation**	**Patho/LP variants**	**Figures/ Movies**
❶<br>$^{\text{Cr1-FH-CC}}$**H969**<br>$^{\text{Cr1-FH-CC}}$**E972**<br>$^{\text{Cr1-FH-CC}}$**N973**<br>$^{\text{Cr1-FH-CC}}$**K976**	$^{\text{Cr3-BH}}$**Q734**<br>vdW $^{\text{Cr3-BH}}$**Q734,** $^{\text{Cr3-BH}}$**D737**<br>vdW $^{\text{Cr3-BH}}$**Q734,** $^{\text{Cr3-BH}}$**D737**<br>$^{\text{Cr3-BH}}$**D737** #	MYH7:<br>Q734P/E* ❷<br>S738R ❷<br><br>MYH7 VUS:<br>D737Y** ❷<br>S738T/N*** ❷ ❹<br>E972D** ❹<br>K976E* ❷	Figure 6G

Supplementary Table 5 – Comparison of the distinct interactions of the FH and BH of Cr1 and Cr3. This table indicates how the structural elements of the myosin head can be engaged in different interactions depending on whether they are part of FH or BH, and Cr1 or Cr3. In yellow background, the interfaces stabilizing the heads within the IHM, including in green background the Cluster 3 interactions that involve Loop-2; in light blue, the interfaces stabilizing the docking of $_{Car}$IHM in distinct environments corresponding to Crown 1 or Crown 3. In pink, regions that are not involved in interactions at the start of the simulation but engage during the simulation. For the BH heads of Cr1 and Cr3, the surface of SH3, U50 HI-HK helices and Loop-3 are not involved in any interaction.

Crown 1 – FH		Crown 3 – FH	
^{Cr1}FH SH3 (32-80)	Intercrown<br>**Highly dynamic**<br>^{Cr3}BH RLC NTE (1-23)		
		**^{Cr3}FH Loop-1**<br>(198-216)	**Surface IIIA3**<br>**MyBP-C C10**<br>**Highly dynamic**
**^{Cr1}FH U50**<br>**HI.HK Helix region**<br>(278, 290-293, 304-308, 314-325)	**Surface IA1**<br>**MyBP-C C8**<br>Interface of lower plasticity (vdW)	**^{Cr3}FH U50**<br>**HI.HK Helix region**<br>(292-350, 382-386)	**Surface IIIA1**<br>**MyBP-C C10**<br>**Few contacts / Medium plasticity**<br>**Surface IIIA2**<br>**LMM Cr2 cc** (1700-1724)<br>**Relatively low plasticity**
^{Cr1}FH PHHIS<br>386-394 (near Loop-4)	**Surface IA2**<br>Close to the ^{Cr2} LMM coiled-coils<br>(**1115-1134, 1602-1615**)		
**^{Cr1}FH PHHIS (HCM-loop)**	**^{Cr3}LMM coiled-coil (aa 1120-1134)**	**^{Cr3}FH PHHIS (HCM-loop)**	**Surface IIIB'**<br>^{Cr3} **S2 (Cluster 3)**<br>**Dynamic**
^{Cr1}FH Transducer	^{Cr1}BH PHHIS (HCM-loop)	^{Cr3}FH Transducer	^{Cr3}BH PHHIS (HCM-loop)
^{Cr1}FH Loop-3	Intercrown<br>^{Cr3}BH RLC NTE **(intercrown)**		
^{Cr1}FH Loop-2	**Surface IC'**<br>^{Cr1} S2 **(Cluster 3) Highly dynamic**	^{Cr3}FH Loop-2	**Surface IIIC'**<br>^{Cr3} **S2 (Cluster 3) Dynamic**
^{Cr1}FH Relay and Converter	^{Cr1}BH PHHIS (379-393, Loop4)	^{Cr3}FH Relay and Converter	^{Cr3}BH PHHIS (379-393, Loop4)
^{Cr1}FH ELC C-lobe	^{Cr1}BH PHHIS (HU HV helices)	^{Cr3}FH ELC C-lobe	^{Cr3}BH PHHIS (HU HV helices)
^{Cr1}FH RLC NTE	^{Cr1}BH RLC N-lobe	^{Cr3}FH RLC NTE	^{Cr3}BH RLC N-lobe
^{Cr1}FH RLC N-lobe	^{Cr1}BH RLC N-lobe	^{Cr3}FH RLC N-lobe	^{Cr3}BH RLC N-lobe, NTE
^{Cr1}FH RLC NTE, C- lobe	**Surface IIB**<br>**MyBP-C C5**	*^{Cr3}FH RLC N-lobe*	Surface IIIB'<br>*MyBP-C C9*

Crown 1 – BH		Crown 3 – BH	
^{Cr1}BH Loop-1	^{Cr1}BH ELC and RLC C-lobes (stabilize lever priming in BH)	^{Cr3}BH Loop-1	^{Cr3}BH ELC and RLC C-lobes (stabilize lever priming in BH)
^{Cr1}BH PHHIS (379-393, Loop-4)	^{Cr1}FH Relay and Converter	^{Cr3}BH PHHIS (379-393, Loop-4)	^{Cr3}FH Relay and Converter
**^{Cr1}BH PHHIS (HCM-loop)**	**^{Cr1}FH Transducer**	**^{Cr3}BH PHHIS (HCM-loop)**	**^{Cr3}FH Transducer**
^{Cr1}BH Transducer	^{Cr1} S2 (Cluster 1)	^{Cr3}BH Transducer	^{Cr3} S2 (Cluster 1)
^{Cr1}BH L50 (A-loop, Loop-2)	^{Cr1} S2 (Cluster 2)	^{Cr3}BH L50 (A-loop, Loop-2)	^{Cr3} S2 (Cluster 2)
		^{Cr3}BH Converter	Transient interactions<br>*Distal Cr1 S2 (around aa 969)*
^{Cr1}BH ELC C-lobe	^{Cr1}BH Transducer (β-bulge Loop1)	^{Cr3}BH ELC C-lobe	^{Cr3}BH Transducer: β-bulge Loop-1
		^{Cr3}BH RLC NTE	Intercrown<br>^{Cr1}FH SH3, ^{Cr1}FH Loop-3
^{Cr1}BH RLC N-lobe	^{Cr1}FH RLC N-lobe	^{Cr3}BH RLC N-lobe	^{Cr3}FH RLC N-lobe
^{Cr1}BH RLC C-lobe	^{Cr1}BH Loop-1, ^{Cr1}BH ELC N-lobe	^{Cr3}BH RLC C-lobe	^{Cr3}BH Loop-1, ^{Cr3}BH ELC N-lobe

References:

1. Grinzato, A. *et al.* Cryo-EM structure of the folded-back state of human β-cardiac myosin. *Nat Commun* **14**, 3166 (2023).
2. Pylypenko, O. & Houdusse, A. M. Essential "ankle" in the myosin lever arm. *Proc. Natl. Acad. Sci. U.S.A.* **108**, 5–6 (2011).
3. Scarff, C. A. *et al.* Structure of the shutdown state of myosin-2. *Nature* **588**, 515–520 (2020).
4. Yang, S. *et al.* Cryo-EM structure of the inhibited (10S) form of myosin II. *Nature* **588**, 521–525 (2020).
5. Heissler, S. M., Arora, A. S., Billington, N., Sellers, J. R. & Chinthalapudi, K. Cryo-EM structure of the autoinhibited state of myosin-2. *Sci. Adv.* **7**, eabk3273 (2021).
6. Dutta, D., Nguyen, V., Campbell, K. S., Padrón, R. & Craig, R. Cryo-EM structure of the human cardiac myosin filament. *Nature* **623**, 853–862 (2023).
7. Meng, E. C. *et al.* UCSF ChimeraX : Tools for structure building and analysis. *Protein Science* **32**, e4792 (2023).
8. Auguin, D. *et al.* Omecamtiv mecarbil and Mavacamten target the same myosin pocket despite opposite effects in heart contraction. *Nat Commun* **15**, 4885 (2024).
9. Blankenfeldt, W., Thomä, N. H., Wray, J. S., Gautel, M. & Schlichting, I. Crystal structures of human cardiac β-myosin II S2-Δ provide insight into the functional role of the S2 subfragment. *Proc. Natl. Acad. Sci. U.S.A.* **103**, 17713–17717 (2006).
