# Supplementary material for: Dynamics of the β-cardiac myosin auto-inhibited state explain cardiomyopathy pathogenesis
Source: Nat Commun. 2026 Jun 4;17:5502. doi: 10.1038/s41467-026-73572-5 (PMC13287786; doi:10.1038/s41467-026-73572-5)
Supplement: Supplementary file 2 — Description of Additional Supplementary Files [file 41467_2026_73572_MOESM2_ESM.pdf]

Title: Supplementary Data 1

Description: Raw data of the molecular dynamics simulations experiments of isolated $_{Car}$IHM.

For the three conditions (WT, E525K, and E903K), raw RMSD values as a function of time are provided and plotted (Raw RMS). Distances between residue 525 and neighboring negatively charged residues of the S2 coiled-coil are also calculated as a function of time for the three conditions (Dist). For each condition, two independent simulations were performed (see Methods), and raw data from the replicates are shown here, demonstrating the reproducibility of the phenomena described in the text.

Title Supplementary Data 2

Description: Raw data of the molecular dynamics simulations experiments of relaxed thick filament.

For each chain, the RMSD data are shown as function of time in the two independent replicates. Only replicate 1 is discussed in the text, but the data shown here demonstrate the reproducibility of the phenomena described.

Title Supplementary Data 3

Description: Raw data of the molecular dynamics simulations experiments of relaxed thick filament.

For each chain, the RMSF data are shown as function of time in the two independent replicates. Only replicate 1 (Traj1) is discussed in the text, but the data shown here demonstrate the reproducibility of the phenomena described.

Title Supplementary Data 4

Description: Intercrown interface during all-atom molecular dynamics simulations 1 and 2 of the relaxed thick filament.

Residues involved in the interface are represented at representative time points.

Title: Supplementary Movie 1

Description: Flexibility of WT $_{Car}$IHM and E525K-IHM in CryoEM grids as calculated by 3DFlex.

The 41 maps from 3DFlex Generate (see Methods) are shown, frame by frame, side by side for WT and E525K. Colors are identical to those in Figure 1B**.** Note that, to capture the subdomains motions, the resolution of the 3DFlex maps is limited to 4.9 Å, much lower than that of the final deposited maps used to refine the model.

The 41 maps from 3DFlex Generate (see Methods) are shown, frame by frame, side by side for WT and E525K. Colors are identical to those in Figure 1B**.** Note that, to capture the subdomains motions, the resolution of the 3DFlex maps is limited to 4.9 Å, much lower than that of the final deposited maps used to refine the model.

Title: Supplementary Movie 2

Description: Variability of the interfaces in two conformations ^{WT}ConfA and ^{WT}ConfB.

(A) Comparison of the FH/BH interface involving the HCM-loop (red backbone: residues 400-416). The same structural elements are involved in this interface, but they do not form the same contacts, indicating plasticity in the BH/S2 interface. **(B-D)** Comparison of the interactions involving S2 near the ^{BH}Loop-2 (yellow backbone: residues 628-646) and the ^{BH}A-loop (dark green: residues 526-529) in three different orientations. The corresponding CryoEM density is shown in Supplementary Fig. 8. **(C)** No contact with S2 is made by $^{BH\text{-}A\text{-}loop}$E525 in these two structures. **(D)** ^{BH}Loop-2 forms distinct contacts in the two structures as it accommodates to the change in S2 orientation. A-loop: activation-loop.

Title: Supplementary Movie 3

Description: Movement of the S2 in WT $_{Car}$IHM and E525K-$_{Car}$IHM.

Part 1: the same 3DFlex maps used in Movie 1 were superimposed based on the BH. A 90° rotation and clipping of the BH outline the up-and-down movement of the S2. The BH helix does not always move in phase with the FH helix and the Hook also twists, suggesting possible adjustment in the register within the IHM itself. **Part 2:** Ten maps from masked 3DVA focusing on the S2 of E525K-$_{Car}$IHM looping back and forth. Starts with the back view of the motif to show the movement of the S2, outlining Clusters 0 and 3 at high contour level, then rotates to show Clusters 1 and 2. Contour level is then decreased to better show Cluster 3 and ^{BH}Loop-2 interactions with the S2. A final rotation to the front view of the motif shows ^{BH}Loop-1 and Cluster 3.

Title: Supplementary Movie 4

Description: Clusters 1 and 2 in ^{WT}ConfA, ^{WT}ConfB and E525K.

Two panels illustrate the interactions occurring in Cluster 1 (Left) and Cluster 2 (Right) in the three $_{Car}$IHM CryoEM structures in the same orientation.

Title: Supplementary Movie 5

Description: All-atom molecular dynamics simulations of wild-type conformation A (ConfA-WT), conformation A with the DCM-causing mutation E525K (ConfA-E525K) and conformation A with the HCM-causing mutation E903K (ConfA-E903K), global view.

Top and bottom rows: two different views rotated 180° in the y axis for each run. For clarity, only the backbone is shown. The duration of each simulation was 135 ns. The arrow near the distal region of the coiled-coil indicates the transient interactions that occur between ^{FH}Loop-2 and S2 in Cluster 3.

Title: Supplementary Movie 6

Description: Close-up of Cluster 2 in the all-atom molecular dynamics simulations of ConfA-WT, ConfA-E525K and ConfA-E903K.

Side chains of Cluster 2 and selected neighboring residues are shown. Polar interactions are indicated by blue dashes. Van der Walls radii of K525 and K903 are outlined in their respective panels.

Title: Supplementary Movie 7

Description: Close-up of the dynamic interface between ^{FH}Loop-2 and the S2 coiled-coil in all-atom molecular dynamics simulations of ConfA-WT.

The interface is formed by labile polar interactions which, coupled with the flexibility of Loop-2, create a dynamic network of transient contacts. Residues involved in the interactions are displayed in each frame.

Title: Supplementary Movie 8

Description: Close-up of Cluster 3 in the all-atom molecular dynamics simulations of ConfA-WT and ConfA-E525K.

Side chains of Cluster 3 are shown. Polar interactions are indicated by blue dashes. Although not present at the start of the simulation, transient interactions between ^{FH}Loop-2 and S2 occur rapidly in the two conditions ConfA-WT and ConfA-E525K.

Title: Supplementary Movie 9

Description: Improved structure of the Hook based on 3DFlex Reconstruct.

Starts with the previously published cryoEM map of the C-zone of the thick filament, showing the corresponding structure of the RLCs in PDB 8G4L, outlining $_{\mathrm{RLC}}$Ser15 in the RLC-RLC and intercrown interfaces. Then switches to the improved map from 3DFlex Reconstruct and morphing from PDB 8G4L to the new E525K-$_{\mathrm{Car}}$IHM structure. Model-to-model morphing was performed with ChimeraX which does not apply realistic restrains, simply to illustrate the incompatibility of PDB 8G4L with the improved map, and the improvement in fitting with the new structure. The movie ends with alternating superposition of 8G4L in grey on the new structure in colors.

Title: Supplementary Movie 10

Description: ^{WT}ConfB corresponds to one of the conformations that the cardiac myosin can adopt when free in solution, but it is not compatible with docking on the filament.

^{WT}ConfA and ^{WT}ConfB were docked in the CryoEM map of the relaxed filament (EMD-29722) via their S2 domains. While ^{WT}ConfA can fit in Crown 1 and Crown 3, ^{WT}ConfB goes out of density (purple dotted circles), losing the contacts that stabilize $_{\mathrm{Car}}$IHM in the filament.

Title: Supplementary Movie 11

Description: Long-term all-atoms molecular dynamics calculations of the human cardiac relaxed filament.

Part 1 (0 to 45 s): fit of the initial model in the CryoEM map of the relaxed filament (EMD-29722) with completion and optimization of the regions with missing density as described in Methods. **Part 2** (45 s to 1 m 34 s): Dynamics of the overall filament and close-up of selected interfaces during the 135 ns simulation.

Title: Supplementary Movie 12

Description: **^{FH}RLC/$^{\mathrm{MyBP\text{-}C}}$C5 interface, comparison with 8G4L**.

Zoom on the Cr1 RLC/RLC interface in the refined model of the human relaxed cardiac thick filament and how it interacts with the C5 subdomain of MyBP-C. It is compared to the initial low-resolution model (PDB 8G4L). Some disordered regions such as $^{\mathrm{MyBP\text{-}C}}$C5 loop (690-708, in pale orange) were not part of the previous model but were rebuilt by our approach: we can now precisely describe the interface. Another major difference in this region is the position of the N-terminal extension of the ^{FH}RLC (^{RLC}NTE) that is directly part of the RLC/RLC interfaces in 8G4L but not in our model.

Title: Supplementary Movie 13

Description: Interfaces stabilizing the docking of Cr1 and Cr3 during the all-atom molecular dynamics calculations (part 1).

Representative snapshots during the all-atom molecular dynamics calculations. Simulation times are indicated on the top left of each frame. Side chains of residues involved in contacts are shown as sticks, polar contacts as red dashes. The names of the selected interfaces (also described in Supplementary Table 4) are shown. All interactions illustrate the role of musical chairs in these interfaces. Some interfaces are relatively stable during the time-course: ($^{\mathrm{MyBP\text{-}C}}$C8/$_{\mathbf{Cr1}}$^{FH}U50; $^{\mathrm{MyBP\text{-}C}}$C5/$_{\mathbf{Cr1}}$^{FH}RLC), the $^{\mathrm{MyBP\text{-}C}}$C9/$_{\mathbf{Cr3}}$RLC and the $^{\mathrm{MyBP\text{-}C}}$C10/$_{\mathbf{Cr3}}$$^{\mathbf{FH}}$Loop-1 interfaces form during this simulation and stay relatively stable; some other interfaces are more ephemerous: notably those in involving Loop-2 and the HCM-loop in Cluster 3. In Supplementary Movie 13: $^{\mathrm{MyBP\text{-}C}}$C8/$_{\mathbf{Cr1}}$^{FH}U50; $^{\mathrm{MyBP\text{-}C}}$C9/$_{\mathbf{Cr3}}$RLC; ^{FH}HCM-loop (outlined in red) is involved in different interactions to stabilize Cr1 (with LMM) and Cr3 (with S2, as strong interactions are formed in Cluster 3).

Title: Supplementary Movie 14

Description: Interfaces stabilizing the docking of Cr1 and Cr3 during the all-atom molecular dynamics calculations (part 2).

Representative snapshots during the all-atom molecular dynamics calculations. Simulation times are indicated on the top left of each frame. Side chains of residues involved in contacts are shown as sticks, polar contacts as red dashes. The names of the selected interfaces (also described in Supplementary Table 4) are shown. All interactions illustrate the role of musical chairs in these interfaces. Some interfaces are relatively stable during the time-course: ($^{\text{MyBP-C}}$C8/$_{\textbf{Cr1}}^{\text{FH}}$U50; $^{\text{MyBP-C}}$C5/$_{\textbf{Cr1}}^{\text{FH}}$RLC), the $^{\text{MyBP-C}}$C9/$_{\textbf{Cr3}}$RLC and the $^{\text{MyBP-C}}$C10/$_{\textbf{Cr3}}^{\textbf{FH}}$Loop-1 interfaces form during this simulation and stay relatively stable; some other interfaces are more ephemerous: notably those in involving Loop-2 and the HCM-loop in Cluster 3. In Supplementary Movie 14: $^{\text{MyBP-C}}$C5/$_{\textbf{Cr1}}^{\text{FH}}$RLC; $^{\text{MyBP-C}}$C10/$_{\textbf{Cr3}}^{\textbf{FH}}$Loop-1; ^{FH}Loop-2 (outlined in yellow) is involved in strong interactions in cluster 3 for Cr3, but weak interactions with Cr1.

Title: Supplementary Movie 15

Description: The intercrown interface during the all-atom molecular dynamics calculations.

Representative snapshots during the all-atom molecular dynamics calculations. Simulation times are indicated on the top left of each frame. Side chains of residues involved in contacts are shown as sticks, polar contacts as red dashes. The intrinsically disordered N-terminal extension of $^{\text{Cr3-BH}}$RLC (aa 1-20) is free and it interacts with multiple residues of the docked Cr1 FH $_{\text{Car}}$IHM head during the all-atoms simulation. The $^{\text{BH-RLC}}$Ser15 whose phosphorylation regulates force production is outlined in spheres.
