# Supplementary material for: Dynamics of the β-cardiac myosin auto-inhibited state explain cardiomyopathy pathogenesis
Source: Nat Commun. 2026 Jun 4;17:5502. doi: 10.1038/s41467-026-73572-5 (PMC13287786; doi:10.1038/s41467-026-73572-5)
Supplement: Supplementary file 6 — Supplementary Data 4 [file 41467_2026_73572_MOESM6_ESM.docx]

| **Time (ns)** | **Simulation 1** | | **Simulation 2** | |
| --- | --- | --- | --- | --- |
| **0.6** |  |  | ^RLC-NTE^K4 | ^SH3^D42 |
|  |  |  | ^RLC-NTE^K5 | ^SH3^K43 |
|  |  |  | ^RLC-NTE^K7 | E677 |
|  |  |  | ^RLC-NTE^K8 | ^SH3^E45 |
| **0.8** | ^RLC-NTE^K4 | ^SH3^D42 |  |  |
|  | ^RLC-NTE^K5 | ^SH3^D42 |  |  |
|  | ^RLC-NTE^K8 | ^SH3^E45 |  |  |
| **2** |  |  | ^RLC-NTE^K5 | ^NTE^D685, |
|  |  |  | ^RLC-NTE^K7 | E677 |
|  |  |  | ^RLC-NTE^K8 | ^SH3^E45 |
|  |  |  | ^RLC-NTE^Q23 | ^Loop-3^K570 |
| **4** | ^RLC-NTE^R9 | ^NTE^E677 |  |  |
| **8** |  |  | ^RLC-NTE^M1 | ^SH3^K43 |
|  |  |  | ^RLC-NTE^K5 | ^NTE^E677, ^NTE^D685 |
|  |  |  | ^RLC-NTE^K7 | ^P-loop^E179 |
|  |  |  | ^RLC-NTE^K8 | ^SH3^E45 |
|  |  |  | ^RLC-NTE^E22 | ^Loop-3^K570 |
| **9** |  |  | ^RLC-NTE^K5 | ^NTE^E677, ^NTE^D685 |
|  |  |  | ^RLC-NTE^K7 | ^P-loop^E179 |
|  |  |  | ^RLC-NTE^K8 | ^SH3^E45 |
|  |  |  | ^RLC-NTE^F18 | ^Loop-3^K570 |
|  |  |  | ^RLC-NTE^E22 | ^Loop-3^K570 |
| **14** | ^RLC-NTE^S19 | ^Loop-3^K570 | ^RLC-NTE^K5 | ^NTE^D685 |
|  | ^RLC-NTE^K8 | ^near Loop-3^I585 | ^RLC-NTE^K7 | ^P-loop^E179 |
|  | ^RLC-NTE^R9 | ^Switch-II^I467, ^Switch-II^F465 | ^RLC-NTE^K8 | ^SH3^Q44, ^SH3^E45 |
|  |  |  | ^RLC-NTE^F18 | ^Loop-3^K570 |
|  |  |  | ^RLC-NTE^S19 | ^Loop-3^R567 |
|  |  |  | ^RLC-NTE^F21 | ^Loop-3^K570 |
| **16** |  |  | ^RLC-NTE^K5 | ^NTE^D685 |
|  |  |  | ^RLC-NTE^K7 | ^P-loop^E179 |
|  |  |  | ^RLC-NTE^K8 | ^SH3^D42, ^SH3^Q44, ^SH3^E45 |
|  |  |  | ^RLC-NTE^R9 | ^SH3^D42, |
|  |  |  | ^RLC-NTE^F18 | ^Loop-3^K570 |
|  |  |  | ^RLC-NTE^S19 | ^Loop-3^R567, ^Loop-3^K570 |
|  |  |  | ^RLC-NTE^F21 | ^Loop-3^K570 |
| **20** | ^RLC-NTE^S19 | ^Loop-3^K570 | ^RLC-NTE^K5 | ^NTE^D685 |
|  | ^RLC-NTE^K5 | ^L50-anchors Loop-3^D587 | ^RLC-NTE^K7 | ^P-loop^E179 |
|  | ^RLC-NTE^R9 | ^Switch-II^I467, | ^RLC-NTE^K8 | ^SH3^E45 |
|  |  |  | ^RLC-NTE^F18 | ^Loop-3^K570 |
|  |  |  | ^RLC-NTE^S19 | ^Loop-3^R567 |
|  |  |  | ^RLC-NTE^F21 | ^Loop-3^K570 |
| **24** |  |  | ^RLC-NTE^K5 | ^NTE^D685 |
|  |  |  | ^RLC-NTE^K8 | ^SH3^E45 |
|  |  |  | ^RLC-NTE^F18 | ^Loop-3^K570 |
|  |  |  | ^RLC-NTE^S19 | ^Loop-3^R567 |
|  |  |  | ^RLC-NTE^E22 | ^Loop-3^K570 |
| **25** | ^RLC-NTE^K5 | ^Loop-3^R567, ^L50-anchors Loop-3^D587 | ^RLC-NTE^K8 | ^SH3^K43, ^SH3^E45 |
|  | ^RLC-NTE^K8 | ^L50-anchors Loop-3^D587 | ^RLC-NTE^R9 | ^SH3^D41 |
|  | ^RLC-NTE^R9 | ^Switch-II^I467, | ^RLC-NTE^F18 | ^Loop-3^K570 |
|  |  |  | ^RLC-NTE^S19 | ^Loop-3^R567 |
|  |  |  | ^RLC-NTE^E22 | ^Loop-3^K570 |
| **27** |  |  | ^RLC-NTE^M1 | ^SH3^K43 |
|  |  |  | ^RLC-NTE^K5 | ^NTE^D685 |
|  |  |  | ^RLC-NTE^K7 | ^P-loop^E179 |
|  |  |  | ^RLC-NTE^K8 | ^SH3^K43, ^SH3^E45 |
|  |  |  | ^RLC-NTE^F18 | ^Loop-3^K570 |
|  |  |  | ^RLC-NTE^S19 | ^Loop-3^R567 |
|  |  |  | ^RLC-NTE^F21 | ^Loop-3^K570 |
| **28** | ^RLC-NTE^K5 | ^L50-anchors Loop-3^D587 |  |  |
|  | ^RLC-NTE^K8 | ^near Loop-3^I585, ^L50-anchors Loop-3^D587 |  |  |
|  | ^RLC-NTE^R9 | ^Switch-II^I467, |  |  |
| **31** | ^RLC-NTE^Q23 | ^Loop-3^G571 |  |  |
|  | ^RLC-NTE^K8 | ^L50-anchors Loop-3^D587 |  |  |
| **34** | ^RLC-NTE^K5 | ^Loop-3^N568 |  |  |
|  | ^RLC-NTE^K8 | ^L50-anchors Loop-3^D587 |  |  |
|  | ^RLC-NTE^R9 | ^Wedge^I585 |  |  |
|  | ^RLC-NTE^E22 | ^Loop-3^K570 |  |  |
| **36** | ^RLC-NTE^R9 | ^Wedge^I585, ^Wedge^A583 |  |  |
|  | ^RLC-NTE^K8 | ^L50-anchors Loop-3^D587 |  |  |
|  | ^RLC-NTE^E22 | ^Loop-3^K570 |  |  |
| **37** | ^RLC-NTE^K8 | ^L50-anchors Loop-3^D587 | ^RLC-NTE^K5 | ^NTE^D685 |
|  | ^RLC-NTE^R9 | ^Wedge^A583 | ^RLC-NTE^K7 | ^P-loop^E179 |
|  | ^RLC-NTE^S19 | ^Loop-3^K570 | ^RLC-NTE^K8 | ^SH3^E45 |
|  | ^RLC-NTE^F21 | ^Loop-3^K570 |  |  |
| **41** | ^RLC-NTE^K8 | ^L50-anchors Loop-3^D587 | ^RLC-NTE^K5 | ^NTE^D685 |
|  | ^RLC-NTE^S19 | ^Loop-3^K570 | ^RLC-NTE^K8 | ^SH3^E45 |
|  | ^RLC-NTE^F21 | ^Loop-3^K570 | ^RLC-NTE^F18 | ^Loop-3^K570 |
|  | ^RLC-NTE^Q23 | ^Loop-3^K570 | ^RLC-NTE^F21 | ^Loop-3^K570 |
| **44** | ^RLC-NTE^F18 | ^Loop-3^K570 | ^RLC-NTE^K5 | ^NTE^D685 |
|  | ^RLC-NTE^F21 | ^Loop-3^K570 | ^RLC-NTE^K8 | ^SH3^Q44,^SH3^E45 |
|  | ^RLC-NTE^Q23 | ^Loop-3^K570 | ^RLC-NTE^F18 | ^Loop-3^K570 |
|  |  |  | ^RLC-NTE^F21 | ^Loop-3^K570 |
| **47** | ^RLC-NTE^R9 | ^Switch-II^F468 | ^RLC-NTE^K5 | ^NTE^D685 |
|  | ^RLC-NTE^E22 | ^Loop-3^K570 | ^RLC-NTE^K8 | ^SH3^Q44,^SH3^E45 |
|  |  |  | ^RLC-NTE^F18 | ^Loop-3^K570 |
|  |  |  | ^RLC-NTE^S19 | ^Loop-3^R567 |
|  |  |  | ^RLC-NTE^F21 | ^Loop-3^K570 |
| **49** |  |  | ^RLC-NTE^K5 | ^NTE^D685 |
|  |  |  | ^RLC-NTE^K8 | ^SH3^D41, ^SH3^Q44,^SH3^E45 |
|  |  |  | ^RLC-NTE^F18 | ^Loop-3^K570 |
|  |  |  | ^RLC-NTE^S19 | ^Loop-3^R567 |
|  |  |  | ^RLC-NTE^F21 | ^Loop-3^K570 |
| **50** | ^RLC-NTE^R9 | ^Switch-II^F468, ^Switch-II^D469 |  |  |
|  | ^RLC-NTE^E22 | ^Loop-3^K570 |  |  |
| **51** | ^RLC-NTE^S19 | ^Loop-3^K572 | ^RLC-NTE^K5 | ^NTE^D685 |
|  | ^RLC-NTE^E22 | ^Loop-3^K570 | ^RLC-NTE^K8 | ^SH3^K43, ^SH3^E45 |
|  |  |  | ^RLC-NTE^F18 | ^Loop-3^K570 |
|  |  |  | ^RLC-NTE^S19 | ^Loop-3^R567 |
|  |  |  | ^RLC-NTE^F21 | ^Loop-3^K570 |
| **52** | ^RLC-NTE^K7 | ^Switch-II^D469 |  |  |
|  | ^RLC-NTE^R9 | ^Wedge^A583 |  |  |
|  | ^RLC-NTE^E22 | ^Loop-3^K570 |  |  |
| **56** | ^RLC-NTE^K7 | ^Switch-II^D469 | ^RLC-NTE^M1 | ^SH3^K43 |
|  |  | ^Wedge^I585 | ^RLC-NTE^K5 | ^NTE^D685 |
|  | ^RLC-NTE^E22 | ^Loop-3^K570 | ^RLC-NTE^K8 | ^SH3^K41, ^SH3^K43, ^SH3^E45 |
|  |  |  | ^RLC-NTE^E22 | ^Loop-3^K570 |
| **60** | ^RLC-NTE^A6 | ^Loop-3^R567 |  |  |
|  | ^RLC-NTE^K7 | ^Switch-II^D469 |  |  |
|  | ^RLC-NTE^E22 | ^Loop-3^K570 |  |  |
| **70** | ^RLC-NTE^K7 | ^Switch-II^D469 |  |  |
|  | ^RLC-NTE^R9 | ^Wedge^A583 |  |  |
|  | ^RLC-NTE^E22 | ^Loop-3^K570 |  |  |
| **76** | ^RLC-NTE^K7 | ^Loop-3^K570 |  |  |
|  | ^RLC-NTE^E22 | ^Loop-3^K570 |  |  |
| **78** |  |  | ^RLC-NTE^K5 | ^NTE^D685 |
|  |  |  | ^RLC-NTE^K8 | ^SH3^K41, ^SH3^K43, ^SH3^E45 |
|  |  |  | ^RLC-NTE^F18 | ^Loop-3^K570 |
|  |  |  | ^RLC-NTE^E22 | ^Loop-3^N568 |
| **82** | ^RLC-NTE^K7 | ^Switch-II^D469 | ^RLC-NTE^K5 | ^NTE^D685 |
|  | ^RLC-NTE^G11 | ^Switch-II^I467 | ^RLC-NTE^K8 | ^SH3^K41, ^SH3^K43, ^SH3^E45 |
|  | ^RLC-NTE^S19 | ^Loop-3^K570 | ^RLC-NTE^F18 | ^Loop-3^K570 |
|  | ^RLC-NTE^E22 | ^Loop-3^K570 | ^RLC-NTE^S19 | ^Loop-3^N568 |
|  |  |  | ^RLC-NTE^Q23 | ^Loop-3^K570 |
| **83** | ^RLC-NTE^K7 | ^Switch-II^D469 |  |  |
|  | ^RLC-NTE^G11 | ^Switch-II^I467 |  |  |
|  | ^RLC-NTE^S19 | ^Loop-3G^571 |  |  |
|  | ^RLC-NTE^E22 | ^Loop-3^K570 |  |  |
| **84** | ^RLC-NTE^K7 | ^Switch-II^D469 | ^RLC-NTE^K8 | ^SH3^K41, ^SH3^K43, ^SH3^E45 |
|  | ^RLC-NTE^S19 | ^Switch-II^D469, ^Loop-3^K570, ^Loop-3^K572 | ^RLC-NTE^F18 | ^Loop-3^K570 |
|  | ^RLC-NTE^E22 | ^Loop-3^K570 | ^RLC-NTE^F21 | ^Loop-3^K570 |
|  |  |  | ^RLC-NTE^E22 | ^Loop-3^K570 |
| **87** | ^RLC-NTE^K7 | ^Switch-II^D469, ^L50-anchors Loop-3^D587 |  |  |
|  | ^RLC-NTE^E22 | ^Loop-3^K570 |  |  |
| **90** | ^RLC-NTE^R9 | ^P-loop^E179 |  |  |
|  | ^RLC-NTE^K7 | ^L50-anchors Loop-3^D587 |  |  |
|  | ^RLC-NTE^E22 | ^Loop-3^K570 |  |  |
| **94** | ^RLC-NTE^R9 | ^P-loop^E179 |  |  |
|  | ^RLC-NTE^K7 | ^Switch-II^F468, ^Switch-II^D469 |  |  |
|  | ^RLC-NTE^S19 | ^Loop-3^K572 |  |  |
|  | ^RLC-NTE^E22 | ^Loop-3^K570 |  |  |
| **100** | ^RLC-NTE^K4 | ^Loop-3^R567 |  |  |
|  | ^RLC-NTE^K7 | ^Switch-II^D469 |  |  |
|  | ^RLC-NTE^R9 | ^P-loop^E179 |  |  |
|  | ^RLC-NTE^S19 | ^Loop-3^G571 |  |  |
| **101** | ^RLC-NTE^K4 | ^Loop-3^R567 |  |  |
|  | ^RLC-NTE^K5 | ^Loop-3^R567 |  |  |
|  | ^RLC-NTE^K7 | ^Switch-II^D469 |  |  |
|  | ^RLC-NTE^R9 | ^P-loop^E179 |  |  |
|  | ^RLC-NTE^E22 | ^Loop-3^K570 |  |  |
| **103** | ^RLC-NTE^K4 | ^Loop-3^R567 | ^RLC-NTE^K5 | ^NTE^D685 |
|  | ^RLC-NTE^K7 | ^Switch-II^F468, ^Switch-II^D469 | ^RLC-NTE^K8 | ^SH3^K41, ^SH3^K43, ^SH3^E45 |
|  | ^RLC-NTE^R9 | ^P-loop^E179 | ^RLC-NTE^N16 | ^Loop-3^R567 |
|  | ^RLC-NTE^S19 | ^Loop-3^K570 | ^RLC-NTE^F18 | ^Loop-3^K570 |
|  | ^RLC-NTE^E22 | ^Loop-3^K570 | ^RLC-NTE^S19 | ^Loop-3^ R567, ^Loop-3^N568, ^Loop-3^K570 |
|  |  |  | ^RLC-NTE^F21 | ^Loop-3^K570 |
|  |  |  | ^RLC-NTE^E22 | ^Loop-3^K570 |
| **104** | ^RLC-NTE^K4 | ^Loop-3^R567 | ^RLC-NTE^K5 | ^NTE^D685 |
|  | ^RLC-NTE^K7 | ^Switch-II^D469 | ^RLC-NTE^K8 | ^SH3^K41, ^SH3^K43, ^SH3^E45 |
|  | ^RLC-NTE^R9 | ^P-loop^E179 | ^RLC-NTE^F18 | ^Loop-3^K570 |
|  | ^RLC-NTE^N16 | ^Loop-3^G571 | ^RLC-NTE^S19 | ^Loop-3^ R567 |
|  | ^RLC-NTE^S19 | ^Loop-3^K570 | ^RLC-NTE^F21 | ^Loop-3^K570 |
|  | ^RLC-NTE^E22 | ^Loop-3^K570 |  |  |
| **105** |  |  | ^RLC-NTE^K5 | ^SH3^Q44, ^NTE^D685 |
|  |  |  | ^RLC-NTE^K8 | ^SH3^K41, ^SH3^K43, ^SH3^E45 |
|  |  |  | ^RLC-NTE^N16 | ^Loop-3^R567 |
|  |  |  | ^RLC-NTE^S19 | ^Loop-3^ R567 |
|  |  |  | ^RLC-NTE^F21 | ^Loop-3^K570 |
| **108** | ^RLC-NTE^A6 | ^near Loop-3^I585 | ^RLC-NTE^K5 | ^NTE^D685 |
|  | ^RLC-NTE^K7 | ^Switch-II^D469 | ^RLC-NTE^K8 | ^SH3^K41, ^SH3^Q44, ^SH3^E45 |
|  | ^RLC-NTE^R9 | ^P-loop^E179 |  |  |
|  | ^RLC-NTE^S19 | ^Loop-3^G571, ^Loop-3^K572 |  |  |
|  | ^RLC-NTE^E22 | ^Loop-3^K570 |  |  |
| **111** | ^RLC-NTE^A6 | ^near Loop-3^I585 |  |  |
|  | ^RLC-NTE^K7 | ^near Loop-3^I585, ^L50-anchors Loop-3^D587 |  |  |
|  | ^RLC-NTE^R9 | ^P-loop^E179 |  |  |
|  | ^RLC-NTE^S19 | ^Loop-3^G571 |  |  |
|  | ^RLC-NTE^E22 | ^Loop-3^K570 |  |  |
| **118** | ^RLC-NTE^A6 | ^near Loop-3^I585 | ^RLC-NTE^K5 | ^NTE^D685 |
|  | ^RLC-NTE^R9 | ^P-loop^E179 | ^RLC-NTE^K8 | ^SH3^K41, ^SH3^Q44, ^SH3^E45 |
|  | ^RLC-NTE^N16 | ^Loop-3^G571 | ^RLC-NTE^Q23 | ^Loop-3^N568 |
|  | ^RLC-NTE^S19 | ^Loop-3^K570 |  |  |
|  | ^RLC-NTE^E22 | ^Loop-3^K570 |  |  |
| **119** | ^RLC-NTE^K5 | Q564 | ^RLC-NTE^K5 | ^NTE^D685 |
|  | ^RLC-NTE^A6 | ^near Loop-3^I585 | ^RLC-NTE^K8 | ^SH3^K41, ^SH3^Q44, ^SH3^E45 |
|  | ^RLC-NTE^R9 | ^P-loop^E179 | ^RLC-NTE^S19 | ^Loop-3^ K565 |
|  | ^RLC-NTE^S19 | ^Loop-3^K570 | ^RLC-NTE^Q23 | ^Loop-3^N568 |
|  | ^RLC-NTE^E22 | ^Loop-3^K570 |  |  |
| **123** | ^RLC-NTE^A6 | ^near Loop-3^I585 | ^RLC-NTE^K5 | ^NTE^D685 |
|  | ^RLC-NTE^K7 | ^near Loop-3^I585, ^L50-anchors Loop-3^D587 | ^RLC-NTE^K8 | ^SH3^K41, ^SH3^Q44, ^SH3^E45 |
|  | ^RLC-NTE^K8 | ^L50-anchors Loop-3^Y582 | ^RLC-NTE^E22 | ^Loop-3^ K565 |
|  | ^RLC-NTE^R9 | ^P-loop^E179 |  |  |
|  | ^RLC-NTE^S19 | ^Loop-3^G571 |  |  |
|  | ^RLC-NTE^E22 | ^Loop-3^K570 |  |  |
