# Supplementary material for: Dynamics of the β-cardiac myosin auto-inhibited state explain cardiomyopathy pathogenesis
Source: Nat Commun. 2026 Jun 4;17:5502. doi: 10.1038/s41467-026-73572-5 (PMC13287786; doi:10.1038/s41467-026-73572-5)
Supplement: Supplementary file 22 — Reporting Summary [file 41467_2026_73572_MOESM22_ESM.pdf]

nature portfolio

Corresponding author(s): Anne Houdusse

Last updated by author(s): Apr 29, 2026

Reporting Summary

Nature Portfolio wishes to improve the reproducibility of the work that we publish. This form provides structure for consistency and transparency in reporting. For further information on Nature Portfolio policies, see our Editorial Policies and the Editorial Policy Checklist.

Statistics

For all statistical analyses, confirm that the following items are present in the figure legend, table legend, main text, or Methods section.

n/a	Confirmed	
☐	☒	The exact sample size (*n*) for each experimental group/condition, given as a discrete number and unit of measurement
☐	☒	A statement on whether measurements were taken from distinct samples or whether the same sample was measured repeatedly
☒	☐	The statistical test(s) used AND whether they are one- or two-sided<br>*Only common tests should be described solely by name; describe more complex techniques in the Methods section.*
☒	☐	A description of all covariates tested
☒	☐	A description of any assumptions or corrections, such as tests of normality and adjustment for multiple comparisons
☐	☒	A full description of the statistical parameters including central tendency (e.g. means) or other basic estimates (e.g. regression coefficient) AND variation (e.g. standard deviation) or associated estimates of uncertainty (e.g. confidence intervals)
☒	☐	For null hypothesis testing, the test statistic (e.g. *F*, *t*, *r*) with confidence intervals, effect sizes, degrees of freedom and *P* value noted<br>*Give P values as exact values whenever suitable.*
☒	☐	For Bayesian analysis, information on the choice of priors and Markov chain Monte Carlo settings
☒	☐	For hierarchical and complex designs, identification of the appropriate level for tests and full reporting of outcomes
☒	☐	Estimates of effect sizes (e.g. Cohen's *d*, Pearson's *r*), indicating how they were calculated

Our web collection on statistics for biologists contains articles on many of the points above.

Software and code

Policy information about availability of computer code

Data collection	-CryoEM: the acquisition was supervised by the software EPU (ThermoFisher, version 3.14).<br><br>-Molecular dynamics: flexible regions of the models were rebuilt with Modeller (version 10.1).<br><br>-Molecular dynamics simulations were performed with GROMACS (v. 2023.3). CHARMM36m forcefield was used (software CHARMM-GUI, version 3.8).
Data analysis	-CryoEM: data processing was performed with CryoSPARC (version 4.7.1).<br>-Structure analysis: manual refinement and analysis were performed with Coot (version 0.9.8.96), Chimera (version 1.19), ChimeraX (version 1.10.1).<br>-Refinement: real space refinement was performed with the "real space refinement" module from Phenix (version 1.19.2-4158).<br><br>-Molecular dynamics: trajectories were generated, computed and analyzed with GROMACS (v. 2023.3) and VMD (version 1.8.4a53, June 29, 2021). Pymol was used to display and look at the trajectories (version 3.1.6.1).

For manuscripts utilizing custom algorithms or software that are central to the research but not yet described in published literature, software must be made available to editors and reviewers. We strongly encourage code deposition in a community repository (e.g. GitHub). See the Nature Portfolio guidelines for submitting code & software for further information.

Data

Policy information about availability of data

All manuscripts must include a data availability statement. This statement should provide the following information, where applicable:

- Accession codes, unique identifiers, or web links for publicly available datasets
- A description of any restrictions on data availability
- For clinical datasets or third party data, please ensure that the statement adheres to our policy

The Data Availability statement is included in the manuscript as follows:

"The atomic model generated in this study have been deposited in the PDB59 under accession codes: 9TLP [https://doi.org/10.2210/pdb9TLP/pdb] (WTConfA); 9TPK [https://doi.org/10.2210/pdb9TPK/pdb] (WTConfB); 9TPJ [https://doi.org/10.2210/pdb9TPJ/pdb] (E525K). The cryoEM map of of WTConfA, WTConfB, E525K have been deposited in the EMDB database60 under the accession numbers EMD-56108 [https://www.ebi.ac.uk/pdbe/entry/emdb/EMD-56108] (WTConfA); EMD-56107 [https://www.ebi.ac.uk/pdbe/entry/emdb/EMD-56107] (WTConfB); EMD-56106 [https://www.ebi.ac.uk/pdbe/entry/emdb/EMD-56106] (E525K), respectively.

The CryoEM dataset collected on E525K is available at the ESRF as Lannes, L. (2027). CryoEM-BAG France: Structural Biology using cryoEM in France [Dataset]. European Synchrotron Radiation Facility under doi.org/10.15151/ESRF-ES-1521652352.

The source data underlying Figure 2H, Supplementary Figure 9A-B, Supplementary Figure 15 A-F, and Supplementary Figure 16A-F are provided as Source Data file."

Research involving human participants, their data, or biological material

Policy information about studies with human participants or human data. See also policy information about sex, gender (identity/presentation), and sexual orientation and race, ethnicity and racism.

Reporting on sex and gender	n/a
Reporting on race, ethnicity, or other socially relevant groupings	n/a
Population characteristics	n/a
Recruitment	n/a
Ethics oversight	n/a

Note that full information on the approval of the study protocol must also be provided in the manuscript.

Field-specific reporting

Please select the one below that is the best fit for your research. If you are not sure, read the appropriate sections before making your selection.

[x] Life sciences [ ] Behavioural & social sciences [ ] Ecological, evolutionary & environmental sciences

For a reference copy of the document with all sections, see nature.com/documents/nr-reporting-summary-flat.pdf

Life sciences study design

All studies must disclose on these points even when the disclosure is negative.

Sample size	Molecular dynamics simulations were performed twice in each condition to ensure the reproducibility of the analysis.
Data exclusions	No data was excluded.

Replication	Two molecular dynamics simulations were performed for each condition. The sample size was determined based on the available computational resources and prior studies. Experimental data confirm that the major conclusions drawn from the two replicates can be observed in cryo-EM data of isolated cardiac IHM WT and E525K, as well as in cryo-ET of the unstabilized relaxed thick filament (see Chen et al., 2024, https://doi.org/10.1073/pnas.2311883121). Two simulations were therefore sufficient to demonstrate convergence and reproducibility of the phenomena discussed in this manuscript.<br><br>Each replicate was initiated independently. Data from the replicates are provided in Supplementary Data 1–3.<br><br>For each replicate, RMSD, RMSF, and inter-residue distances within the coiled-coil region were calculated and compared. All major findings were consistently reproduced.
Randomization	Samples were not randomized, as randomization is not applicable to molecular dynamics simulations. However, all replicates were conducted independently.
Blinding	Samples were not blinded, as blinding is not applicable to molecular dynamics simulations. However, all replicates were conducted independently.

Reporting for specific materials, systems and methods

We require information from authors about some types of materials, experimental systems and methods used in many studies. Here, indicate whether each material, system or method listed is relevant to your study. If you are not sure if a list item applies to your research, read the appropriate section before selecting a response.

Materials & experimental systems

n/a	Involved in the study
☒	☐ Antibodies
☐	☒ Eukaryotic cell lines
☒	☐ Palaeontology and archaeology
☒	☐ Animals and other organisms
☒	☐ Clinical data
☒	☐ Dual use research of concern
☒	☐ Plants

Methods

n/a	Involved in the study
☒	☐ ChIP-seq
☒	☐ Flow cytometry
☒	☐ MRI-based neuroimaging

Eukaryotic cell lines

Policy information about cell lines and Sex and Gender in Research

Cell line source(s)	HEK 293 and C2C12 were both from ATCC.
Authentication	Neither cell line was authenticated as they are both used only for reagent generation and not for cell biological studies. The HEK 293 cells are used only to make adenovirus. The C2C12 cells underwent appropriate differentiation upon starvation and produced the functional recombinant myosin proteins that were used in this study.
Mycoplasma contamination	Cells were not routinely tested for mycoplasma contamination.
Commonly misidentified lines (See ICLAC register)	n/a

Plants

Seed stocks	n/a
Novel plant genotypes	n/a
Authentication	n/a
