# Supplementary material for: Dynamics of the β-cardiac myosin auto-inhibited state explain cardiomyopathy pathogenesis
Source: Nat Commun. 2026 Jun 4;17:5502. doi: 10.1038/s41467-026-73572-5 (PMC13287786; doi:10.1038/s41467-026-73572-5)
Supplement: Supplementary file 23 — Transparent Peer Review File [file 41467_2026_73572_MOESM23_ESM.pdf]

nature portfolio

Peer Review File

Dynamics of the β-cardiac myosin auto-inhibited state explain cardiomyopathy pathogenesis

Corresponding Author: Dr Anne Houdusse

This file contains all reviewer reports in order by version, followed by all author rebuttals in order by version.

Version 0:

Reviewer comments:

Reviewer #1

(Remarks to the Author)
The manuscript by Auguin et al is a very robust and influential study that uses high-resolution cryo-EM structures of cardiac IHMs to model the entire thick filament assembly. It has several important components that make it well-suited for publication in Nature Communications:
1.I commend the authors for demonstrating the dynamic nature of the IHM structure. For some time, there was a perception in the field that proteins interact only in a single mode. In the manuscript, the authors not only show that structural dynamics exist but also beautifully demonstrate, using in silico methods, its importance for the WT and for cases where residues are mutated. The resultant conclusion that the naturally transient dynamic interactions, precisely tuned by the S2 sequence, govern the thick filament transition between the ON and OFF states is highly valuable.
2.The authors also demonstrate the advantage of the usage of the atomic models built using high-resolution density maps for the molecular dynamics simulations. Many published manuscripts contained inaccurate conclusions because they used atomic models derived from low-resolution data to extract nonexistent information. This manuscript nicely shows the power of the in silico approach when the starting model is correct.

I have a couple of minor comments/questions:
1.May the authors check the uploaded Movie 5, which cannot be played?
2.IHM ConfB does not fit into the ordered crowns 1 or 3. As the authors mention, the disordered Crown 2 may be adapting the ConfB conformation, which naturally causes its disordering. Is it possible to model the ConfB state in Crown2 within the thick filament model to obtain a complete structure for further analysis and MDS?
3.It was a bit confusing to me how the refinement of the thick filament model based on the high-resolution data was done against the 6A map… Should it cause a loss of high-resolution details? Did the authors use constraints to prevent it from happening?

Reviewer #2

(Remarks to the Author)
The paper by Auguin et al describes a combined CryoEM/MD investigation of the Interacting Head Motif in cardiac myosin. Compared to previous studies, this work uses higher resolution structural data to shed light on the dynamics of this motif in WT and mutants related to disease.

The paper is very well written and beautiful supplementary movies are provided, which clarify the nature of the conformational changes observed.

While the findings are novel and potentially very interesting, the following points should be addressed before publication:

• Conclusions are drawn from individual simulations for each system (WT and mutants), established practice for unbiased MD is now to run simulations multiple times and report statistics to reassure that results are reproducible. All the comments on predictive power of MD based on current number of simulations are largely overstated and need to be backed by replicas.
• From the atomistic point of view, it is unclear why E525K and E903K would lead to a different behaviour: can the reader be guided more on the reason of this difference? Is it due to the different environment around the two residues?

• Transient interactions are repeatedly mentioned in the paper, their time evolution along the simulations should be shown.
• Unless we missed it somewhere else, the methods section contains very few details about how MD simulations were carried out. How was the protonation state of ionisable residues at pH7 determined? Were restraints applied during minimization and equilibration, how and on which atoms? What barostat and thermostat were used? What protocol was used exactly for equilibration and how long was it run for? Time step? Integration method? Shape of the box? Minimal distance between protein and box? Reporting only the size of the box is not enough. Were PBC applied? Sampling frequency of frames for analyses?
• More details need also to be included on how the modelling of missing residues was done, in particular of those involved in the interactions discussed in the paper. Was homology modelling used at any stage? If yes, how? How was the potential impact of alternative modelling strategies on the results assessed?

Minor points:

• vdW contacts and other types of interactions are often mentioned but unclear how they were determined and often difficult to appreciate from images, more quantitative analyses should be carried out over the whole trajectory rather than just some individual frames (plots of time evolution of interactions, occupancy etc)
- The flexibility difference between WT and E525K shown via sausage plots and colour gradient in Supplementary Figure 9 is not very evident (the two images look very similar in terms of flexibility), other types of representations might be more suitable. This image is used in the text to show how MD results agree with CryoEM ones ("The flexibility observed for each residue in the simulations (Supplemental Fig. 9B-D) is consistent with the CryoEM data (Supplemental Fig. 7B, Movie 1)"), but at the moment this is not really shown: the mentioned CryoEM data are about flexibility of FH, which is not clearly shown in Supplementary Figure 9.
- The following statement is unclear: "The importance of the asymmetric S2 bend (Cluster 0) in the stabilization of a unique asymmetric CarIHM structure is highlighted by the number of missense mutations that can result in cardiomyopathies (Fig. 4C, Supplemental Fig. 12)." The mutations shown seem to be evenly distributed throughout the coiled coil, what information is this giving us? Is this distribution any different from the one observed in the motor domains for example? How can a link be drawn between the distribution shown and the importance of the asymmetric bend?
- "Interestingly, the HCM MYH7 E45 mutations to Asp, Gly, or Lys, classified as variants of unknown significance (VUS) for HCM, belong to this category (to date, no mutations of E45 are officially ascertained as pathogenic/likely pathogenic). Our study indicates that this residue is critical for the formation of the intercrown contacts (Movie 14)." Residue E45 does not seem to be shown in the movie.

Reviewer #3

(Remarks to the Author)
I co-reviewed this manuscript with one of the reviewers who provided the listed reports. This is part of the Nature Communications initiative to facilitate training in peer review and to provide appropriate recognition for Early Career Researchers who co-review manuscripts.

Version 1:

Reviewer comments:

Reviewer #1

(Remarks to the Author)
The authors addressed all my points, and I can recommend the paper for publication in its current form.

Reviewer #2

(Remarks to the Author)
We appreciate the effort made to address all comments. Few minor remaining points:

* Movie 5 still cannot be played
* "A transient interaction between FHK903 and BH 291 E653 occurs, but S2 continues to explore positions far from 292 those of WT-CarIHM. The orientation and flexibility of the proximal S2, below the Hook and prior to 293 Cluster 1, are also significantly affected (Supplementary Fig. 9D)." Probably Supplementary Fig. 9E (not D) should be mentioned at the end of the sentence
* Ideally reproducibility across replicas should be confirmed for all the main findings.

Open Access This Peer Review File is licensed under a Creative Commons Attribution 4.0 International License, which permits use, sharing, adaptation, distribution and reproduction in any medium or format, as long as you give appropriate credit to the original author(s) and the source, provide a link to the Creative Commons license, and indicate if changes were made.
In cases where reviewers are anonymous, credit should be given to 'Anonymous Referee' and the source.
The images or other third party material in this Peer Review File are included in the article's Creative Commons license, unless indicated otherwise in a credit line to the material. If material is not included in the article's Creative Commons license and your intended use is not permitted by statutory regulation or exceeds the permitted use, you will need to obtain permission directly from the copyright holder.
To view a copy of this license, visit https://creativecommons.org/licenses/by/4.0/

Reviewer #1 Comments:

The manuscript by Auguin et al is a very robust and influential study that uses high-resolution cryo-EM structures of cardiac IHMs to model the entire thick filament assembly. It has several important components that make it well-suited for publication in Nature Communications:

1.I commend the authors for demonstrating the dynamic nature of the IHM structure. For some time, there was a perception in the field that proteins interact only in a single mode. In the manuscript, the authors not only show that structural dynamics exist but also beautifully demonstrate, using in silico methods, its importance for the WT and for cases where residues are mutated. The resultant conclusion that the naturally transient dynamic interactions, precisely tuned by the S2 sequence, govern the thick filament transition between the ON and OFF states is highly valuable.

2.The authors also demonstrate the advantage of the usage of the atomic models built using high-resolution density maps for the molecular dynamics simulations. Many published manuscripts contained inaccurate conclusions because they used atomic models derived from low-resolution data to extract nonexistent information. This manuscript nicely shows the power of the in silico approach when the starting model is correct.

We thank Reviewer #1 for the positive evaluation of our work.

I have a couple of minor comments/questions:
1.May the authors check the uploaded Movie 5, which cannot be played?

This issue was resolved by uploading a re-encoded version of Movie 5 that should display properly in all platforms.

2.IHM ConfB does not fit into the ordered crowns 1 or 3. As the authors mention, the disordered Crown 2 may be adapting the ConfB conformation, which naturally causes its disordering. Is it possible to model the ConfB state in Crown2 within the thick filament model to obtain a complete structure for further analysis and MDS?

Crown 2 appears to be capable of adopting multiple orientations in all the available structures of the cardiac relaxed filament, whether stabilized by Mavacamten (Dutta *et al.*, 2023; Tamborrini et al., 2023) or not (Chen *et al.*, 2024). Modeling Crown 2 as ConfB or ConfA would be theoretically possible, however, the resolution of the cryoEM

map is not sufficient to choose a particular IHM conformation. In addition, it would substantially increase the number of atoms. We have already reached ~ 4.6 million which is close to the limit of CHARMM rebuilding capacity. It would also require a larger solvated box and significantly increased computational time. Moreover, the 140 ns simulations would likely not allow to observe the motif unfolding, we would at most see Crown 2 appearing more dynamic, an observation already supported by existing EM studies. While such an investigation would be valuable for future work, it would require additional resources and falls beyond the scope of the present study, which focuses on the more stabilized heads (Crowns 1 and 3).

3.It was a bit confusing to me how the refinement of the thick filament model based on the high-resolution data was done against the 6A map... Should it cause a loss of high-resolution details? Did the authors use constraints to prevent it from happening?

Biasing the high-resolution model with the 6 Å map during refinement could indeed introduce errors, so we optimized our methodology to minimize its impact. We used real-space refinement to refine our model against the map (Alfonine *et al*., 2018), avoiding the tasks that could substantially alter the model and reduce the benefits of high-resolution details such as simulated annealing, side-chain optimizations, and morphing. In our studies, we only applied the gradient-driven minimization with secondary structure restraints. Between real-space refinement cycles, we performed *in silico* minimization on the side-chains (which are not visible in the medium/low-resolution map), while keeping the Cα stable.

From our experience, when the high-resolution model is of good quality, it does not deviate significantly from the initial structure. Moreover, our procedure consistently improved both the correlation coefficient and the geometry of the models in previous studies (see Robert-Paganin *et al*., 2021 for examples), while also providing realistic interfaces from the chemistry point of view. Interestingly, the interfaces stabilizing the cardiac IHM in the isolated motif closely resemble those observed in the refined relaxed filament (see **Supplementary Tables 3 & 4**). Thus, our approach takes advantage of both the accuracy of high-resolution data and the contextual information provided by the filament at medium- to low-resolution.

Details were added in the method section for the methodology used, as requested by Reviewer #2.

Reviewer #2 (Remarks to the Author):

The paper by Auguin et al describes a combined CryoEM/MD investigation of the Interacting Head Motif in cardiac myosin. Compared to previous studies, this work uses

higher resolution structural data to shed light on the dynamics of this motif in WT and mutants related to disease.

The paper is very well written and beautiful supplementary movies are provided, which clarify the nature of the conformational changes observed.

While the findings are novel and potentially very interesting, the following points should be addressed before publication:

We thank Reviewer #2 for the positive appreciation of our work. The reviewer provided very helpful comments that, in our opinion, substantially improved the quality of the manuscript. Several changes have been introduced in the revised version, particularly to improve the molecular dynamics section.

• Conclusions are drawn from individual simulations for each system (WT and mutants), established practice for unbiased MD is now to run simulations multiple times and report statistics to reassure that results are reproducible. All the comments on predictive power of MD based on current number of simulations are largely overstated and need to be backed by replicas.

Molecular dynamics simulations were performed at least twice for each condition to ensure the reproducibility of the phenomena discussed in the manuscript. We have now added this fact in the text. All simulations, whether performed on the isolated $_{Car}$IHM or on the filament, led to the same conclusions. We have included in the manuscript the analysis of the duplicate filament simulation (see **Supplementary Fig. 16**). Raw data files containing the RMSD data for the duplicate isolated $_{Car}$IHM simulations will be provided for online availability.

(p. 17, l. 537-538) “The simulations were performed twice, revealing that these patterns are conserved in the second run (**Supplementary Fig. 16**)”

• From the atomistic point of view, it is unclear why E525K and E903K would lead to a different behaviour: can the reader be guided more on the reason of this difference? Is it due to the different environment around the two residues?

We agree with Reviewer #2 that this information was lacking. We therefore added some additional figures (**Supplementary Fig. 9F-H**) showing how the two mutations differentially alter the local charge network, thus exerting opposite effects on the stabilization of the S2 coiled-coil. The corresponding paragraph in the manuscript has also been revised and now reads as follows (changes highlighted in green):

New text (p. 8, l. 269-298)

In detail, the *in silico* and CryoEM results are also consistent and complementary. The flexibility observed for each residue in the MD simulations (**Supplementary Fig. 9C-E**) matches the conformational variability captured by CryoEM data (**Supplementary Fig. 7B**, **Supplementary Movie 1**). The MD simulations further allow visualization of the specific interactions that can occur at the $_{Car}$IHM interfaces over time (**Supplementary Movies 5 & 6**). In particular, the interactions established between ^{FH}Loop-2 and the S2 illustrate perfectly the concept of "musical chairs": the flexible ^{FH}Loop-2 forms a dynamic interface through a network of transient polar interactions. The transient nature of these interactions, combined with the flexibility of ^{FH}Loop-2, makes it possible to maintain the global interface despite changes in the orientation of S2 (**Supplementary Movie 7**). In the **WT** simulation, the S2 explores multiple positions within Cluster 2 forming only a few H-bonds (**Supplementary Movies 5 & 6**), which is consistent with the CryoEM results (**Supplementary Movie 1**). While E525 is involved in electrostatic interactions with K484 and N656, residues ^{S2}D900 and ^{S2}E903 remain as free negative charges contributing to the flexibility of the S2 (**Supplementary Figure 9F**). In the **E525K** simulation, K525 rapidly forms an interaction with ^{S2}E903 promoting the formation of specific bonds between BH and S2 in both Cluster 1 and Cluster 2 (**Supplementary Movie 6**), as observed in CryoEM maps. K525 introduces a positive charge that enables interactions with both ^{S2}D900 and ^{S2}E903, significantly reducing the flexibility of the S2 that remains in position (**Fig. 2I**, **Supplementary Fig. 9B, 9E, 9G**). In contrast, when the **E903K** mutation is introduced, major differences appear in both clusters, resulting in a modification of all contacts at the BH/S2 interface (**Fig. 2I**, **Supplementary Movie 6**). In Cluster 2, S2 deviates in less than 40 ns from its initial position due to a lack of stabilization (**Fig. 2I**, **Supplementary Movies 5 & 6**). Although K903 introduces a positive charge on the S2, it cannot interact with E525 that is already involved in interactions with K484 and N656 (**Supplementary Fig. 9H**). A transient interaction between ^{FH}K903 and ^{BH}E653 occurs, but S2 continues to explore positions far from those of WT-$_{Car}$IHM. The orientation and flexibility of the proximal S2, below the Hook and prior to Cluster 1, are also significantly affected (**Supplementary Fig. 9D**). This single E903K mutation results in a drastic disruption of the conformational ensembles explored by WT, which explains the destabilization of $_{Car}$IHM. In contrast, E525K strongly biases the conformational ensemble to enrich it in conformations close to ^{WT}ConfA. Our analysis shows how two mutations within the same region alter the local electrostatic network (**Supplementary Fig. 9F-H**) resulting in opposite effects on the stabilization of the S2 (**Supplementary Movie 3, 5 & 6**).

• Transient interactions are repeatedly mentioned in the paper, their time evolution along the simulations should be shown.

Transient interactions are mentioned in the manuscript since the nature of the $_{Car}$IHM interface indicates that the interactions are not stable but transient, making all these stabilizing interfaces quite dynamic. Constant change of partners is observed in a nanosecond time-scale. Since these interactions are formed and are lost in a random manner, quantifying precisely their evolution in time becomes less insightful than analyzing the persistence of the interface. We compared the distinct simulations for a particular IHM and can indicate that similar interactions occur at these interfaces in different molecular dynamic simulations. To clarify this point, we have added a Supplementary Movie showing the evolution of the ^{FH}Loop-2/S2 interface (**Supplementary Movie 7**), illustrating how these transient interactions change throughout the WT-ConfA simulation. This addition helps to better convey the "musical

chairs” concept. The corresponding section of the manuscript has been revised accordingly, with the following addition:

Text (p.8, l. 273-277). “In particular, the interactions established between ^{FH}Loop-2 and the S2 illustrate perfectly the concept of “musical chairs”: the flexible ^{FH}Loop-2 forms a dynamic interface through a network of transient polar interactions. The transient nature of these interactions, combined with the flexibility of FHLoop-2, makes it possible to maintain the global interface despite changes in the orientation of S2 (**Supplementary Movie 7**).“

This observation is also illustrated in our analysis of the interactions observed during the molecular dynamics simulation starting from the ConfA or ConfB $_{car}$IHM (see **Supplementary Table 3**), as well as in the analysis of interactions within the filament observed during the simulations (**Supplementary Movie 3** for the S2 of $_{Car}$IHM and **Supplementary Movie 10** for the human cardiac relaxed filament). It is further demonstrated in **Supplementary Movies 13**, **14**, and **15**, in which residue names are displayed.

To illustrate this point for the molecular dynamic simulations of the thick filament, the interactions involving the intercrown for two independent dynamic simulations are compared in **Supplementary dataset1**. Note that the result is similar even though the intrinsically disordered residues of the intercrown were modeled at first in distinct initial positions (or coordinates)**.**

While both dynamics differ in detail for the timing into which the interactions occur, the position of the intercrown, its dynamic nature are similar and the position of the intercrown and consequence on the positioning of S15 are similar.

• Unless we missed it somewhere else, the methods section contains very few details about how MD simulations were carried out. How was the protonation state of ionisable residues at pH7 determined? Were restraints applied during minimization and equilibration, how and on which atoms? What barostat and thermostat were used? What protocol was used exactly for equilibration and how long was it run for? Time step? Integration method? Shape of the box? Minimal distance between protein and box? Reporting only the size of the box is not enough. Were PBC applied? Sampling frequency of frames for analyses?

PBC were indeed applied in our simulations. Due to size limitations in the manuscript, we chose to reduce the details in the manuscript, but we agree with Reviewer #2 that this information is needed. The method section has been revised in consequence, including more details.

(p. 24, l. 827-831). “General parameters used for all-atom molecular dynamics

For all the molecular dynamics experiments performed in this work, 500 ps NVT equilibration runs were performed (time step 0.001 ps with the integrator “md”) with GROMACS

(version 2023.3)[48]. Restrains were set for backbone and side chains atoms to 400 kJ.mol^{-1}.nm^{-2} and 40 kJ.mol^{-1}.nm^{-2} respectively. Hydrogen bonds were constrained using LINCS algorithm."

We added the barostat and thermostat:

(p.24, l. 844-847). "The simulations were performed using constant pressure and temperature ensemble (NPT). The temperature and the pressure of the system were fixed at 310.15 K with the Nosé-Hoover thermostat and 1 bar with the Parrinello-Rahman barostat[53,54]. The MD production time step was set to 2fs (dt = 0.002ps) for them all. WT-ConfA, E525K and E903K systems contain 1,242,557; 1,258,385 and 1,226,383 atoms respectively. The simulations were performed over 135 ns with GROMACS (version 2023.3)[48]."

The section describing the molecular dynamics simulations are more complete:

(p. 24, l. 833-843) "Three conditions were examined in all-atom MD simulations: **WT-ConfA** that started from the coordinates of the CryoEM structure ^{WT}ConfA; **E525K** (E525K-ConfA) in which the side chain of residue E525 was manually changed for a lysine; and **E903K** (E903K-ConfA) in which the side chain of residue E903 was manually changed for a lysine. Each simulation was performed twice, to ensure the reproducibility of the observations. The models were built and optimized using all-atom systems on CHARMM36m forcefields[51]. All the models were built in a box consisting of a cube with an edge 232 Å. The shortest periodic distance between the protein and the box was 5.1 nm at 0 ns. All simulations were performed in the same condition (150 mM KCl, pH 7.5 with one protonation of the histidine residue, system HSD) and contained the $_{Car}$IHM model and TIP3 explicit water molecules. Long-range interactions were computed using the particle mesh Ewald (PME) method[53], thus facilitating the calculation of the energies".

(p. 25, l. 883-896) All-atom molecular dynamics of the cardiac relaxed filament

The starting point for the all-atom MD simulations performed on the filament was based on the atomic coordinates of the relaxed filament. The model was built and optimized using CHARMM36m forcefield (software CHARMM-GUI)[49–51]. The system was built using a box consisting of a cube with an edge of 365 Å and a volume of 365 Å^3. The shortest periodic distance between the protein and the box was 2.17 nm at 0 ns. It contained all atoms of the thick filament, TIP3 waters with 150 mM KCl, pH 7.5 and one single protonation of the histidine (HSD form). Thus, the long-term simulation was performed using 4,613,271 atoms, 310.15 K of temperature and 1 bar of pressure. The simulation was performed with GROMACS (version 2023.3)[48] for 140 ns. During minimization, to maintain the overall structure of the filament, constraints of 40 kJ.mol^{-1}.nm^{-2} were kept maintaining the Cα of MyBP-C, Titin and the coiled-coils constituting the core of the filament. Crowns 1 and 3, including the heavy chains, the ELCs and RLCs, were free of constraints. The simulations were performed twice to ensure the reproducibility of the results, with the "flexible interfaces" (loop 686-711 from the C5 domain of MyBP-C; myosin Loop-1 (residues 200-215) or the N-terminal extension of the RLC) rebuilt independently in the duplicate (**Supplementary Fig. 16**).

Added in Trajectory analysis:

(p. 25, l. 898-899) "Frames of 0.1 ns steps were extracted from the original recorded simulations (sampled at 10ps) for the analysis."

• More details need also to be included on how the modelling of missing residues was done, in particular of those involved in the interactions discussed in the paper. Was homology modelling used at any stage? If yes, how? How was the potential impact of alternative modelling strategies on the results assessed?

We agree with Reviewer #2 that this information was missing. We also acknowledge that modeling loops remains challenging, as even AlphaFold2 and RoseTTAFold are considered inadequate for accurately modeling flexible regions (see Wang *et al.*, 2024, briefing in bioinformatics). In our case, we used a hybrid approach combining semi-manual building of the loops and molecular dynamics to optimize the geometry and interfaces. The procedure was added in methods:

(p.24-25, l. 857-865) "The missing structural elements such as the loop formed by residues 686-711 from the C5 domain of MyBP-C, myosin Loop-1 (residues 200-215) or the N-terminal extension of the RLC (residues 1-23) were rebuilt with Modeller (v 10.1)[56] provided with Chimera. The system was built and optimized with QuickMD simulator from CHARMM-GUI[49,50]. An initial step of 1 ns simulation was performed, the model was relaxed in a box consisting with a cube of an edge of 365 Å and a volume of 365 Å^3, with explicit water (TIP3P) with the CHARMM36m force field[51], salt (150 mM KCl, pH 7.5). The shortest periodic distance between the protein and the box was 2.17 nm at 0 ns. During this run, the backbone of the filament was restrained, keeping the Cα of the entire model maintained with constrains of 400 kJ.mol^{-1}.nm^{-2}, except for the flexible interfaces that were free. This allowed computing of realistic interfaces in these regions that were not rebuilt in the initial model 8G4L[24], although they are part of the interfaces."

Flexible loops in the filament were remodeled independently before each step of molecular dynamics and consistently resulted in similar interactions. For instance, the N-terminal extension of the RLC established comparable interactions in both simulations (see **Supplementary Fig. 16G-I**). This approach helps mitigate potential bias arising from the initial positioning of flexible interfaces that were not resolved in the cryo-EM map.

Minor points:

• vdW contacts and other types of interactions are often mentioned but unclear how they were determined and often difficult to appreciate from images, more quantitative analyses should be carried out over the whole trajectory rather than just some individual frames (plots of time evolution of interactions, occupancy etc)

The question of evaluating and quantifying interactions over time is addressed in the 3rd major point. All kinds of atom contacts were determined by visual inspection of the entire models, measuring the distances between atoms to assess for polar bonds and H-bonds. This was executed on static structures and along the MD simulations. We did not try to make an exhaustive description of possible conformations of the IHM or the thick filament.

The movies presented to illustrate this point (Supplementary Movie 7, 13-15) were made with representative frames chosen after a detailed analysis of the entire trajectories and of the evolution of the interfaces frame by frame (sampling 0.1 ns per frame as indicated in the "Methods"). A complete list of the residues found in interfaces in long term simulations is provided in Supplementary Table 4 (colored in brown). The "Methods" were edited to clarify this point and reads as follows:

(p. 24-25, l. 895-906) **"Trajectory analysis**

Trajectories were generated and processed using "gmx trajconv" from Gromacs. Frames of 0.1 ns steps were extracted from the original simulations (sampled at 10ps) for the analysis. All the trajectories were adjusted on the first frame by a superimposition on the backbone of the motor domains (1-710) for $_{Car}$IHMs and on the backbone of MyBP-C, Titin and the contextual coiled-coils in the filament, using the macrocommand of VMD (RMSD trajectory tool, version 1.9.4a53, June 29, 2021), this allowed the extraction of the RMSD plots. RMSF were obtained with MDAnalysis[57] and further used to compute the sausage "putty" representations[58] in PyMOL.

All trajectories were analyzed in detail to describe the time evolution of the interfaces. For each interface presented, representative frames illustrating this evolution were selected and assembled into movies (**Supplementary Movies 7, 13–15**). Interactions observed in the molecular dynamics simulations but not present in the initial models are highlighted in brown in **Supplementary Table 4**."

- The flexibility difference between WT and E525K shown via sausage plots and colour gradient in Supplementary Figure 9 is not very evident (the two images look very similar in terms of flexibility), other types of representations might be more suitable. This image is used in the text to show how MD results agree with CryoEM ones ("The flexibility observed for each residue in the simulations (Supplemental Fig. 9B-D) is consistent with the CryoEM data (Supplemental Fig. 7B, Movie 1)"), but at the moment this is not really shown: the mentioned CryoEM data are about flexibility of FH, which is not clearly shown in Supplementary Figure 9.

When we state that the flexibility observed in molecular dynamics simulations is consistent with cryoEM data, it means that regions of flexibility in cryoEM are also found to be flexible *in silico*. When ConfA and ConfB are compared, different interactions are found in the cryoEM maps for interactions between the heads. These regions are also dynamic in the MD simulations and similar interactions occur. For E525K, the S2 is stabilized compared to WT both in MD simulations and in cryoEM maps. We agree that **Supplementary Fig. 9A** was not sufficient to illustrate this concept. Another analysis has been added to Supplementary Fig. 9, the RMSF was plotted for each condition (BH and FH) and the location of the S2 coiled-coil is indicated (**Supplementary Fig. 9B**). This shows that the S2 is stabilized in E525K compared to WT and that it is significantly destabilized in the E903K condition. To clarify our statements, the sentence now reads as follows:

(p.8 , l. 269-272) "In detail, the *in silico* and CryoEM results are also consistent and complementary. The flexibility observed for each residue in the MD simulations (**Supplementary Fig. 9C-E**) matches the conformational variability captured by CryoEM data (**Supplementary Fig. 7B**, **Supplementary Movie 1**)." The MD simulations further allow visualization of the specific interactions that can occur at the $_{Car}$IHM interfaces over time (**Supplementary Movies 5 & 6**). In particular, the interactions established between ^{FH}Loop-2 and the S2 illustrate perfectly the concept of "musical chairs": the flexible ^{FH}Loop-2 forms a dynamic interface through a network of transient polar interactions. The transient nature of these interactions, combined with the flexibility of ^{FH}Loop-2, makes it possible to maintain the global interface despite changes in the orientation of S2 (**Supplementary Movie 7**).

- The following statement is unclear: "The importance of the asymmetric S2 bend (Cluster 0) in the stabilization of a unique asymmetric $_{Car}$IHM structure is highlighted by the number of missense mutations that can result in cardiomyopathies (Fig. 4C, Supplemental Fig. 12)." The mutations shown seem to be evenly distributed throughout the coiled coil, what information is this giving us? Is this distribution any different from the one observed in the motor domains for example? How can a link be drawn between the distribution shown and the importance of the asymmetric bend?

The interesting aspect of this region is that it is not involved in an interface. Instead, its structural features appear to be important and require a specific sequence, as several missense mutations are present here. If the coiled coil in this region would remain symmetric (as found in the crystal structure PDB 2FXO), many residues would face the solvent and should not impair myosin function or regulation. In our high-resolution structures, we indicate that the coiled-coil here forms asymmetric bonds due to the presence of an unusual motif 'RRK' in the coiled-coil that involves residues that do not interact in a symmetrical manner. The interactions that can form likely stabilize the S2 coiled coil in a bent conformation that helps it find the BH. We thank the reviewer for having identified this sentence that could lead to misunderstanding. The text was modified:

(p. 11, l. 378-381) "The fact that missense mutations on the asymmetric S2 bend (Cluster 0) can result in cardiomyopathies (Fig. 4C, Supplemental Fig. 12) indicates that this region is not a simple connector that can be modified at will, but a stabilizing element of the asymmetric $_{Car}$IHM structure."

- "Interestingly, the HCM MYH7 E45 mutations to Asp, Gly, or Lys, classified as variants of unknown significance (VUS) for HCM, belong to this category (to date, no mutations of E45 are officially ascertained as pathogenic/likely pathogenic). Our study indicates that this residue is critical for the formation of the intercrown contacts (Movie 14)." Residue E45 does not seem to be shown in the movie.

This residue is part of the interface in the atomic model of the filament. The interaction is illustrated in Fig. 6C. Now the text reads as follows:

(p. 17, l. 569-572) "Interestingly, the HCM MYH7 E45 mutations to Asp, Gly, or Lys, classified as variants of unknown significance (VUS) for HCM, belong to this category (to date, no mutations of E45 are officially ascertained as pathogenic/likely pathogenic). Our study indicates that this residue is critical for the formation of the intercrown contacts (**Fig. 6C**)"

Reviewer #3 (Remarks to the Author):

I co-reviewed this manuscript with one of the reviewers who provided the listed reports. This is part of the Nature Communications initiative to facilitate training in peer review and to provide appropriate recognition for Early Career Researchers who co-review manuscripts.

Reviewer #1 (Remarks to the Author):

The authors addressed all my points, and I can recommend the paper for publication in its current form.

We thank Reviewer #1 for his comments and positive appreciation of our work.

Reviewer #2 (Remarks to the Author):

We appreciate the effort made to address all comments. Few minor remaining points:
We thank Reviewer #2 for his comments to improve the quality of our work. They all have been addressed as follows.

** Movie 5 still cannot be played*

We re-recorded Movie 5 to be sure it can be displayed properly. On our computers, we are able to open it in different movie players.

** "A transient interaction between FHK903 and BH 291 E653 occurs, but S2 continues to explore positions far from 292 those of WT-CarIHM. The orientation and flexibility of the proximal S2, below the Hook and prior to 293 Cluster 1, are also significantly affected (Supplementary Fig. 9D)." Probably Supplementary Fig. 9E (not D) should be mentioned at the end of the sentence*

We thank Reviewer #2 for this comment. This was changed in the last version.

** Ideally reproducibility across replicas should be confirmed for all the main findings.*

We included additional information that confirms the reproducibility of all the main findings. Supplementary Dataset 2 includes the raw data (i) for the RMSD of the two replicates and (ii) for the distances between residue 525 and negative charges of the S2 (D896, D900, E902, E903) during the time course of the simulation for the three conditions (WT, E525K and E903K). Supplementary Datasets 3 and 4 contain the raw data for the RMSD and the RMSF calculated from the two replicates of the molecular dynamics simulations of the cardiac relaxed filament. Here again, two replicates are included, showing the reproducibility of our observations.

The main text and the "Methods" sections were edited as follows:

*"Raw data from the simulations and their replicates are provided in **Supplementary Dataset 1.**"* *(p.8, l.258-259)*

"*Raw data from the replicates of this simulation are provided in **Supplementary Datasets 2–4**."(p.16, l.526-527)*

"Each simulation was performed twice, to ensure the reproducibility of the observations (see **Supplementary Dataset 1**). The two sets of simulations lead to the same conclusions (i) E903K is more dynamic than WT and E525K, and (ii) the mutations alter the charge network of Cluster 2, with K525 stabilizing the coiled-coil position by establishing electrostatic interactions with negatively charged residues located on the S2 (D896, D900, E902, E903) (**Supplementary Dataset 1**). These distances show how interactions between K525 and negatively charged residues on S2 are maintained during the E525K simulations. " (p.24 l. 839-844).

"The simulations were performed twice to ensure the reproducibility of the results, with the "flexible interfaces" (loop 686-711 from the C5 domain of MyBP-C; myosin Loop-1 (residues 200-215) or the N-terminal extension of the RLC) rebuilt independently in the duplicate (**Supplementary Fig. 16**, **Supplementary Dataset 2 & 3**). Raw RMSD and RMSF data from both simulations are provided in **Supplementary Datasets 2 and 3**. **Supplementary Dataset 4** presents the interactions established at the intercrown interface over the course of two independent molecular dynamics simulations. Although these interactions are not strictly identical at each time point, most interacting residues are conserved throughout the simulations, supporting their reproducibility. Similarly to isolated $_{Car}$IHM, both molecular dynamics simulations of the filament lead to the same conclusions. " (p. 25, l. 903-909).

The legends of the Supplementary Datasets were added in the document of Supplementary Material:

"**Supplementary Dataset 1 – Raw data of the molecular dynamics simulations experiments of isolated $_{Car}$IHM.** For the three conditions (WT, E525K, and E903K), raw RMSD values as a function of time are provided and plotted ("Raw RMS"). Distances between residue 525 and neighboring negatively charged residues of the S2 coiled-coil are also calculated as a function of time for the three conditions ("Dist"). For each condition, two independent simulations were performed (see Methods), and raw data from the replicates are shown here, demonstrating the reproducibility of the phenomena described in the text.

Supplementary Dataset 2 – Raw data of the molecular dynamics simulations experiments of relaxed thick filament. For each chain, the RMSD data are shown as function of time in the two independent replicates. Only replicate 1 is discussed in the text, but the data shown here demonstrate' the reproducibility of the phenomena described.

Supplementary Dataset 3 – Raw data of the molecular dynamics simulations experiments of relaxed thick filament. For each chain, the RMSF data are shown as function of time in the two independent replicates. Only replicate 1 (“Traj1”) is discussed in the text, but the data shown here demonstrate' the reproducibility of the phenomena described.

Supplementary Dataset 4 Intercrown interface during all-atom molecular dynamics simulations 1 and 2 of the relaxed thick filament. ”
